# Gamified Web-Delivered Attentional Bias Modification Training for Adults With Chronic Pain: Randomized, Double-Blind, Placebo-Controlled Trial

**DOI:** 10.2196/50635

**Published:** 2025-01-16

**Authors:** Julie F Vermeir, Melanie J White, Daniel Johnson, Geert Crombez, Dimitri M L Van Ryckeghem

**Affiliations:** 1 School of Psychology and Counselling Faculty of Health Queensland University of Technology (QUT) Brisbane Australia; 2 School of Computer Science Faculty of Science Queensland University of Technology (QUT) Brisbane Australia; 3 Department of Experimental Clinical and Health Psychology Ghent University Ghent Belgium; 4 Department of Clinical Psychological Science Maastricht University Maastricht Netherlands; 5 Department of Behavioural and Cognitive Sciences University of Luxembourg Esch-sur-Alzette Luxembourg

**Keywords:** chronic pain, cognition, attentional bias, gamification, motivation, randomized controlled trial, web-based intervention, pain management, digital intervention, digital health

## Abstract

**Background:**

Attentional bias to pain-related information has been implicated in pain chronicity. To date, research investigating attentional bias modification training (ABMT) procedures in people with chronic pain has found variable success, perhaps because training paradigms are typically repetitive and monotonous, which could negatively affect engagement and adherence. Increasing engagement through the gamification (ie, the use of game elements) of ABMT may provide the opportunity to overcome some of these barriers. However, ABMT studies applied to the chronic pain field have not yet incorporated gamification elements.

**Objective:**

This study aimed to investigate the effects of a gamified web-delivered ABMT intervention in a sample of adults with chronic pain via a randomized, double-blind, placebo-controlled trial.

**Methods:**

A final sample of 129 adults with chronic musculoskeletal pain, recruited from clinical (hospital outpatient waiting list) and nonclinical (wider community) settings, were included in this randomized, double-blind, placebo-controlled, 3-arm trial. Participants were randomly assigned to complete 6 web-based sessions of nongamified standard ABMT (n=43), gamified ABMT (n=41), or a control condition (nongamified sham ABMT; n=45) over a period of 3 weeks. Active ABMT conditions trained attention away from pain-related words. The gamified task included a combination of 5 game elements. Participant outcomes were assessed before training, during training, immediately after training, and at 1-month follow-up. Primary outcomes included self-reported and behavioral engagement, pain intensity, and pain interference. Secondary outcomes included anxiety, depression, cognitive biases, and perceived improvement.

**Results:**

Results of the linear mixed model analyses suggest that across all conditions, there was an overall small to medium decline in self-reported task-related engagement between sessions 1 and 2 (*P*<.001; Cohen *d*=0.257; 95% CI 0.13-0.39), sessions 1 and 3 (*P*<.001; Cohen *d*=0.368; 95% CI 0.23-0.50), sessions 1 and 4 (*P*<.001; Cohen *d*=0.473; 95% CI 0.34-0.61), sessions 1 and 5 (*P*<.001; Cohen *d*=0.488; 95% CI 0.35-0.63), and sessions 1 and 6 (*P*<.001; Cohen *d*=0.596; 95% CI 0.46-0.73). There was also an overall small decrease in depressive symptoms from baseline to posttraining assessment (*P*=.007; Cohen *d*=0.180; 95% CI 0.05-0.31) and in pain intensity (*P*=.008; Cohen *d*=0.180; 95% CI 0.05-0.31) and pain interference (*P*<.001; Cohen *d*=0.237; 95% CI 0.10-0.37) from baseline to follow-up assessment. However, no differential effects were observed over time between the 3 conditions on measures of engagement, pain intensity, pain interference, attentional bias, anxiety, depression, interpretation bias, or perceived improvement (all *P* values>.05).

**Conclusions:**

These findings suggest that gamification, in this context, was not effective at enhancing engagement, and they do not support the widespread clinical use of web-delivered ABMT in treating individuals with chronic musculoskeletal pain. The implications of these findings are discussed, and future directions for research are suggested.

**Trial Registration:**

Australian New Zealand Clinical Trials Registry (ANZCTR) ACTRN12620000803998; https://anzctr.org.au/ACTRN12620000803998.aspx

**International Registered Report Identifier (IRRID):**

RR2-10.2196/32359

## Introduction

### Background

Cognitive theories of pain posit that biases in attention contribute to the development and maintenance of chronic pain problems [1-3]. For example, the fear-avoidance model of chronic pain [4] assigns a causal role to attentional bias, such that individuals who have an attentional bias are more likely to have higher levels of pain and pain-related disability and, subsequently, be at a greater risk of developing chronic pain. Several reviews and meta-analyses have found that individuals with chronic pain exhibit an attentional bias toward pain-related words or pictures [5-7], and these biases have been positively associated with pain intensity, pain-related disability, and emotional distress [8,9]. This has led research to explore whether these attentional biases can be directly altered using a computer-based attentional bias modification training (ABMT) program and whether this modification results in concomitant changes in pain intensity and health outcomes associated with pain [10]. ABMT protocols typically use a modified version of the dot-probe task [11] to alter the bias by training individuals to disengage from pain-related cues and redirect attention to the competing non–pain-related (neutral) cues. It is suggested that repeated ABMT trials create a strategy for individuals to disengage from pain-related information and to facilitate attentional engagement toward other non–pain-related information. If this strategy is transferred to everyday life, it is expected to result in a reduction of pain, pain interference, and disability [10,12].

ABMT has been proposed as a promising tool for chronic pain based on its successful use for various conditions such as anxiety [13] and depression [14]. So far, however, results of pain ABMT interventions in pain (refer to the study by Van Ryckeghem et al [15] for an overview) and chronic pain samples [10,12,16-20] have been mixed, with most studies reporting at least some short- to medium-term therapeutic benefits of ABMT for clinically relevant pain outcomes [10,12,16,18,19].

A total of 2 factors that may contribute to these mixed findings are boredom and low motivation. ABMT procedures require participants to complete numerous trials over multiple sessions across several weeks [10,12,16-20] and typically use a basic layout. There is evidence that participants view the dot-probe task as monotonous, repetitive, and boring [21-23]. Consequently, this may lead to (temporal) low task engagement, low motivation to complete the sessions, and high dropout rates, which in turn may compromise the effectiveness of the intervention. Increasing engagement through the gamification of ABMT may provide the opportunity to overcome some of these barriers. Gamification refers to the use of digital game elements (eg, points) in nonentertainment settings [24]. Several reviews on gamified cognitive training tasks have reported that adding game-like elements to repetitive tasks improves motivation and engagement [25,26]. This is further supported by a recent study investigating gamification of interpretation bias modification for anxiety, which found that gamification could increase engagement and enjoyment [27]. However, it is still difficult to draw any firm conclusions regarding the effectiveness of gamified cognitive bias modification interventions [27,28], and more rigorously designed and theory-driven research is needed, particularly in the field of chronic pain.

### Aims and Hypotheses

The aim of this study was to investigate the effects of a gamified web-delivered ABMT intervention using an empirically supported set of pain-related word stimuli on behavioral and self-reported engagement, pain intensity, pain interference, anxiety, depression, cognitive biases, and perceived improvement in a sample of adults with chronic musculoskeletal pain. We hypothesized that (1) the gamified ABMT condition would be more engaging than the nongamified (ie, standard ABMT and control) conditions; (2) both the standard and gamified ABMT conditions, compared to the control condition, would be more effective in improving outcomes of interest over time; and (3) these improvements would be greater in the gamified ABMT condition compared to the standard ABMT condition [29].

## Methods

The trial protocol has been published elsewhere [29]. This study is conducted and described according to the CONSORT-EHEALTH (Consolidated Standards of Reporting Trials of Electronic and Mobile Health Applications and Online Telehealth) checklist (Multimedia Appendix 1) [30].

### Ethical Considerations

The study was approved by the Human Research Ethics Committees of the Royal Brisbane and Women’s Hospital (HREC/2020/QRBW/61743) and Queensland University of Technology (2000000395) and prospectively registered on the Australian New Zealand Clinical Trials Registry (ACTRN12620000803998). All participants provided informed consent before their inclusion in the study, with the option to withdraw at any time without any consequences. Only approved study team members had access to participant data. The data were deidentified before analysis to safeguard participants’ privacy. No incentives were offered to the participants.

### Study Design and Setting

This study was a randomized, double-blind, placebo-controlled, 3-arm, parallel-group trial examining the efficacy of a gamified web-delivered ABMT for chronic pain. Participants were involved in the study for approximately 2 months, which included a 3-week intervention period, followed by a 1-month follow-up period. All training sessions were conducted via the internet at the participants’ time and place of convenience using their own computers, and all outcome assessments (ie, at baseline, during training, immediately after training, and at 1-month posttraining) were self-assessed via web-based questionnaires and computerized tasks. Participants were randomly allocated to 1 of the 3 training conditions: nongamified standard ABMT, gamified ABMT, or nongamified sham ABMT (control). The control condition comprised a dot-probe paradigm without training direction (ie, the probe was located in the position of the pain-related vs non–pain-related words with equal probability), whereas the standard and gamified ABMT conditions aimed to train attention away from pain-related words (ie, the probe was located in the non–pain-related word location most of the time). Those in the control condition were offered the opportunity to do the standard ABMT at the conclusion of the study. Concomitant care (eg, rehabilitation program and pain medications) was permitted during the trial and was monitored through a pain treatment question that probed participants’ pain treatments and frequency since the commencement of the study or previous assessment. Electronic informed consent (ie, e-consent) was obtained for all study participants.

### Participants

Participants were recruited from a large Australian public hospital outpatient waitlist for pain management (clinical setting) and from the wider community (nonclinical setting). Individuals on the hospital outpatient waiting list were invited to participate through personalized mail correspondence, whereas individuals from the wider community were recruited through university electronic mailing lists, social media, and community channels (eg, Facebook advertising, Pain Australia, Chronic Pain Australia, and word of mouth). The inclusion and exclusion criteria for participants are listed in Textbox 1.

**Eligibility criteria.**

**Inclusion criteria**

- Aged ≥18 years
- Experiencing chronic musculoskeletal pain, that is, pain in bones, joints, muscles, or related soft tissues (eg, rheumatoid arthritis pain, nonspecific back pain, or fibromyalgia pain)
- Meeting the criteria for chronic pain, that is, self-reported pain that lasts or recurs for >3 months [31]
- Having normal or corrected-to-normal (eg, glasses or contact lenses) vision

**Exclusion criteria**

- Not being a native English speaker or fluent in reading and writing English (as participants’ reaction time to English words was used as an index of attentional bias to semantically related pain memory networks)
- Not having access to a desktop or laptop computer connected to reliable internet (as the trial was conducted on the web)
- Not being able or willing to provide informed consent to participate

As this was the first study to assess the effects of gamification techniques in a pain ABMT intervention, no prior effect size was available to guide sample size estimation during the study design phase. Therefore, a minimum sample size of 30 per training condition was planned on the basis that this exceeded the sample size determined by several similar pain ABMT and gamified training studies [12,20,32]. Considering attrition rates of previous trials in chronic pain treatment [33] and given the 1-month follow-up assessment, a dropout rate of approximately 30% was expected for this trial. Therefore, a total target sample size of 120 participants (40 participants per condition) was sought. We monitored attrition throughout the study, and recruitment ended when approximately 120 participants had completed at least 1 training session (ie, minimum threshold for exposure to ABMT).

### Randomization, Allocation Concealment, and Blinding

After the baseline assessment, eligible consenting adults were randomly allocated to 1 of the 3 training conditions: standard ABMT, gamified ABMT, or control. Participant randomization was performed by an independent researcher with no involvement in the study using a computerized random number generator, Sealed Envelope (Sealed Envelope Ltd). A block randomization technique was used, allowing 6 participants at a time to be randomized in equal proportions to the 3 training arms. Participants and researchers were both blinded to the training condition to which participants were assigned. Participants were not provided information about the 3 training conditions but were informed that they might receive the intervention or complete a similar task (the control condition). This approach kept participants blinded to their allocation, as it would have been easy for them to recognize if they were in the active gamified condition (particularly because the control condition did not include game elements). The outcome data were blinded, as assessments and training occurred on the web in the absence of the investigators. The first author (JFV) could access the training data to monitor the data collection process and was responsible to respond to participants who had questions or technical issues. However, it is unlikely that this caused problems of bias allocation or assessment because of the web-based nature of the study.

### Study Program

#### Task Stimuli

Word stimuli were taken from a large pool of pain-related and non–pain-related linguistic stimuli, previously created and evaluated for use in chronic pain samples [34]. Specifically, for this study, we selected sensory and affective pain words that were rated as most related to chronic musculoskeletal pain and were categorized the fastest as pain-related by adults with self-reported chronic pain. As shown in Table 1, three sets of word stimuli were used: (1) 8 non–pain-related word pairs related to the categories of natural and man-made resources for the practice trials, (2) 8 pain-related and non–pain-related word pairs for the training trials, and (3) 8 pain-related and non–pain-related word pairs for the pretraining and posttraining assessment of attentional bias trials. Each pain-related word was matched with a non–pain-related word for length and frequency of word use in the English language, according to SubtlexUS [35]. Word stimuli in each set were not replicated in any other set, and each word stimulus was presented in a black 28-point uppercase Courier New font on a white background.

**Table 1:** Word pairs used in the practice, training, and assessment trials.

Practice set		Training set		Assessment set	
Non–pain-related	Non–pain-related	Pain-related	Non–pain-related	Pain-related	Non–pain-related

Cork	Fork	Aching (S^a^)	Sponge	Agonizing (A^b^)	Octagonal
Cotton	Carpet	Burning (S)	Streets	Cramping (S)	Cupboard
Goat	Lamp	Debilitating (A)	Strawberries	Cutting (S)	Crystal
Iron	Huts	Pain (S)	Bird	Excruciating (A)	Entertaining
Log	Pot	Stabbing (S)	Stocking	Hurting (S)	Trailer
Plant	Table	Suffering (A)	Newspaper	Sharp (S)	Plate
Rice	Bowl	Throbbing (S)	Resorting	Spasm (S)	Tiles
Waterfalls	Microwaves	Unbearable (A)	Metabolite	Tortured (A)	Currents

^a^S: sensory pain-related word.

^b^A: affective pain-related word.

#### Experimental Tasks

##### Overview

Tasks were programmed and presented using Inquisit 6.4 (Millisecond Software) on participants’ internet-connected computers. To account for different screen sizes and ensure consistency in the display of word stimuli across participants, a calibration process was performed at the start of each session, asking participants to place a credit card on the screen and adjust the length of a horizontal line until it matched the width of the credit card.

The 3 training tasks were delivered using modified versions of the dot-probe task [11]. Each task began with a centered fixation cross for 500 milliseconds, after which a randomly selected stimulus pair of words (ie, a pain-related and a non–pain-related word) was presented horizontally on the screen for 500 milliseconds, with one word located at the top of the screen and the other at the bottom. Next, the paired words disappeared, and a probe (*p* or *q*) replaced the location of 1 of the stimuli. Participants were instructed to determine whether a *p* or a *q* had appeared and respond as quickly and as accurately as possible by pressing either the *P* or *Q* key on the keyboard, with the right and left index finger, respectively. The probe disappeared after 2500 milliseconds or sooner upon response. The intertrial interval was 500 milliseconds.

In addition, digit trials, that is, trials during which a random digit number between 1 and 9 replaced the fixation cross for a duration of 150 milliseconds, were included to ensure that participants’ attention was directed to the center of the screen [20,36]. The intertrial interval was 1000 milliseconds after digit trials so that participants could reposition their index fingers on the *P* and *Q* keys. In the context of this study, trials in which the probe appeared in the location previously occupied by the pain-related word were considered congruent trials, and trials in which the probe appeared in the opposite location to that previously occupied by the pain-related word were considered incongruent trials.

##### Nongamified Standard ABMT

Each session started with a practice block of 17 trials (16 non–pain-related stimulus pairs and 1 digit trial) where participants received feedback after every correct (ie, *Correct!*) and erroneous (ie, *Incorrect!*) response. The training phase comprised 4 training blocks, each comprising 68 experimental trials (8 congruent trials, 56 incongruent trials, and 4 digit trials), totaling 272 experimental trials. The probe replaced non–pain-related cues in 87.5% (224/256) of trials and pain cues in 12.5% (32/256) of trials, thereby directing attention away from pain-related words. This distribution was selected to reduce the obviousness of the probe contingency [37], and participants were not made aware of it. Word pairs were randomly presented in each of the 4 possible combinations (probe up and target down, probe down and target up, probe up and target up, and probe down and target down). Stimuli were presented in a randomized order across trials and participants, and trials were intermixed and randomly presented in 4 blocks, with a rest offered between each block of trials.

##### Nongamified Sham (Control) ABMT

The control and standard ABMT conditions were identical in all respects, except that in the control condition, the probe appeared with equal frequency in the position of the pain-related and non–pain-related words.

##### Gamified ABMT

Gamified ABMT was based on the standard ABMT but with the addition of game elements. Details regarding the development of the gamified task can be found in the published study protocol [29]. In brief, the development of gamified ABMT followed the Medical Research Council framework for complex interventions [38], incorporating theory, evidence from reviews, and expert input. The selection of game elements was guided by concepts of self-determination theory [39,40] and self-regulation [41] and informed by a recent qualitative and quantitative review assessing the effectiveness of gamification applied to cognitive training tasks [25]. According to self-determination theory [39,40], competence, relatedness, and autonomy are the 3 basic psychological needs that determine intrinsic motivation, sustained engagement, and psychological well-being. Self-regulation techniques such as goal setting and self-monitoring can also motivate users to engage and sustain in activities [42-44].

**Figure 1:**
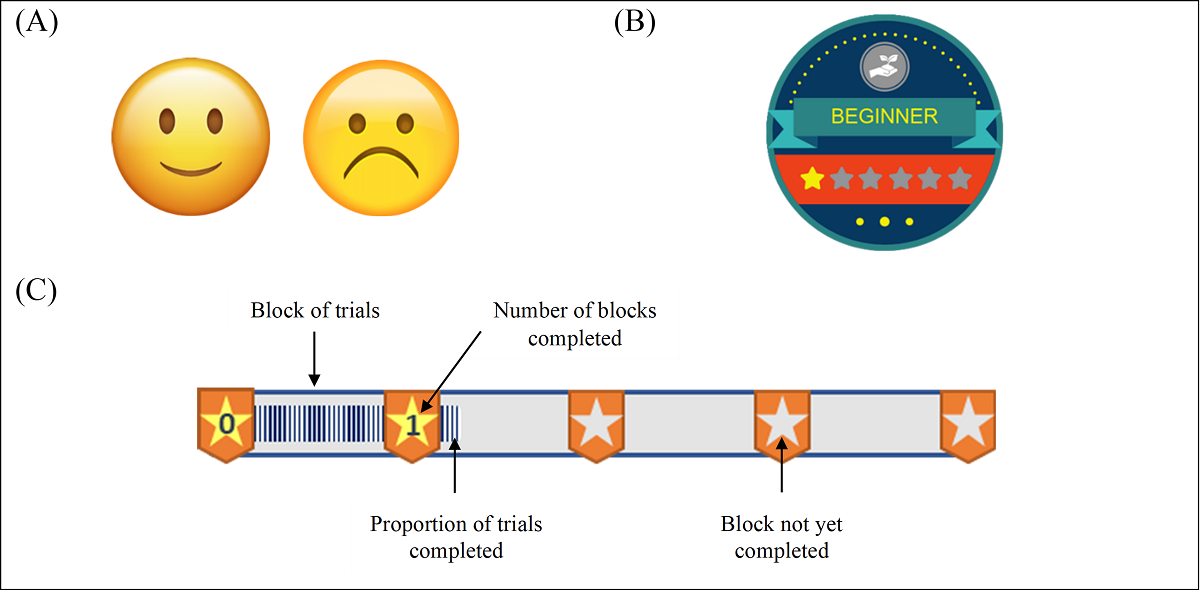
Sample of game elements used in the gamified task: (A) smiling (left) and frowning (right) emoticons received during practice trials for correct and incorrect responses, respectively; (B) badge earned at the end of the first training session; and (C) progress bar representing the proportion of trials completed in each block and a written indicator reflecting the number of blocks completed.

Specifically, a combination of 5 game elements was incorporated in the gamified task to facilitate participants’ motivation and engagement in the ABMT procedure. These features were specifically chosen and implemented in a way designed to minimize cognitive disruption (ie, aiming to avoid interfering with the key cognitive mechanisms involved in the procedure). First, at the beginning of each training session, a clear gamified performance goal was set for the task: to earn as many points as possible and receive badges along the way (game element: clear gamified goal). Goals that are specific and reasonably challenging are the most effective at increasing motivation and task performance [45] and are likely to increase the satisfaction of the need for competence [46]. Second, during the practice phase, immediate gamified feedback was given (game element: feedback loops). For each correct trial, the word *Correct!* and a smiling emoticon appeared on the screen, whereas the word *Incorrect!* and a frowning emoticon occurred in every incorrect practice trial (Figure 1). This type of feedback has been shown to facilitate self-monitoring [43,47] and feelings of competence [46]. Third, during the training phase, a constantly visible progress bar at the top of the screen indicated the proportion of trials remaining in each block, and a written indicator reflected the number of blocks completed (game element: task-related progress; Figure 1). Such gamification features have been shown to facilitate self-tracking and motivate participants toward the attainment of goals [42,43] and fulfill their desire for competence [48]. Fourth, between blocks of trials, participants received feedback about their performance in the form of points (game element: rewards), calculated for each block of trials (1 point is earned for each correct trial; maximum of 68 points could be earned per block). To ensure the flow of training was uninterrupted, feedback was provided after each block of trials rather than after each trial. In addition, an auditory and visual reward in the form of a firework was incorporated into the task (game element: sound effect with reward). All the participants in the gamified ABMT condition experienced the fireworks after the first block of trials to ensure that everyone was exposed to the same type of game elements. However, for subsequent blocks of trials, only those who obtained at least 60% accuracy experienced the fireworks. Audio-visual rewards have been shown to emphasize feelings of competence [49], and the criterion of 60% involves an element of uncertainty that could further increase motivation. Finally, at the end of each training session, the participants were rewarded with a badge (game element: rewards). There were 6 different badges, and each badge had a number on it corresponding to the number of sessions completed (Figure 1). Collectible points and badges have been shown to facilitate goal setting [42,43] and satisfy the needs for competence, autonomy, and relatedness [46].

### Outcomes and Measures

Data were collected via the internet using the web-based system Qualtrics (Qualtrics International Inc) for survey responses and Inquisit 6.4 (Millisecond Software) for task data and self-reported engagement responses. Participants’ outcomes were assessed before beginning training (baseline), during training (for self-reported task-related engagement), immediately after completion of training (posttraining), and 1 month after the last training session (follow-up).

#### Baseline Information

At baseline, demographic information (eg, age); health data (eg, general health status); pain experience details (eg, duration of primary pain condition); and data on type of computer, keyboard, and screen size used were collected. To provide further information about the participants’ chronic pain severity, the Graded Chronic Pain Scale (GCPS) [50] was administered. The GCPS is a 7-item self-report instrument that assesses pain intensity and pain-related disability in the past 6 months. All items, except for the number of days disabled, are scored on an 11-point Likert scale, with responses ranging from 0 to 10. Subscale scores for the pain intensity and pain-related dimensions are combined to calculate a chronic pain grade that enables individuals to be classified into pain severity grades ranging from grade 0 (*no pain problem*) to grade 4 (*high disability*-*high intensity*). The reliability and validity of the GCPS have been demonstrated in previous studies [50-52].

#### Primary Outcome Measures

##### Engagement

A total of 2 self-report measures were used to assess participants’ experiences of engagement. Task-related engagement was measured after each training session with a single-item question: How engaging was this session? This item was rated on an 11-point Likert scale, ranging from 0 (*not at all*) to 10 (*very much*). Higher scores represent greater engagement. Task-related interest and enjoyment was assessed with the Intrinsic Motivation Inventory (IMI) Interest and Enjoyment subscale [53-55], which comprises 7 items. Each item is scored on a 7-point Likert scale that ranges from 1 (*not at all*) to 7 (*very true*), with higher scores representing higher levels of interest and enjoyment. The reliability and validity of this subscale have been demonstrated in previous studies [56,57].

A total of 2 behavioral measures were used to assess participants’ engagement: nonuse intervention attrition [58], defined as the number of participants who discontinued using the intervention at each training session, and completion rates, defined as the number of training sessions (out of 6) that each participant completed during the intervention period.

##### Pain Intensity

The Patient-Reported Outcomes Measurement Information System (PROMIS) Pain Intensity Short Form 3a (version 1.0; 3 items) [59] was used to measure pain intensity. The first 2 items assess pain intensity over the last 7 days (average and worst pain), and the last item assesses pain intensity *right now*, each scored using a 5-point Likert scale, with responses ranging from 1 (*had no pain* or *no pain*) to 5 (*very severe*). As recommended by PROMIS, the Pain Intensity scale (version 1.0) was rescored into T-scores by using the free web-based HealthMeasures Scoring Service [59]. Higher T-scores represent worse pain. This measure has shown to be valid for assessing pain intensity in various settings [60].

##### Pain Interference

The PROMIS Pain Interference Short Form 8a (version 1.0; 8 items) [59] was used to assess the impact of pain on daily life over the last 7 days using a 5-point Likert scale, with responses ranging from 1 (*not at all*) to 5 (*very much*). As recommended by PROMIS, the Pain Interference scale (version 1.0) scale was rescored into T-scores using the free web-based HealthMeasures Scoring Service [59]. Higher T-scores represent greater pain interference. This measure has been assessed and validated in both general and clinical populations [60,61].

#### Secondary Outcome Measures

##### Measure of Attentional Bias for Pain

The dot-probe paradigm [62] was used to measure pain-related attentional biases. This task is similar to the nongamified sham (control) ABMT task, except for the total number of trials. The assessment phase included a practice block of 17 trials (16 non–pain-related stimulus pairs and 1 digit trial) and 2 assessment blocks of 68 trials (32 congruent trials, 32 incongruent trials, and 4 digit trials), totaling 136 assessment trials. Stimuli were presented in a randomized order across trials and participants, and trials were intermixed and randomly presented in 2 blocks, with a rest offered between the blocks.

##### Anxiety and Depression

A total of 2 PROMIS measures comprising PROMIS Anxiety 8a (version 1.0; 8 items) and PROMIS Depression 8b (version 1.0; 8 items) [59] were used to assess negative affect over the last 7 days using a 5-point Likert scale, with responses ranging from 1 (*never*) to 5 (*always*). The scales were scored using the free web-based HealthMeasures Scoring Service [59]. Higher T-scores represent greater symptoms of anxiety or depression. These PROMIS measures have demonstrated excellent psychometric properties in both population-based [63] and clinical samples [64,65].

##### Perceived Improvement

The Patient Global Impression of Change (PGIC) scale [66] was used to assess participants’ perception of overall pain-related improvement following training. It is composed of a single item rated on a 7-point Likert scale, ranging from 1 (*very much improved*) to 7 (*very much worse*). For descriptive purposes, participants were classified into 3 categories according to the PGIC score: disease deterioration (1-3 points), stable disease (4 points), or disease improvement (5-7 points) since the start of the program [67]. The PGIC is widely used in chronic pain research [67,68].

##### Measure of Interpretation Bias for Pain

An adapted version of the computerized interpretation bias task [69] was used to measure pain-related interpretation biases, which contains 16 incomplete vignettes that describe 8 ambiguous situations relating to bodily threat or pain and 8 ambiguous situations relating to social evaluations. Vignettes were adapted to reflect events that may occur in the workplace, home, or during an adult’s everyday life. Participants are asked to rate how likely each ending came to their mind on a scale of 1 (*does not come to mind*) to 5 (*definitely comes to mind*) and to select the interpretation (word) that first came to their mind. Next, participants are presented with the same scenarios again; however, this time, they are asked to rate the likelihood that each resolution would actually happen in that situation on a scale of 1 (*not likely*) to 5 (*very likely*) and to select the word that they believe is most likely to end the sentence. All items and interpretations were presented in a fixed random order.

Similar to previous reports [69-71], this study only used the rating data for interpretation belief (ie, belief that the interpretation is likely to be true); however, all other data are available upon request from the authors. An interpretation bias score for each domain was calculated by subtracting the mean ratings of benign endings from the mean ratings of negative endings, with higher scores indicating a higher tendency to believe that negative interpretations are likely to be true. Previous studies using this task have found evidence of interpretation biases for pain in individuals with chronic pain, particularly for interpretation belief [70].

#### Exploratory and Other Measures

##### Exploratory Measures

As detailed in the preregistered protocol, further measures were collected for exploratory purposes, all of which are reported in more detail in Multimedia Appendix 2 [20,29,72-77]. These measures included the Attentional Control Scale (ACS) [72], the Behavioral Inhibition System and Behavioral Activation System scales [73], and the Pain Catastrophizing Scale [74].

##### Pain Treatment Information

The posttraining and 1-month follow-up assessments included a question that probed participants’ use of pain treatments and frequency of health care use since the commencement of the study or previous assessment.

##### Manipulation Check

The posttraining assessment included a manipulation check question asking participants whether they believed they had received the intervention or sham training (ie, no intervention).

##### Validity Check

Instructional questions (eg, “Please select 5=Always”) were included in the baseline, posttraining, and 1-month follow-up assessments to identify careless responding patterns [78]. Participants were excluded if they answered all instructional questions incorrectly.

### Procedure

Interested participants first provided informed consent before being taken to the screening questions, and then to the baseline assessment (approximately 35 min), consisting of questions relating to demographic characteristics; general health status; current mental health status; pain experience information; as well as the PROMIS Pain Intensity 3a, PROMIS Pain Interference 8a, PROMIS Anxiety 8a, PROMIS Depression 8b, ACS, Behavioral Inhibition System and Behavioral Activation System scales, and Pain Catastrophizing Scale. At the end of the assessment, participants selected their preferred days for training (Monday and Thursday or Tuesday and Friday) and provided an email address so that the research team could send links to the training sessions. Participants who completed the baseline assessment were randomized into 1 of 3 conditions (standard ABMT, gamified ABMT, or control) and invited by email to start their first training session. This email included a web link to the appropriate version of the training as well as instructions on how to download and install the software used to run the program.

Participants performed the training sessions on the web at their time and place of convenience, twice a week on a separate pair of days (Monday and Thursday or Tuesday and Friday) for 3 consecutive weeks, totaling 6 training sessions. This dosage was based on previous pain ABMT literature [10,12,16], which has shown positive training effects for dosage ranging between 4 and 8 sessions. After each training session, participants were asked to rate their engagement with the task. The first and final sessions took approximately 30 minutes, as they included cognitive assessment measures (ie, the dot-probe and interpretation bias assessment tasks were administered at the beginning, before training at session 1, and at the end, after the last training block in session 6), whereas sessions 2 to 5 took approximately 15 minutes to complete. Participants were asked to complete the sessions within 24 hours of receiving a web link on a computer with a proper keyboard and to create a quiet and private environment free from distractions for at least 30 minutes. Each session started with the same instructions, similar to those used in previous research (eg, [20]). A combination of SMS text message and email message reminders were sent to those who did not complete the scheduled session within 24 hours of receiving the web link. Participants were allowed to skip training sessions but were excluded from the analyses if they did not successfully complete at least 1 session (ie, minimum threshold for exposure to ABMT). They could contact the first author (JFV) by email or phone if they had any questions or technical problems.

Immediately after completion of the final training session, participants were automatically invited to the posttraining assessment (approximately 15 min), consisting of questions about pain treatments, a manipulation check, and the PROMIS Pain Intensity 3a, PROMIS Pain Interference 8a, PROMIS Anxiety 8a, PROMIS Depression 8b, ACS, IMI (interest and enjoyment), and PGIC. Participants were encouraged to complete the posttraining assessment regardless of whether they completed all training sessions. Finally, 1 month after the end of the last training session, all participants were sent a link to complete the follow-up assessment (approximately 10 min), which included the same questions as that of the posttraining assessment, except for the manipulation check, the ACS, and IMI (interest and enjoyment). Participants who failed to complete the follow-up assessment received up to 2 emails or SMS text message reminders.

### Data Preparation and Data Analysis

Statistical analyses were performed using SPSS (version 27.0; IBM Corp). Analyses included all randomized participants who successfully completed at least 1 training session. To manage missing data, linear mixed model analyses were used (where appropriate), as it allows the inclusion of all available data. Missing PROMIS measures data were handled according to the recommendations in the scoring manual, using the HealthMeasures Scoring Service [59]. Concomitant care received during the study was categorized into pain treatment received (*yes* or *no*) and use of medication (*yes* or *no*). Significance for all statistical tests were set at *P*<.05 (2-tailed). Effect sizes were presented by the test’s most appropriate effect size [79]. For the linear mixed models, a standardized Cohen *d* was calculated from the estimated marginal means tables [80]. No analyses were performed until recruitment and data collection were completed.

Participant characteristics were analyzed using descriptive statistics, and chi-square test, Fisher-Freeman-Halton exact test, 1-way ANOVA, and nonparametric Kruskal-Wallis test were used for group comparisons. To prepare reaction time (RT) data for analyses, practice trials, digit trials, incorrect trials, and outliers (ie, responses <200 or >1000 ms) were excluded from the calculation of mean RTs [20,36]. In addition, the data of 2 participants at pretraining assessment and 1 participant at posttraining assessment were excluded from the attentional bias analyses because they committed errors on >30% of the trials, showing suboptimal dot-probe task performance. Furthermore, the data of another 2 participants at pretraining assessment had to be discarded due to having a very high percentage of outliers (>96.8%). The mean percentage of dot-probe errors made by participants was 3.2%, and the mean percentage of outliers was 5.6%. An attentional bias index was calculated using the following formula: (tupl–tlpl)+(tlpu–tupu)/2, where t=target (pain) stimulus, p=probe, u=upper location, and l=lower location. Positive scores indicated an attentional bias toward pain-related stimuli, whereas negative scores reflected an attentional bias toward non–pain-related stimuli (or away from pain-related stimuli). To determine whether pain-related attentional biases were present at baseline, 1-sample 2-tailed *t* tests (vs 0) were performed on the pretraining attentional bias index scores for each condition separately.

A series of linear mixed model analyses were conducted to examine changes over time in symptoms (ie, pain intensity, pain interference, anxiety, and depression), self-reported task-related engagement, cognitive biases (ie, attentional bias and interpretation bias), and perceived improvement in the different training conditions. The categories baseline, control, married or in a relationship, employed, and session 1 were used as reference categories for time, training condition, marital status, work status, and session, respectively. A backward modeling approach was used to build the most parsimonious model to test the hypotheses [81], using the Akaike information criterion (AIC) and Bayesian information criterion (BIC) to identify the most appropriate model. Specifically, a 3-step model-building procedure was used to identify the best-fit model. First (model 1), we constructed a model that included the main effects and covariates. Second (model 2), we removed from the model covariates that were nonsignificant to see if it improved the fit of the model. Third (model 3), we added the interaction terms to the previously best-fit model (model 1 or model 2) and compared the 3 models on their fit to the data using the AIC and BIC. All models incorporated a random intercept for participants and used the maximum likelihood estimator. Finally, sensitivity analyses were conducted on all primary analyses without controlling for the covariates. Comparisons of models for each primary and secondary outcome variable are available in Multimedia Appendix 3.

To analyze the impact of gamification on interest and enjoyment, a 1-way analysis of covariance in a general linear model was performed (no data were missing). Regarding behavioral engagement, Kaplan-Meier survival curves [82] were calculated to assess the time at which attrition occurred in each training condition and compared statistically using a log-rank test. The number of sessions completed was the time variable, and the event variable was specified as the moment of ceasing participation. Participants were classified as noncompleters if they did not complete all 6 training sessions. In addition, a 1-way analysis of covariance in a general linear model was performed to determine whether there were differences in the mean number of sessions completed between the training conditions. Pearson correlations assessed the relationship between changes in attentional bias magnitude from pretraining to posttraining assessment and changes in scores on symptom measures (ie, pain intensity, pain interference, anxiety, and depression). Change in attentional bias was calculated by subtracting the attentional bias score in the pretraining session from the attentional bias score in the posttraining session.

Finally, exploratory analyses were conducted to address additional questions. These included the role of engagement metrics (ie, number of training sessions completed) and individual differences (ie, attentional control, pain-related worrying, personality characteristics, and recruitment setting) in the impact of training conditions on pain intensity and pain interference. Models for each exploratory analysis are available in Multimedia Appendix 2.

## Results

### Participant Flow

**Figure 2:**
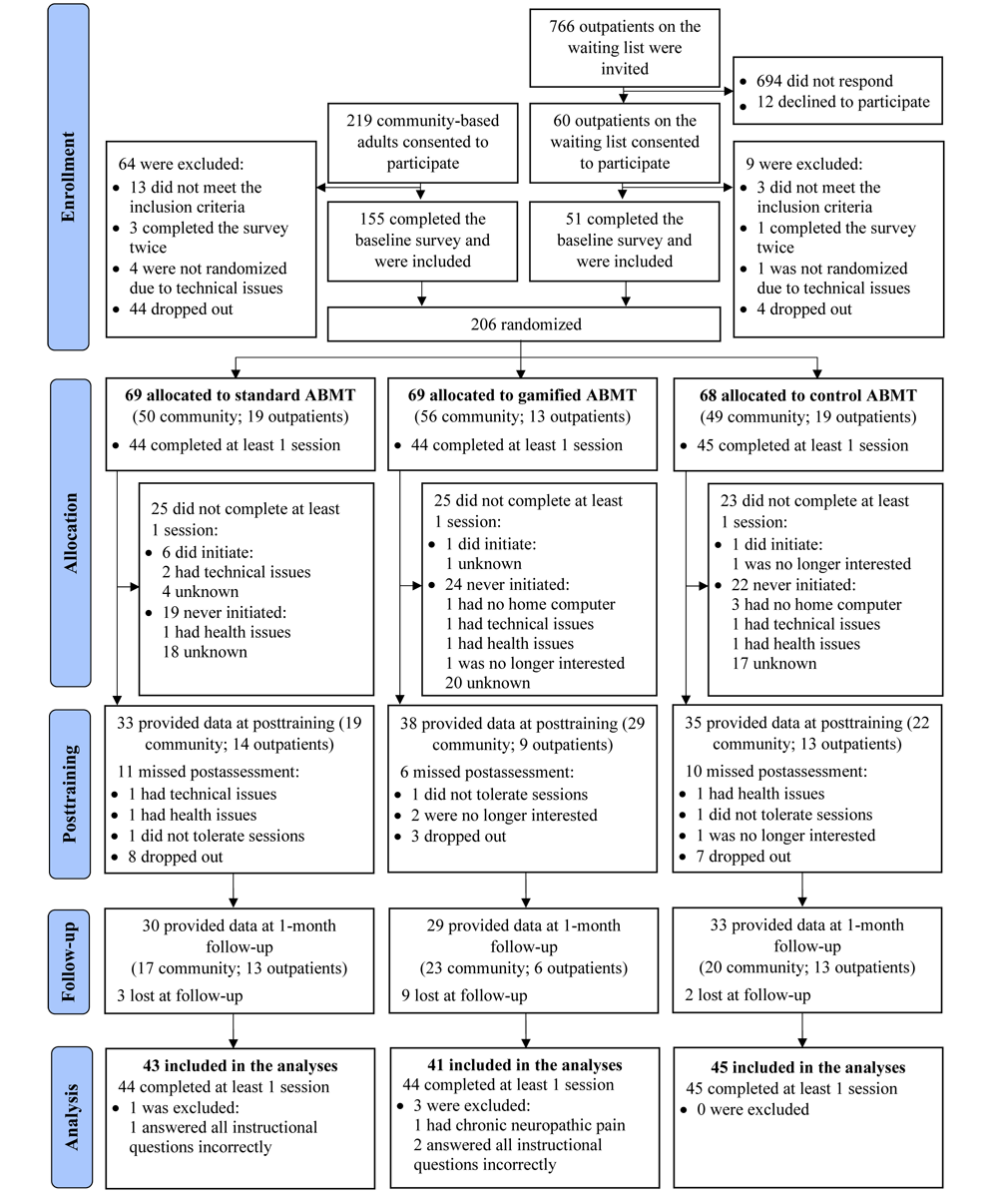
CONSORT (Consolidated Standards of Reporting Trials) diagram of flow of participants through the study. ABMT: attentional bias modification training.

The first participant for this study was enrolled in August 2021, and the final follow-up assessment was completed in June 2022. Figure 2 shows the CONSORT (Consolidated Standards of Reporting Trials) flow diagram for the study. Of the 766 outpatients on the waiting list that were invited to participate, 60 (7.8% response rate) consented to participate, and of those 60, 51 (85%) were included and randomized to conditions. Of the 219 community-based adults that consented to participate, 155 (70.8%) were included and randomized to conditions. In total, 206 participants were randomized into the standard ABMT (n=69, 33.5%), gamified ABMT (n=69, 33.5%), or control condition (n=68, 33%). Of these 206 participants, 106 (51.5%) completed the posttraining survey (n=33, 16% standard ABMT; n=38, 18.5% gamified ABMT; and n=35, 17% control) and 92 (44.7%) completed the 1-month follow-up survey (n=30, 14.6% standard ABMT; n=29, 14.1% gamified ABMT; and n=33, 16% control). Of the 206 participants who were randomized, 73 (35.4%) were excluded from all analyses due to not successfully completing at least 1 training session, which was the minimum threshold for exposure to ABMT (n=25, 12.1% standard ABMT; n=25, 12.1% gamified ABMT; and n=23, 11.2% control), 3 (1.5%) were excluded for answering all the instructional questions incorrectly (n=1, 0.5% standard ABMT and n=2, 1% gamified ABMT), and 1 (0.5%) was excluded for having only chronic neuropathic pain (gamified ABMT). The final sample size included in the analysis was 129 adults with chronic musculoskeletal pain (n=43, 33.3% standard ABMT; n=41, 31.8% gamified ABMT; and n=45, 34.9% control).

### Sample Characteristics and Baseline Group Differences

Descriptive statistics for baseline key characteristics and outcome measures are presented in Tables 2 and 3, respectively. The mean age of the 129 participants was 49.49 (SD 12.50) years. The participants were primarily female, born in Australia, tertiary educated (ie, university, college, or posthigh school qualifications), married or in a relationship, employed, and right-handed. Most participants (59/129, 45.7%) reported that their general health was *fair,* but that they had mental health problems (eg, anxiety). Nearly all participants (124/129, 96.1%) in the sample had received a diagnosis for their pain. Chronic lower back pain was the most common pain problem. The mean duration of pain was 13.56 (SD 10.58) years. On the GCPS, participants’ mean current pain was 5.81 (SD 1.94), and calculation of the pain grade showed that most participants (69/129, 53.5%) were classified in grade 4 (*high disability*-*high intensity*). Most participants had consulted a physician for their pain in the past 4 weeks, were taking regular medication (ie, nonprescription and prescription medicines) to alleviate their pain, and were receiving pain treatment (eg, physical interventions).

**Table 2:** Baseline key characteristics of the sample by training condition (N=129).

Variable		Full sample	By training condition				*P* value	
			Standard ABMT^a^ (n=43)	Gamified ABMT (n=41)	Control (n=45)			
**Key demographics**								
	Age (y), mean (SD)	49.49 (12.50)	48.09 (12.51)	47.78 (11.88)	52.38 (12.79)	.16		
	Gender (female), n (%)	104 (80.6)	35 (81.4)	36 (87.8)	33 (73.3)	.44		
	Country of birth (Australia), n (%)	105 (81.4)	34 (79.1)	32 (78)	39 (86.7)	.53		
	Education level (tertiary), n (%)	101 (78.3)	37 (86)	37 (90.2)	27 (60)	.003^b^		
	Relationship status (married or in a relationship), n (%)	75 (58.1)	28 (65.1)	16 (39)	31 (68.9)	.01^b^		
	Employment status (employed), n (%)	55 (42.6)	18 (41.9)	27 (65.9)	10 (22.2)	<.001^b^		
	Handedness (right-handed), n (%)	114 (88.4)	39 (90.7)	37 (90.2)	38 (84.4)	.88		
**General health status, n (%)**								.45
	Very good	3 (2.3)	1 (2.3)	1 (2.4)	1 (2.2)			
	Good	38 (29.5)	10 (23.3)	15 (36.6)	13 (28.9)			
	Fair	59 (45.7)	18 (41.9)	18 (43.9)	23 (51.1)			
	Bad	23 (17.8)	11 (25.5)	7 (17.1)	5 (11.1)			
	Very bad	6 (4.7)	3 (7)	0 (0)	3 (6.7)			
Mental health condition (yes), n (%)		71 (55)	26 (60.5)	20 (48.8)	25 (55.6)	.56		
Diagnosis (yes), n (%)		124 (96.1)	41 (95.3)	38 (92.7)	45 (100)	.16		
Type of pain (chronic lower back pain), n (%)		92 (71.3)	34 (79.1)	25 (61)	33 (73.3)	.19		
Primary pain site (lower back), n (%)		61 (47.3)	22 (51.2)	16 (39)	23 (51.1)	.78		
Duration of chronic pain^c^ (y), mean (SD)		13.56 (10.58)	14.02 (9.87)	12.91 (10.48)	13.70 (11.50)	.41		
GCPS^d^ pain intensity, mean (SD)		5.81 (1.94)	6.14 (1.77)	5.37 (2.13)	5.89 (1.87)	.18		
**GCPS scales, n (%)**								.60
	Grade 0: no pain problem	0 (0)	0 (0)	0 (0)	0 (0)			
	Grade 1: low disability-low intensity	8 (6.2)	4 (9.3)	2 (4.9)	2 (4.4)			
	Grade 2: low disability-high intensity	20 (15.5)	4 (9.3)	10 (24.4)	6 (13.3)			
	Grade 3: high disability-low intensity	32 (24.8)	11 (25.6)	10 (24.4)	11 (24.4)			
	Grade 4: high disability-high intensity	69 (53.5)	24 (55.8)	19 (46.3)	26 (57.8)			
Medical visit in the past 4 week (yes), n (%)		95 (73.6)	33 (76.7)	28 (68.3)	34 (75.6)	.64		
Use of medication (yes), n (%)		126 (97.7)	41 (95.3)	41 (100)	44 (97.8)	.65		
Pain treatment (yes), n (%)		114 (88.4)	38 (88.4)	38 (92.7)	38 (84.4)	.55		
Computer type (laptop), n (%)		79 (61.2)	28 (65.1)	23 (56.1)	28 (62.2)	.65		
Computer screen (≤50.8 cm), n (%)		64 (49.6)	20 (46.5)	18 (43.9)	26 (57.8)	.16		

^a^ABMT: attentional bias modification training.

^b^Statistical significance: *P*<.05, 2-tailed.

^c^One missing value in the gamified ABMT condition.

^d^GCPS: Graded Chronic Pain Scale.

**Table 3:** Summary statistics on outcome measures by training condition and assessment time points.

Variable						Training condition																			*P* value^a^
						Standard ABMT^b^ (n=43)						Gamified ABMT (n=41)						Control (n=45)							
						Values, n (%)			Values, mean (SD)			Values, n (%)			Values, mean (SD)			Values, n (%)			Values, mean (SD)				
**Primary outcomes**																									
	**Task-related engagement**																								
		Session 1			37 (86)			5.81 (3.10)			38 (93)			6.03 (2.95)			43 (96)			6.14 (2.74)			—^c^		
		Session 2			34 (79)			4.79 (3.06)			38 (93)			5.79 (2.92)			38 (84)			5.95 (3.11)			—		
		Session 3			29 (67)			4.83 (3.34)			35 (85)			5.49 (2.96)			36 (80)			5.47 (3.05)			—		
		Session 4			28 (65)			5.14 (3.34)			30 (73)			5.00 (2.91)			34 (76)			5.59 (2.89)			—		
		Session 5			27 (63)			4.96 (3.35)			28 (68)			4.46 (2.85)			30 (67)			5.90 (2.77)			—		
		Session 6			26 (61)			4.81 (3.18)			31 (76)			4.23 (3.01)			31 (69)			5.32 (3.05)			—		
	Number of sessions completed					43 (100)			4.21 (2.01)			41 (100)			4.88 (1.62)			45 (100)			4.71 (1.80)			—	
	IMI^d^					32 (74)			3.45 (1.50)			35 (85)			2.89 (1.59)			35 (78)			3.14 (1.21)			—	
	**PROMIS^e^ pain intensity**																								
		Baseline			43 (100)			66.06 (7.23)			41 (100)			65.51 (7.00)			45 (100)			65.86 (6.40)			.94		
		Posttraining			32 (74)			67.23 (8.59)			35 (85)			63.25 (7.28)			35 (78)			65.34 (6.66)			—		
		Follow-up			29 (67)			65.33 (8.64)			28 (68)			61.31 (6.48)			33 (73)			64.04 (8.93)			—		
	**PROMIS pain interference**																								
		Baseline			43 (100)			66.00 (7.53)			41 (100)			63.73 (6.23)			45 (100)			66.88 (6.42)			.09		
		Posttraining			32 (74)			64.77 (7.40)			35 (85)			62.85 (7.52)			35 (78)			66.01 (6.72)			—		
		Follow-up			29 (67)			64.05 (7.64)			28 (68)			62.70 (6.28)			33 (73)			62.85 (8.50)			—		
**Secondary outcomes**																									
	**Attentional bias index**																								
		Baseline			40 (93)			–0.58 (18.61)			41 (100)			–3.72 (24.41)			44 (98)			1.30 (20.23)			.55		
		Posttraining			26 (61)			2.12 (15.08)			30 (73)			3.98 (16.90)			31 (69)			–1.65 (20.52)			—		
	**PROMIS anxiety**																								
		Baseline			43 (100)			59.97 (11.39)			41 (100)			57.86 (9.05)			45 (100)			61.24 (8.03)			.26		
		Posttraining			32 (74)			61.00 (12.11)			35 (85)			58.78 (9.47)			35 (78)			59.15 (8.33)			—		
		Follow-up			29 (67)			59.55 (10.13)			28 (68)			56.80 (9.71)			33 (73)			59.16 (8.49)			—		
	**PROMIS depression**																								
		Baseline			43 (100)			61.28 (11.05)			41 (100)			56.97 (10.09)			45 (100)			61.92 (8.07)			.04^g^		
		Posttraining			32 (74)			61.28 (11.17)			35 (85)			55.68 (10.40)			35 (78)			58.64 (8.53)			—		
		Follow-up			29 (67)			61.20 (9.39)			28 (68)			55.20 (10.19)			33 (73)			60.05 (10.58)			—		
	**PGIC^f^**																								
		**Posttraining**																							
			Illness improvement	6 (14)			—			6 (15)			—			4 (9)			—			—			
			Stable illness	20 (47)			—			23 (56)			—			29 (64)			—			—			
			Illness deterioration	6 (14)			—			6 (15)			—			2 (4)			—			—			
		**Follow-up**																							
			Disease improvement	7 (16)			—			6 (15)			—			11 (24)			—			—			
			Stable disease	17 (40)			—			18 (44)			—			16 (36)			—			—			
			Disease deterioration	5 (12)			—			4 (10)			—			6 (13)			—			—			
**Interpretation bias**																									
	**Baseline**																								
		Belief: health and pain			43 (100)			0.41 (1.12)			41 (100)			–0.12 (0.93)			45 (100)			0.04 (0.94)			.07		
		Belief: social			43 (100)			–0.66 (1.44)			41 (100)			–1.30 (1.15)			45 (100)			–0.94 (1.07)			.06		
	**Posttraining**																								
		Belief: health and pain			27 (63)			0.51 (0.96)			30 (73)			–0.16 (0.82)			31 (69)			–0.08 (0.77)			—		
		Belief: social			27 (63)			–0.66 (1.29)			30 (73)			–0.98 (1.11)			31 (69)			–0.92 (0.86)			—		

^a^*P* values calculated for baseline differences only.

^b^ABMT: attentional bias modification training.

^c^Not applicable as differences between groups are examined with linear mixed model analyses and are reported in Tables 4 and 5.

^d^IMI: Intrinsic Motivation Inventory, subscales interest and enjoyment.

^e^PROMIS: Patient-Reported Outcomes Measurement Information System.

^f^PGIC: Patient Global Impression of Change.

^g^Statistical significance *P*<.05, 2-tailed

A series of analyses were conducted to determine whether there were any baseline differences between the ABMT conditions on key characteristics (Table 2) and outcome measures (Table 3). The training conditions did differ significantly at baseline on marital status, educational level, employment status, and depressive symptomology. Specifically, the participants in the gamified ABMT condition reported significantly lower scores for depression (*F*_2,126_=3.19; *P*=.04) and were significantly less likely to be married or in a relationship (*χ^2^*_4_=12.7; *P*=.01). Furthermore, the participants in the control condition were significantly less likely to have a tertiary education (Fisher-Freeman-Halton exact test=13.43; *P*=.003) and to be working (Fisher-Freeman-Halton exact test=23.01; *P*<.001). The baseline covariates were accounted for in the analyses as per the statistical plan.

### Primary Outcomes

#### Engagement

Table 4 displays the results of the linear mixed model for self-reported task-related engagement. There was a significant main effect of session between sessions 1 and 2 (*P*<.001), sessions 1 and 3 (*P*<.001), sessions 1 and 4 (*P*<.001), sessions 1 and 5 (*P*<.001), and sessions 1 and 6 (*P*<.001), with small to medium effect sizes, reflecting an overall decrease in task-related engagement ratings after session 1 over the 6 sessions (session 1: mean 6.00, SD 2.90; session 2: mean 5.54, SD 3.04; session 3: mean 5.29, SD 3.09; session 4: mean 5.26, SD 3.02; session 5: mean 5.13, SD 3.02; and session 6: mean 4.78, SD 3.07). However, there was no significant main effect of training condition (*P* values>.05; AIC=2384.19; BIC=2449.96). The model that included the interaction effects (Table S1 in Multimedia Appendix 3) did not fit the data better than the previous model, and all interactions were nonsignificant for session×condition (*P* values>.05; AIC=2396.60; BIC=2506.23). The 1-way analysis of covariance revealed that there was no significant difference in the mean interest and enjoyment (IMI) scores between training conditions (*F*_2,91_=0.94; *P*=.40; η^2^_p_=0.020).

**Figure 3:**
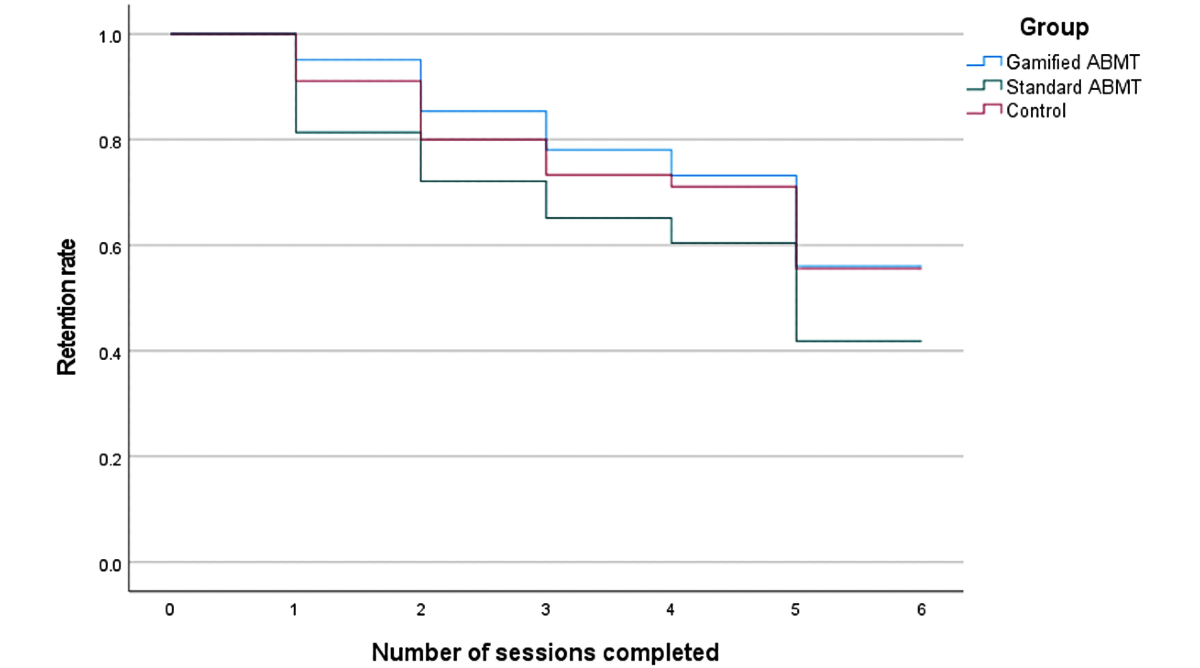
Kaplan-Meier survival curves for dropout by training condition over the 3-week intervention period. ABMT: attentional bias modification training.

On average, participants completed 4.60 (SD 1.83) out of 6 training sessions, which did not differ significantly between conditions (*F*_2,118_=1.24; *P*=.29; η^2^_p_=0.021). The attrition pattern of the 3-week intervention period is presented in the Kaplan-Meier plot in Figure 3. Nonuse intervention attrition occurred for 58% (25/43), 44% (18/41), and 44% (20/45) of the standard ABMT, gamified ABMT, and control conditions, respectively, which is comparable to other web-based health interventions [83-86]. Dropout occurred throughout the course of the intervention, with no significant differences between conditions in the time of attrition (log-rank *χ*_2_^2^=2.8; *P*=.25). For those who provided unsolicited feedback (25/63, 40%), the reasons for nonuse intervention attrition were technical issues (8/25, 32%), sick or in hospital (5/25, 20%), unable to tolerate the training sessions (4/25, 16%), lack of interest (4/25, 16%), too busy or away (2/25, 8%), and no access to a computer due to the COVID-19 pandemic lockdown or floods (2/25, 8%).

**Table 4:** Linear mixed models for primary outcomes.

Variable			Β (SE; 95% CI)		*P* value		Cohen *d* (95% CI)
**Engagement**							
	Intercept	5.72 (0.59; 4.55 to 6.90)		<.001^a^		—^b^	
	Session 2	–0.69 (0.18; –1.03 to –0.34)		<.001^a^		0.257 (0.13 to 0.39)	
	Session 3	–0.99 (0.18; –1.35 to –0.63)		<.001^a^		0.368 (0.23 to 0.50)	
	Session 4	–1.28 (0.19; –1.65 to –0.91)		<.001^a^		0.473 (0.34 to 0.61)	
	Session 5	–1.33 (0.19; –1.71 to –0.96)		<.001^a^		0.488 (0.35 to 0.63)	
	Session 6	–1.63 (0.19; –2.00 to –1.25)		<.001^a^		0.596 (0.46 to 0.73)	
	Gamified ABMT^c^	0.55 (0.67; –0.77 to 1.87)		.41		–0.040 (–0.14 to 0.06)	
	Standard ABMT	–0.20 (0.60; –1.38 to 0.98)		.74		0.017 (–0.08 to 0.12)	
	Single (covariate)	–1.54 (0.56; –2.64 to –0.43)		.007^a^		—	
	Divorced or separated (covariate)	0.22 (0.75; –1.26 to 1.69)		.77		—	
	Student (covariate)	1.09 (0.87; –0.64 to 2.81)		.22		—	
	Retired (covariate)	1.71 (0.67; 0.37 to 3.04)		.01^a^		—	
	Not employed (covariate)	0.67 (0.63; –0.58 to 1.92)		.29		—	
**Pain intensity**							
	Intercept	51.17 (3.50; 44.24 to 58.10)		<.001^a^		—	
	Posttraining	–0.36 (0.68; –1.70 to 0.99)		.60		0.034 (–0.10 to 0.16)	
	Follow-up	–1.90 (0.71; –3.31 to –0.50)		.008^a^		0.180 (0.05 to 0.31)	
	Gamified ABMT	–0.48 (1.31; –3.06 to 2.11)		.72		0.025 (–0.11 to 0.16)	
	Standard ABMT	1.01 (1.27; –1.50 to 3.52)		.43		–0.054 (–0.19 to 0.08)	
	Depression (covariate)	0.24 (0.06; 0.13 to 0.35)		<.001^a^		—	
**Pain interference**							
	Intercept	43.53 (2.89; 37.83 to 49.24)		<.001^a^		—	
	Posttraining	–0.88 (0.58; –2.02 to 0.26)		.13		0.100 (–0.03 to 0.23)	
	Follow-up	–2.12 (0.61; –3.31 to –0.93)		<.001^a^		0.237 (0.10 to 0.37)	
	Gamified ABMT	1.63 (1.19; –0.72 to 3.99)		.17		–0.093 (–0.23 to 0.04)	
	Standard ABMT	0.27 (1.06; –1.84 to 2.37)		.80		–0.017 (–0.15 to 0.12)	
	Single (covariate)	–3.32 (1.02; –5.34 to –1.30)		.001^a^		—	
	Divorced or separated (covariate)	–1.11 (1.32; –3.72 to 1.51)		.41		—	
	Student (covariate)	0.77 (1.56; –2.32 to 3.86)		.62		—	
	Retired (covariate)	4.40 (1.21; 2.01 to 6.78)		<.001^a^		—	
	Not employed (covariate)	3.94 (1.16; 1.65 to 6.23)		<.001^a^		—	
	Depression (covariate)	0.34 (0.05; 0.25 to 0.43)		<.001^a^		—	

^a^Statistical significance: *P*<.05, 2-tailed.

^b^Not applicable.

^c^ABMT: attentional bias modification training.

#### Pain Intensity

Table 4 displays the results of the linear mixed model for PROMIS Pain Intensity. There was a significant main effect of time from baseline to follow-up assessment (*P*=.008), with small effect size, indicating an overall small decrease in pain intensity scores over time across all conditions (baseline: mean 65.81, SD 6.83; follow-up: mean 63.61, SD 8.22). However, there was no significant main effect of time from baseline to posttraining assessment (*P*=.60) or of training condition (*P* values>.05; AIC=2113.76; BIC=2143.93). The model that included the interaction effects (Table S2 in Multimedia Appendix 3) did not fit the data better than the previous model, and all interactions were nonsignificant for time×condition (*P* values>.05; AIC=2115.34; BIC=2160.60).

#### Pain Interference

Table 4 displays the results of the linear mixed model for PROMIS Pain Interference scores. There was a significant main effect of time from baseline to follow-up assessment (*P<*001), with small effect size, indicating an overall small decrease in pain interference scores over time across all conditions (baseline: mean 65.58, SD 6.83; follow-up: mean 63.19, SD 7.53). However, there was no significant main effect of time from baseline to posttraining assessment (*P*=.13) or of training condition (*P* values>.05; AIC=2006.30; BIC=2055.33). The model that included the interaction effects (Table S3 in Multimedia Appendix 3) did not fit the data better than the previous model. The model, however, showed a significant interaction for follow-up×gamified (*P*=.03; AIC=2007.61; BIC=2071.72). All the other interactions were nonsignificant (*P* values>.05).

### Secondary Outcomes

#### Measure of Attentional Bias for Pain

The 1-sample *t* tests against 0 indicated that there were no significant attentional biases toward pain-related stimuli observed at baseline in the standard ABMT (mean –0.58, SD 18.61; *t*_39_=–0.20; *P=*.84; Cohen *d*=–0.031; 95% CI –0.34 to 0.28), gamified ABMT (mean –3.72, SD 24.41; *t*_40_=–0.98; *P=*.34; Cohen *d*=–0.152; 95% CI –0.46 to 0.16), or control (mean 1.30, SD 20.23; *t*_43_=0.43; *P=*.67; Cohen *d*=0.064; 95% CI –0.23 to 0.36) conditions. Table 5 displays the results of the linear mixed model for attentional bias index. There was no significant main effect of time on attentional bias index scores from baseline to posttraining assessment (*P*=.39) or of training condition (*P* values>.05; AIC=1878.14; BIC=1898.28). The model that included the interaction effects (Table S4 in Multimedia Appendix 3) did not fit the data better than the previous model, and all interactions were nonsignificant for time×condition (*P* values>.05; AIC=1879.54; BIC=1906.39). Correlations between changes in attentional bias magnitude and changes on pain intensity, pain interference, anxiety, and depression measures from baseline to posttraining assessment were all nonsignificant (*P* values>.05; Table S5 in Multimedia Appendix 3).

**Table 5:** Linear mixed models for secondary outcomes.

Variable		Β (SE; 95% CI)	*P* value	Cohen *d* (95% CI)
**Attentional bias index**				
	Intercept	–0.91 (2.55; –5.93 to 4.12)	.72	—^a^
	Posttraining	2.39 (2.76; –3.05 to 7.82)	.39	–0.059 (–0.19 to 0.08)
	Gamified ABMT^b^	–0.57 (3.27; –7.01 to 5.88)	.86	0.014 (–0.15 to 0.18)
	Standard ABMT	0.45 (3.33; –6.12 to 7.02)	.89	–0.011 (–0.18 to 0.15)
**Anxiety**				
	Intercept	16.40 (3.45; 9.58 to 23.22)	<.001^c^	—
	Posttraining	–0.43 (0.65; –1.71 to 0.86)	.51	0.043 (–0.09 to 0.17)
	Follow-up	–1.16 (0.68; –2.51 to 0.18)	.09	0.116 (–0.02 to 0.25)
	Gamified ABMT	1.46 (1.29; –1.09 to 4.00)	.26	–0.077 (–0.21 to 0.06)
	Standard ABMT	–0.06 (1.25; –2.54 to 2.42)	.96	0.003 (–0.13 to 0.14)
	Depression (covariate)	0.71 (0.05; 0.61 to 0.82)	<.001^c^	—
**Depression**				
	Intercept	54.65 (1.94; 50.81 to 58.49)	<.001^c^	—
	Posttraining	–1.82 (0.66; –3.12 to –0.51)	.007^c^	0.180 (0.05 to 0.31)
	Follow-up	–1.17 (0.70; –2.54 to 0.20)	.09	0.114 (–0.02 to 0.25)
	Gamified ABMT	–0.90 (2.10; –5.06 to 3.26)	.67	0.029 (–0.11 to 0.16)
	Standard ABMT	2.07 (1.90; –1.68 to 5.82)	.28	–0.074 (–0.21 to 0.06)
	Primary (covariate)	–8.46 (8.39; –25.08 to 8.16)	.32	—
	Secondary (covariate)	5.04 (1.92; 1.24 to 8.85)	.01^c^	—
	Single (covariate)	3.94 (1.73; 0.51 to 7.37)	.03^c^	—
	Divorced or separated (covariate)	–0.78 (2.31; –5.36 to 3.79)	.74	—
	Student (covariate)	5.89 (2.69; 0.57 to 11.22)	.03^c^	—
	Retired (covariate)	3.14 (2.10; –1.02 to 7.31)	.14	—
	Not employed (covariate)	7.09 (1.95; 3.22 to 10.95)	<.001^c^	—
**Perceived improvement**				
	Intercept	3.76 (0.18; 3.40 to 4.12)	<.001^c^	—
	Follow-up	–0.03 (0.09; –0.22 to 0.15)	.73	0.025 (–0.12 to 0.17)
	Gamified ABMT	0.20 (0.19; –0.17 to 0.57)	.28	–0.095 (–0.27 to 0.08)
	Standard ABMT	0.11 (0.18; –0.24 to 0.46)	.53	–0.056 (–0.23 to 0.12)
	Primary (covariate)	–1.94 (0.69; –3.30 to –0.58)	.006^c^	—
	Secondary (covariate)	0.11 (0.17; –0.24 to 0.45)	.54	—
	Student (covariate)	–0.04 (0.24; –0.52 to 0.45)	.88	—
	Retired (covariate)	0.20 (0.20; –0.19 to 0.58)	.32	—
	Not employed (covariate)	0.42 (0.18; 0.07 to 0.77)	.02^c^	—
**Interpretation bias: pain and health belief**				
	Intercept	–0.96 (0.49; –1.93 to 0.02)	.06	—
	Posttraining	–0.04 (0.09; –0.21 to 0.14)	.68	0.028 (–0.11 to 0.16)
	Gamified ABMT	–0.08 (0.19; –0.45 to 0.29)	.67	0.036 (–0.13 to 0.20)
	Standard ABMT	0.42 (0.18; 0.07 to 0.78)	.02^c^	–0.195 (–0.36 to –0.31)
	Depression (covariate)	0.02 (0.01; 0.00 to 0.03)	.04^c^	—
**Interpretation bias: social belief**				
	Intercept	–2.62 (0.64; –3.88 to –1.36)	<.001^c^	—
	Posttraining	0.05 (0.09; –0.12 to 0.23)	.56	–0.040 (–0.18 to 0.09)
	Gamified ABMT	–0.27 (0.24; –0.74 to 0.20)	.26	0.093 (–0.07 to 0.26)
	Standard ABMT	0.31 (0.23; –0.15 to 0.77)	.18	–0.112 (–0.28 to 0.05)
	Depression (covariate)	0.03 (0.01; 0.01 to 0.05)	.007^c^	—

^a^ Not applicable

^b^ABMT: attentional bias modification training.

^c^Statistical significance: *P*<.05, 2-tailed.

#### Anxiety and Depression

Table 5 displays the results of the linear mixed model for PROMIS Anxiety and PROMIS Depression. There was no significant main effect of time on anxiety scores from baseline to posttraining assessment (*P*=.51) or from baseline to follow-up assessment (*P*=.09). Moreover, there was no significant main effect of training condition (*P* values>.05; AIC=2091.03; BIC=2121.20). The model that included the interaction effects (Table S6 in Multimedia Appendix 3) did not fit the data better than the previous model, and all interactions were nonsignificant for time×condition (*P* values>.05; AIC=2095.35; BIC=2140.60).

Regarding depressive symptomatology, there was a significant main effect of time on depression scores from baseline (mean 60.13, SD 9.95) to posttraining assessment (mean 58.45, SD 10.21; *P*=.007), with small effect size, indicating an overall small decrease in depressive symptoms immediately after the intervention across all training conditions. However, there was no significant main effect of time from baseline to follow-up assessment (*P*=.09) or of training condition (*P* values>.05; AIC=2196.33; BIC=2249.13). The model that included the interaction effects (Table S7 in Multimedia Appendix 3) did not fit the data better than the previous model, and all interactions were nonsignificant for time×condition (*P* values>.05; AIC=2201.58; BIC=2269.47).

#### Perceived Improvement

Table 3 presents the statistics of the PGIC after training and at 1-month follow-up. At posttraining assessment, 14% (6/43), 15% (6/41), and 9% (4/45) of participants in the standard ABMT, gamified ABMT, and control conditions, respectively, reported that their symptoms improved. At the 1-month follow-up assessment, 16% (7/43), 15% (6/41), and 24% (11/45) of participants in the standard ABMT, gamified ABMT, and control conditions, respectively, reported that their symptoms improved. As shown in Table 5, there was no significant main effect of time on perceived overall pain-related improvement scores from posttraining to follow-up assessment (*P*=.73) or of training condition (*P* values>.05; AIC=468.12; BIC=503.95). The model that included the interaction effects (Table S8 in Multimedia Appendix 3) did not fit the data better than the previous model, and all interactions were nonsignificant for time×condition (*P* values>.05; AIC=471.87; BIC=514.22).

#### Measure of Interpretation Bias for Pain

Table 5 displays the results of the linear mixed model for interpretation biases for pain. There was no significant main effect of time on interpretation bias scores for pain and bodily threat situations from baseline to posttraining assessment (*P*=.68). However, there was a significant main effect of training condition (*P*=.02), with small effect size, such that participants in the standard ABMT condition (mean 0.45, SD 1.06) endorsed more negative interpretations for scenarios related to pain and bodily threat compared to those in the control condition (mean –0.01, SD 0.87). There was no significant difference in interpretation bias scores for pain and bodily threat between the gamified ABMT and control conditions (*P*=.67; AIC=558.34; BIC=582.00). The model that included the interaction effects (Table S9 in Multimedia Appendix 3) did not fit the data better than the previous model, and all interactions were nonsignificant for time×condition (*P* values>.05; AIC=561.97; BIC=592.39).

Regarding social interpretation, there was no significant main effect of time on interpretation bias scores for social situations from baseline to posttraining assessment (*P*=.56) or of training condition (*P* values>.05; AIC=621.84; BIC=645.50). The model that included the interaction effects (Table S10 in Multimedia Appendix 3) did not fit the data better than the previous model, and all interactions were nonsignificant for time×condition (*P* values>.05; AIC=625.11; BIC=655.53).

### Additional and Exploratory Analyses

#### Exploratory Analyses

Multimedia Appendix 2 displays the results of the additional exploratory analyses. Overall, none of these variables were found to moderate the effects of ABMT on pain outcomes, except for Behavioral Activation System on pain interference (refer to Multimedia Appendix 2 for further details).

#### Manipulation Check

At the end of the posttraining assessment, participants were asked which treatment they believed they had received (ie, intervention or sham training). Overall, 41.2% (42/102) of the participants who responded to the question identified their allocated treatment condition correctly. The proportion of participants who thought they were in the treatment condition was low, with 22% (7/32) for the standard ABMT, 20% (7/35) for the gamified ABMT, and 20% (7/35) for the control condition, a difference that was not statistically significant (*χ^2^*_2_=0.1; *P*=.99).

### Sensitivity Analyses

Sensitivity analyses were conducted on all primary analyses without controlling for marital status, educational level, employment status, and baseline depressive symptomology as covariates. The pattern of results remained unchanged for pain intensity, pain interference, and engagement (Tables S11-S14 in Multimedia Appendix 3).

### Adverse Events and Negative Experiences

Similar to previous studies (eg, [20]), no serious adverse events were reported. However, 5 participants (3/41, 7% gamified ABMT; 1/43, 2% standard ABMT; and 1/45, 2% control) discontinued their participation due to training-related nonserious adverse events. Specifically, despite being offered a rest between each block of trials, 4 participants reported that the physical action required to complete the tasks (ie, repetitive movements and sitting for prolonged periods) triggered pain in their hand, wrist, back, or neck, and 1 participant reported that the task caused dizziness. Of these 5 participants, 2 (2/129, 1.6%) specified that their difficulty in performing the tasks was aggravated by the COVID-19 pandemic lockdowns, which prevented them from receiving regular treatment for their pain. Another participant in the standard ABMT condition discontinued participation due to fear that the thin black writing on a white background might be a migraine trigger and suggested offering the option of a dark mode (ie, white or gray text presented against a dark or back background) and large text (ie, bolding and increasing the font size).

## Discussion

### Principal Findings

This study investigated the effects of a gamified web-delivered pain ABMT intervention in adults with chronic musculoskeletal pain. The findings can be readily summarized. First, there was no evidence that gamified ABMT was effective in enhancing engagement over and above the standard ABMT and sham training control conditions. Second, there were also no differential effects over time between the active ABMT and control conditions on measures of pain intensity, pain interference, depression, anxiety, or perceived improvement. Third, depressive symptoms, pain intensity, and pain interference reduced over time across all conditions. Finally, there was no indication for the presence of pretraining attentional biases in any of the conditions and no evidence that cognitive biases changed after training for the active ABMT conditions compared to the control condition.

### Comparison With Previous Work

This study extends previous pain ABMT research [10,12,16-20] by examining the effects of gamification of a web-delivered pain ABMT intervention in adults with chronic musculoskeletal pain. Unexpectedly, there was no indication that participants in the gamified version of the ABMT task rated higher on self-reported task-related engagement, interest, and enjoyment or that they completed more training sessions or had less dropout compared to those in the nongamified versions. These results are inconsistent with the findings of several reviews [25,26] and recent studies [27,87-89], which found evidence for the potential of gamification to enhance motivation, engagement, and enjoyment in cognitive tasks. In fact, self-reported task-related engagement ratings were only moderate even at the first session (mean 6.00, SD 2.90; rating: 0-11), and all conditions on average reported relatively low levels of interest and enjoyment (IMI measure) immediately after the training (mean 3.15, SD 1.44; rating: 1-7). There was also an overall decline in self-reported task-related engagement over the 6 sessions. This is in line with the findings found in the study by Boendermaker et al [90], who found in their gamified ABMT for alcohol study that motivation to train decreased over time across both gamified and nongamified conditions. Thus, it seems that adding minimal game elements alone is not enough to counteract the monotony of the dot-probe task, which is of ongoing concern [21-23].

The finding that there was no superiority of the gamified ABMT condition over the other conditions was also unexpected but is in accordance with the results of a recent meta-analysis, which found no effect of gamification on cognitive or clinical outcomes [25]. Together, these findings do not imply that gamification cannot have positive effects on engagement and other outcomes, but rather highlight the complexity of designing effective gamified interventions. The development of the gamified ABMT task followed the Medical Research Council framework for complex interventions [38], using theory (ie, self-determination theory and self-regulation), reviewing evidence, and expert involvement. Despite this, it is possible that the type and combination of game elements we selected were not optimal. To date, it is still unclear as to which and how many game elements should be combined to optimize motivation [91,92]. There might also have been a ceiling effect (ie, participants completed, on average, 4.60, SD 1.83 out of 6 training sessions) where all participants were already, at baseline, highly motivated to complete the treatment course, making it difficult for the gamification to have improved further on this already high motivation to engage with the task. In accordance with the preregistered protocol, we excluded participants who did not complete at least 1 training session. As shown in Figure 2, a small minority of these excluded participants (8/73, 11%) had initiated a session, and it is possible that they did not find it engaging, leading to their decision to drop out before completing a session. As such, engagement could be lower than reported in this study if they had been included. Another explanation is that gamification was unsuccessful simply because the ABMT procedure lacks intervention credibility, which was inferred from participants’ guess about their treatment allocation. This view was further supported by 2 participants who explicitly stated that they did not understand how clicking on *P*s and *Q*s could help with their pain.

In contrast to the hypothesis that ABMT would yield therapeutic benefit, there were also no differential effects over time between the active ABMT and control conditions on measures of pain intensity, pain interference, anxiety, depression, or perceived improvement. The results add to the mixed literature on the effectiveness of pain ABMT [10,12,16-20]. There was, however, an overall small decrease in depressive symptoms from baseline to posttraining assessment and in pain intensity and pain interference from baseline to follow-up assessment across all conditions. Similar results have been reported in the study by Carleton et al [17], who found a reduction in the experience of pain in both the ABMT and control groups. It is unclear why these reductions were seen in the control condition. It is possible that sham training (ie, the control condition) is not inert and has an active training component (ie, training at 50/50 contingency), which could influence attentional processes [93] or increase attentional control [94]. However, there was no evidence for change in attentional biases or attentional control following training in any of the conditions. Another possibility is that sham training may in fact be training cognitive flexibility [17]. Results could alternatively reflect natural fluctuation of symptoms over time, known as regression to the mean [95].

Treatment expectancies may have accounted, in part, for the observed improvements [96]; although, intervention credibility was low. It is also possible that treatment effects were observed due to improved standard of care or management. The impacts of the COVID-19 pandemic on our results are also unknown. Research has found that COVID-19 pandemic lockdowns affected individuals with chronic pain disproportionately, with increased pain levels and greater effects on mood and physical activity [97]. Although the exact number is unknown, it is certain that some participants started participation during or shortly after a lockdown period, when their levels of pain, pain interference, and depression may have been elevated.

One explanation for the nonsignificant effects is that pretraining attentional biases were not observed in the active ABMT conditions, which makes it difficult to alter them. However, the absence of baseline biases does not mean that ABMT interventions cannot be effective, as there have been reports of samples without an initial bias where ABMT interventions still led to pain-related improvements [12]. A potential explanation for the lack of baseline biases is that attentional bias in chronic pain may not be as strong as attentional bias in anxiety [98], despite our efforts to use stimuli related to pain. Alternatively, attentional biases toward pain may not be a stable trait-like characteristics of individuals with chronic pain, as often presumed, and may vary considerably within and between participants depending on their personal goals and contexts [15]. This would make it difficult to capture them comprehensively or validly with the dot-probe task, which only provides a snapshot of attention at the time when the probe appears. This might explain why the evidence for attentional biases toward pain-related words or pictures has been mixed [5,6,99].

In this study, there was also no evidence that biases changed after training for the active ABMT conditions compared to the control condition. The finding is, although contrary to our hypotheses, consistent with other pain ABMT studies [12,17,20]. Moreover, there was no relationship between changes in attentional bias magnitude from pretraining to posttraining assessment and changes in scores on measures of pain intensity, pain interference, and mood. Another explanation for the null findings is that the effectiveness of ABMT was attenuated by the relatively uncontrolled setting, with the potential presence of multiple distractions (eg, noise) that can impact attentional resources [100] and also influence the RT-based attentional bias index. It is also possible that ABMT does not target attentional bias or other underlying mechanisms (eg, interpretation bias), as suggested by some researchers [101,102]. Our data indicate no evidence of changes in interpretation biases or attentional control following training between the conditions. The findings are consistent with the study by Todd et al [102], which reported no significant effects of ABMT on interpretation bias in healthy undergraduate students under conditions of low threat and with the study by Heathcote et al [20], which reported no training effects on attentional control in adolescents with chronic pain. It should be noted, however, that Todd et al [102] used a more indirect measure of interpretation bias (ie, Incidental Learning Task [103]), whereas the task we used was constrained by a forced-choice response format and did not include control scenarios, which raises questions about whether other processes (eg, priming and negative expectancy bias) may have influenced the scores on this measure.

### Limitations and Future Work

This study has several limitations. First, our sample was restricted to individuals with chronic musculoskeletal pain, thereby limiting the generalizability of findings to other types of pain conditions. Second, despite a rigorous randomization procedure, the conditions differed on several baseline characteristics. Although these were controlled for in our analyses, replications with matched samples are warranted as there might still be some residual confounding. Third, the use of a web-based delivery mode provided limited control over the environment in which ABMT was completed. Given there are suggestions in the anxiety ABMT literature that the efficacy of ABMT may be more difficult to achieve with remote training delivery compared to training in the laboratory [104], future research may want to replicate our findings in a more controlled setting. Fourth, although we opted for the dot-probe assessment paradigm as it is frequently used in web-based attentional bias research [17], we did not assess its reliability, which has been called into question [105]. Future studies may wish to use alternative measures of attentional bias such as eye-tracking technology. Fifth, the selection of a limited number of stimuli (8 stimulus pairs) may have affected the results. Despite our stimulus material consisting of word stimuli that have a strong association to pain, it is possible that a habituation effect to the words reduced their meaningfulness or that it contributed to the decrease in engagement over time. Future research may consider the use of more diverse stimulus types (eg, pictures as well). Finally, we chose to not include a gamified sham (control) condition to maximize power, which limited our ability to determine the impact of cognitive load on the processes underlying ABMT. Future research may consider using a “neutral” or no-contingency control condition such as sham-n training to ascertain whether attentional bias is affected or if cognitive flexibility is trained [93,106]. Future studies would also benefit from using a more specific gamification framework (eg, [107]) that involves an interdisciplinary team of individuals with chronic pain, cognitive experts, and gamification designers to guide the co-design of their interventions.

### Conclusions

In summary, the results of this first study investigating the effects of a gamified web-delivered pain ABMT intervention suggest that it has no additional effects over and above the standard ABMT and sham training control conditions. Adding minimal game elements alone does not seem to be enough to counteract the monotony of the dot-probe task. There is also no robust evidence that ABMT is of additional clinical value for individuals with chronic pain, at least not in its current form. A change of attentional bias, which is the presumed mechanism of action, was also not observed. While theoretically justified, the findings of this study do not provide support for the widespread clinical use of gamified and nongamified pain ABMT paradigms delivered over the internet.

## Appendix

Multimedia Appendix 1 CONSORT-EHEALTH (Consolidated Standards of Reporting Trials of Electronic and Mobile Health Applications and Online Telehealth) checklist.

Multimedia Appendix 2 Additional and exploratory analyses.

Multimedia Appendix 3 Primary and secondary outcomes.

## Abbreviations

ABMT: attentional bias modification training
ACS: Attentional Control Scale
AIC: Akaike information criterion
BIC: Bayesian information criterion
CONSORT: Consolidated Standards of Reporting Trials
CONSORT-EHEALTH: Consolidated Standards of Reporting Trials of Electronic and Mobile Health Applications and Online Telehealth
GCPS: Graded Chronic Pain Scale
IMI: Intrinsic Motivation Inventory
PGIC: Patient Global Impression of Change
PROMIS: Patient-Reported Outcomes Measurement Information System
RT: reaction time

## Data Availability

The datasets generated and analyzed during this study are available from the corresponding author on reasonable request.
