# Supplementary material for: Gamified Web-Delivered Attentional Bias Modification Training for Adults With Chronic Pain: Randomized, Double-Blind, Placebo-Controlled Trial
Source: JMIR Serious Games. 2025 Jan 16;13:e50635. doi: 10.2196/50635 (PMC11783034; doi:10.2196/50635)
Supplement: Multimedia Appendix 1 [file games_v13i1e50635_app1.pdf]

CONSORT-EHEALTH (V 1.6.1) - Submission/Publication Form

The CONSORT-EHEALTH checklist is intended for authors of randomized trials evaluating web-based and Internet-based applications/interventions, including mobile interventions, electronic games (incl multiplayer games), social media, certain telehealth applications, and other interactive and/or networked electronic applications. Some of the items (e.g. all subitems under item 5 - description of the intervention) may also be applicable for other study designs.

The goal of the CONSORT EHEALTH checklist and guideline is to be
a) a guide for reporting for authors of RCTs,
b) to form a basis for appraisal of an ehealth trial (in terms of validity)

CONSORT-EHEALTH items/subitems are MANDATORY reporting items for studies published in the Journal of Medical Internet Research and other journals / scientific societies endorsing the checklist.

Items numbered 1., 2., 3., 4a., 4b etc are original CONSORT or CONSORT-NPT (non-pharmacologic treatment) items.
Items with Roman numerals (i., ii, iii, iv etc.) are CONSORT-EHEALTH extensions/clarifications.

As the CONSORT-EHEALTH checklist is still considered in a formative stage, we would ask that you also RATE ON A SCALE OF 1-5 how important/useful you feel each item is FOR THE PURPOSE OF THE CHECKLIST and reporting guideline (optional).

Mandatory reporting items are marked with a red *.
In the textboxes, either copy & paste the relevant sections from your manuscript into this form - please include any quotes from your manuscript in QUOTATION MARKS,
or answer directly by providing additional information not in the manuscript, or elaborating on why the item was not relevant for this study.

YOUR ANSWERS WILL BE PUBLISHED AS A SUPPLEMENTARY FILE TO YOUR PUBLICATION IN JMIR AND ARE CONSIDERED PART OF YOUR PUBLICATION (IF ACCEPTED).
Please fill in these questions diligently. Information will not be copyedited, so please use proper spelling and grammar, use correct capitalization, and avoid abbreviations.

DO NOT FORGET TO SAVE AS PDF _AND_ CLICK THE SUBMIT BUTTON SO YOUR ANSWERS ARE IN OUR DATABASE !!!

Citation Suggestion (if you append the pdf as Appendix we suggest to cite this paper in the caption):
Eysenbach G, CONSORT-EHEALTH Group
CONSORT-EHEALTH: Improving and Standardizing Evaluation Reports of Web-based and Mobile Health Interventions
J Med Internet Res 2011;13(4):e126

URL: http://www.jmir.org/2011/4/e126/
doi: 10.2196/jmir.1923
PMID: 22209829

Not shared

Draft saved

* Indicates required question

Your name *

First Last

Julie F. Vermeir

Primary Affiliation (short), City, Country *

University of Toronto, Toronto, Canada

Queensland University of Technology, Brisbane

Your e-mail address *

abc@gmail.com

julie.vermeir@hdr.qut.edu.au

Title of your manuscript *

Provide the (draft) title of your manuscript.

Gamified Web-Delivered Attentional Bias Modification Training for Adults With Chronic Pain: Randomized, Double-Blind, Placebo-Controlled Trial

Name of your App/Software/Intervention *

If there is a short and a long/alternate name, write the short name first and add the long name in brackets.

Gamified web-delivered attentional bias modifi

Evaluated Version (if any)

e.g. "V1", "Release 2017-03-01", "Version 2.0.27913"

Your answer

Language(s) *

What language is the intervention/app in? If multiple languages are available, separate by comma (e.g. "English, French")

English

URL of your Intervention Website or App

e.g. a direct link to the mobile app on app in appstore (itunes, Google Play), or URL of the website. If the intervention is a DVD or hardware, you can also link to an Amazon page.

Your answer

URL of an image/screenshot (optional)

Your answer

Accessibility *

Can an enduser access the intervention presently?

- ○ access is free and open
- ○ access only for special usergroups, not open
- ○ access is open to everyone, but requires payment/subscription/in-app purchases
- ◉ app/intervention no longer accessible
- ○ Other:

Primary Medical Indication/Disease/Condition *

e.g. "Stress", "Diabetes", or define the target group in brackets after the condition, e.g. "Autism (Parents of children with)", "Alzheimers (Informal Caregivers of)"

Adults with chronic pain

Primary Outcomes measured in trial *

comma-separated list of primary outcomes reported in the trial

Self-reported and behavioral engagement, pair

Secondary/other outcomes

Are there any other outcomes the intervention is expected to affect?

Anxiety, depression, cognitive biases, and perceived improvement

Recommended "Dose" *

What do the instructions for users say on how often the app should be used?

- ○ Approximately Daily
- ○ Approximately Weekly
- ○ Approximately Monthly
- ○ Approximately Yearly
- ○ "as needed"
- ◉ Other: Twice a week on a separate pair of days (Monday and Thursday or Tu

Approx. Percentage of Users (starters) still using the app as recommended after 3 months *

- ○ unknown / not evaluated
- ○ 0-10%
- ○ 11-20%
- ○ 21-30%
- ○ 31-40%
- ○ 41-50%
- ○ 51-60%
- ○ 61-70%
- ○ 71%-80%
- ○ 81-90%
- ○ 91-100%
- ◉ Other: Users only had access to the intervention during the study

Overall, was the app/intervention effective? *

- ( ) yes: all primary outcomes were significantly better in intervention group vs control
- ( ) partly: SOME primary outcomes were significantly better in intervention group vs control
- (•) no statistically significant difference between control and intervention
- ( ) potentially harmful: control was significantly better than intervention in one or more outcomes
- ( ) inconclusive: more research is needed
- ( ) Other:

Article Preparation Status/Stage *

At which stage in your article preparation are you currently (at the time you fill in this form)

- ( ) not submitted yet - in early draft status
- ( ) not submitted yet - in late draft status, just before submission
- ( ) submitted to a journal but not reviewed yet
- ( ) submitted to a journal and after receiving initial reviewer comments
- (•) submitted to a journal and accepted, but not published yet
- ( ) published
- ( ) Other:

Journal *

If you already know where you will submit this paper (or if it is already submitted), please provide the journal name (if it is not JMIR, provide the journal name under "other")

- ○ not submitted yet / unclear where I will submit this
- ○ Journal of Medical Internet Research (JMIR)
- ○ JMIR mHealth and UHealth
- ◉ JMIR Serious Games
- ○ JMIR Mental Health
- ○ JMIR Public Health
- ○ JMIR Formative Research
- ○ Other JMIR sister journal
- ○ Other:

Is this a full powered effectiveness trial or a pilot/feasibility trial? *

- ○ Pilot/feasibility
- ◉ Fully powered

Manuscript tracking number *

If this is a JMIR submission, please provide the manuscript tracking number under "other" (The ms tracking number can be found in the submission acknowledgement email, or when you login as author in JMIR. If the paper is already published in JMIR, then the ms tracking number is the four-digit number at the end of the DOI, to be found at the bottom of each published article in JMIR)

- ○ no ms number (yet) / not (yet) submitted to / published in JMIR
- ◉ Other: 50635

TITLE AND ABSTRACT

1a) TITLE: Identification as a randomized trial in the title

1a) Does your paper address CONSORT item 1a? *

I.e does the title contain the phrase "Randomized Controlled Trial"? (if not, explain the reason under "other")

- (x) yes
- ( ) Other:

1a-i) Identify the mode of delivery in the title

Identify the mode of delivery. Preferably use “web-based” and/or “mobile” and/or “electronic game” in the title. Avoid ambiguous terms like “online”, “virtual”, “interactive”. Use “Internet-based” only if Intervention includes non-web-based Internet components (e.g. email), use “computer-based” or “electronic” only if offline products are used. Use “virtual” only in the context of “virtual reality” (3-D worlds). Use “online” only in the context of “online support groups”. Complement or substitute product names with broader terms for the class of products (such as “mobile” or “smart phone” instead of “iphone”), especially if the application runs on different platforms.

	1	2	3	4	5	
subitem not at all important	( )	( )	( )	( )	(x)	essential

Clear selection

Does your paper address subitem 1a-i? *

Copy and paste relevant sections from manuscript title (include quotes in quotation marks "like this" to indicate direct quotes from your manuscript), or elaborate on this item by providing additional information not in the ms, or briefly explain why the item is not applicable/relevant for your study

"Web-Delivered Attentional Bias Modification Training"

1a-ii) Non-web-based components or important co-interventions in title

Mention non-web-based components or important co-interventions in title, if any (e.g., “with telephone support”).

	1	2	3	4	5	
subitem not at all important	○	●	○	○	○	essential

Clear selection

Does your paper address subitem 1a-ii?

Copy and paste relevant sections from manuscript title (include quotes in quotation marks "like this" to indicate direct quotes from your manuscript), or elaborate on this item by providing additional information not in the ms, or briefly explain why the item is not applicable/relevant for your study

Only "Web-Delivered" intervention

1a-iii) Primary condition or target group in the title

Mention primary condition or target group in the title, if any (e.g., “for children with Type I Diabetes”) Example: A Web-based and Mobile Intervention with Telephone Support for Children with Type I Diabetes: Randomized Controlled Trial

	1	2	3	4	5	
subitem not at all important	○	○	○	○	●	essential

Clear selection

Does your paper address subitem 1a-iii? *

Copy and paste relevant sections from manuscript title (include quotes in quotation marks "like this" to indicate direct quotes from your manuscript), or elaborate on this item by providing additional information not in the ms, or briefly explain why the item is not applicable/relevant for your study

"for Adults With Chronic Pain"

1b) ABSTRACT: Structured summary of trial design, methods, results, and conclusions

NPT extension: Description of experimental treatment, comparator, care providers, centers, and blinding status.

1b-i) Key features/functionalities/components of the intervention and comparator in the METHODS section of the ABSTRACT

Mention key features/functionalities/components of the intervention and comparator in the abstract. If possible, also mention theories and principles used for designing the site. Keep in mind the needs of systematic reviewers and indexers by including important synonyms. (Note: Only report in the abstract what the main paper is reporting. If this information is missing from the main body of text, consider adding it)

	1	2	3	4	5	
subitem not at all important	○	○	○	◉	○	essential

Clear selection

Does your paper address subitem 1b-i? *

Copy and paste relevant sections from the manuscript abstract (include quotes in quotation marks "like this" to indicate direct quotes from your manuscript), or elaborate on this item by providing additional information not in the ms, or briefly explain why the item is not applicable/relevant for your study

"Participants were randomly assigned to complete 6 web-based sessions of nongamified standard ABMT (n=43), gamified ABMT (n=41), or a control condition (nongamified sham ABMT; n=45) over a period of 3 weeks. Active ABMT conditions trained attention away from pain-related words. The gamified task included a combination of 5 game elements."

1b-ii) Level of human involvement in the METHODS section of the ABSTRACT

Clarify the level of human involvement in the abstract, e.g., use phrases like "fully automated" vs. "therapist/nurse/care provider/physician-assisted" (mention number and expertise of providers involved, if any). (Note: Only report in the abstract what the main paper is reporting. If this information is missing from the main body of text, consider adding it)

	1	2	3	4	5	
subitem not at all important	○	○	○	◉	○	essential

Clear selection

Does your paper address subitem 1b-ii?

Copy and paste relevant sections from the manuscript abstract (include quotes in quotation marks "like this" to indicate direct quotes from your manuscript), or elaborate on this item by providing additional information not in the ms, or briefly explain why the item is not applicable/relevant for your study

Abstract: "assigned to complete 6 web-based sessions ". Manuscript: "All training sessions were conducted via the internet at the participants' time and place of convenience using their own computers, and all outcome assessments (ie, at baseline, during training, immediately after training, and at 1-month posttraining) were self-assessed via web-based questionnaires and computerized tasks."

1b-iii) Open vs. closed, web-based (self-assessment) vs. face-to-face assessments in the METHODS section of the ABSTRACT

Mention how participants were recruited (online vs. offline), e.g., from an open access website or from a clinic or a closed online user group (closed usergroup trial), and clarify if this was a purely web-based trial, or there were face-to-face components (as part of the intervention or for assessment). Clearly say if outcomes were self-assessed through questionnaires (as common in web-based trials). Note: In traditional offline trials, an open trial (open-label trial) is a type of clinical trial in which both the researchers and participants know which treatment is being administered. To avoid confusion, use “blinded” or “unblinded” to indicated the level of blinding instead of “open”, as “open” in web-based trials usually refers to “open access” (i.e. participants can self-enrol). (Note: Only report in the abstract what the main paper is reporting. If this information is missing from the main body of text, consider adding it)

	1	2	3	4	5	
subitem not at all important	○	○	○	◉	○	essential

Clear selection

Does your paper address subitem 1b-iii?

Copy and paste relevant sections from the manuscript abstract (include quotes in quotation marks "like this" to indicate direct quotes from your manuscript), or elaborate on this item by providing additional information not in the ms, or briefly explain why the item is not applicable/relevant for your study

Abstract: "recruited from clinical (hospital outpatient waiting list) and nonclinical (wider community) settings". Manuscript: "All training sessions were conducted via the internet at the participants’ time and place of convenience using their own computers, and all outcome assessments (ie, at baseline, during training, immediately after training, and at 1-month posttraining) were self-assessed via web-based questionnaires and computerized tasks."

1b-iv) RESULTS section in abstract must contain use data

Report number of participants enrolled/assessed in each group, the use/uptake of the intervention (e.g., attrition/adherence metrics, use over time, number of logins etc.), in addition to primary/secondary outcomes. (Note: Only report in the abstract what the main paper is reporting. If this information is missing from the main body of text, consider adding it)

	1	2	3	4	5	
subitem not at all important	○	○	○	○	◉	essential

Clear selection

Does your paper address subitem 1b-iv?

Copy and paste relevant sections from the manuscript abstract (include quotes in quotation marks "like this" to indicate direct quotes from your manuscript), or elaborate on this item by providing additional information not in the ms, or briefly explain why the item is not applicable/relevant for your study

"A final sample of 129 adults with chronic musculoskeletal pain". "Participants were randomly assigned to complete 6 web-based sessions of nongamified standard ABMT (n=43), gamified ABMT (n=41), or a control condition (nongamified sham ABMT; n=45) over a period of 3 weeks"

1b-v) CONCLUSIONS/DISCUSSION in abstract for negative trials

Conclusions/Discussions in abstract for negative trials: Discuss the primary outcome - if the trial is negative (primary outcome not changed), and the intervention was not used, discuss whether negative results are attributable to lack of uptake and discuss reasons. (Note: Only report in the abstract what the main paper is reporting. If this information is missing from the main body of text, consider adding it)

	1	2	3	4	5	
subitem not at all important	○	○	○	○	◉	essential

Clear selection

Does your paper address subitem 1b-v?

Copy and paste relevant sections from the manuscript abstract (include quotes in quotation marks "like this" to indicate direct quotes from your manuscript), or elaborate on this item by providing additional information not in the ms, or briefly explain why the item is not applicable/relevant for your study

"These findings suggest that gamification, in this context, was not effective at enhancing engagement, and they do not support the widespread clinical use of web-delivered ABMT in treating individuals with chronic musculoskeletal pain."

INTRODUCTION

2a) In INTRODUCTION: Scientific background and explanation of rationale

2a-i) Problem and the type of system/solution

Describe the problem and the type of system/solution that is object of the study: intended as stand-alone intervention vs. incorporated in broader health care program? Intended for a particular patient population? Goals of the intervention, e.g., being more cost-effective to other interventions, replace or complement other solutions? (Note: Details about the intervention are provided in "Methods" under 5)

	1	2	3	4	5	
subitem not at all important	○	○	○	○	◉	essential

Clear selection

Does your paper address subitem 2a-i? *

Copy and paste relevant sections from the manuscript (include quotes in quotation marks "like this" to indicate direct quotes from your manuscript), or elaborate on this item by providing additional information not in the ms, or briefly explain why the item is not applicable/relevant for your study

"ABMT has been proposed as a promising tool for chronic pain based on its successful use for various conditions such as anxiety [13] and depression [14]. So far, however, results of pain ABMT interventions in pain (refer to the study by Van Ryckeghem et al [15] for an overview) and chronic pain samples [10,12,16-20] have been mixed, with most studies reporting at least some short- to medium-term therapeutic benefits of ABMT for clinically relevant pain outcomes [10,12,16,18,19]. There are 2 factors that may contribute to these mixed findings are boredom and low motivation. ABMT procedures require participants to complete numerous trials over multiple sessions across several weeks [10,12,16-20] and typically use a basic layout. There is evidence that participants view the dot-probe task as monotonous, repetitive, and boring [21-23]. Consequently, this may lead to (temporal) low task engagement, low motivation to complete the sessions, and high dropout rates, which in turn may compromise the effectiveness of the intervention. Increasing engagement through the gamification of ABMT may provide the opportunity to overcome some of these barriers. Gamification refers to the use of digital game elements (eg, points) in nonentertainment settings [24]. Several reviews on gamified cognitive training tasks have reported that adding game-like elements to repetitive tasks improves motivation and engagement [25,26]. This is further supported by a recent study investigating gamification of interpretation bias modification for anxiety, which found that gamification could increase engagement and enjoyment [27]. However, it is still difficult to draw any firm conclusions regarding the effectiveness of gamified cognitive bias modification interventions [27,28], and more rigorously designed and theory-driven research is needed, particularly in the field of chronic pain."

2a-ii) Scientific background, rationale: What is known about the (type of) system

Scientific background, rationale: What is known about the (type of) system that is the object of the study (be sure to discuss the use of similar systems for other conditions/diagnoses, if appropiate), motivation for the study, i.e. what are the reasons for and what is the context for this specific study, from which stakeholder viewpoint is the study performed, potential impact of findings [2]. Briefly justify the choice of the comparator.

	1	2	3	4	5	
subitem not at all important	○	○	○	◉	○	essential

Clear selection

Does your paper address subitem 2a-ii? *

Copy and paste relevant sections from the manuscript (include quotes in quotation marks "like this" to indicate direct quotes from your manuscript), or elaborate on this item by providing additional information not in the ms, or briefly explain why the item is not applicable/relevant for your study

"ABMT has been proposed as a promising tool for chronic pain based on its successful use for various conditions such as anxiety [13] and depression [14]. So far, however, results of pain ABMT interventions in pain (refer to the study by Van Ryckeghem et al [15] for an overview) and chronic pain samples [10,12,16-20] have been mixed, with most studies reporting at least some short- to medium-term therapeutic benefits of ABMT for clinically relevant pain outcomes [10,12,16,18,19]. There are 2 factors that may contribute to these mixed findings are boredom and low motivation. ABMT procedures require participants to complete numerous trials over multiple sessions across several weeks [10,12,16-20] and typically use a basic layout. There is evidence that participants view the dot-probe task as monotonous, repetitive, and boring [21-23]. Consequently, this may lead to (temporal) low task engagement, low motivation to complete the sessions, and high dropout rates, which in turn may compromise the effectiveness of the intervention. Increasing engagement through the gamification of ABMT may provide the opportunity to overcome some of these barriers. Gamification refers to the use of digital game elements (eg, points) in nonentertainment settings [24]. Several reviews on gamified cognitive training tasks have reported that adding game-like elements to repetitive tasks improves motivation and engagement [25,26]. This is further supported by a recent study investigating gamification of interpretation bias modification for anxiety, which found that gamification could increase engagement and enjoyment [27]. However, it is still difficult to draw any firm conclusions regarding the effectiveness of gamified cognitive bias modification interventions [27,28], and more rigorously designed and theory-driven research is needed, particularly in the field of chronic pain."

2b) In INTRODUCTION: Specific objectives or hypotheses

Does your paper address CONSORT subitem 2b? *

Copy and paste relevant sections from the manuscript (include quotes in quotation marks "like this" to indicate direct quotes from your manuscript), or elaborate on this item by providing additional information not in the ms, or briefly explain why the item is not applicable/relevant for your study

"The aim of this study was to investigate the effects of a gamified web-delivered ABMT intervention using an empirically supported set of pain-related word stimuli on behavioral and self-reported engagement, pain intensity, pain interference, anxiety, depression, cognitive biases, and perceived improvement in a sample of adults with chronic musculoskeletal pain. We hypothesized that (1) the gamified ABMT condition would be more engaging than the nongamified (ie, standard ABMT and control) conditions; (2) both the standard and gamified ABMT conditions, compared to the control condition, would be more effective in improving outcomes of interest over time; and (3) these improvements would be greater in the gamified ABMT condition compared to the standard ABMT condition [29]."

METHODS

3a) Description of trial design (such as parallel, factorial) including allocation ratio

Does your paper address CONSORT subitem 3a? *

Copy and paste relevant sections from the manuscript (include quotes in quotation marks "like this" to indicate direct quotes from your manuscript), or elaborate on this item by providing additional information not in the ms, or briefly explain why the item is not applicable/relevant for your study

"This study was a randomized, double-blind, placebo-controlled, 3-arm, parallel-group trial examining the efficacy of a gamified web-delivered ABMT for chronic pain." "A block randomization technique was used, allowing 6 participants at a time to be randomized in equal proportions to the 3 training arms."

3b) Important changes to methods after trial commencement (such as eligibility criteria), with reasons

Does your paper address CONSORT subitem 3b? *

Copy and paste relevant sections from the manuscript (include quotes in quotation marks "like this" to indicate direct quotes from your manuscript), or elaborate on this item by providing additional information not in the ms, or briefly explain why the item is not applicable/relevant for your study

No important changes were made after trial commencement.

3b-i) Bug fixes, Downtimes, Content Changes

Bug fixes, Downtimes, Content Changes: ehealth systems are often dynamic systems. A description of changes to methods therefore also includes important changes made on the intervention or comparator during the trial (e.g., major bug fixes or changes in the functionality or content) (5-iii) and other "unexpected events" that may have influenced study design such as staff changes, system failures/downtimes, etc. [2].

	1	2	3	4	5	
subitem not at all important	○	○	○	◉	○	essential

Clear selection

Does your paper address subitem 3b-i?

Copy and paste relevant sections from the manuscript (include quotes in quotation marks "like this" to indicate direct quotes from your manuscript), or elaborate on this item by providing additional information not in the ms, or briefly explain why the item is not applicable/relevant for your study

No important changes were made on the intervention or comparator during the trial. However, we do acknowledge in our manuscript that the impacts of the COVID-19 pandemic on our results are unknown.

4a) Eligibility criteria for participants

Does your paper address CONSORT subitem 4a? *

Copy and paste relevant sections from the manuscript (include quotes in quotation marks "like this" to indicate direct quotes from your manuscript), or elaborate on this item by providing additional information not in the ms, or briefly explain why the item is not applicable/relevant for your study

Inclusion criteria
- Aged ≥18 years
- Experiencing chronic musculoskeletal pain, that is, pain in bones, joints, muscles, or related soft tissues (eg, rheumatoid arthritis pain, nonspecific back pain, or fibromyalgia pain)
- Meeting the criteria for chronic pain, that is, self-reported pain that lasts or recurs for >3 months [31]
- Having normal or corrected-to-normal (eg, glasses or contact lenses) vision

Exclusion criteria
- Not being a native English speaker or fluent in reading and writing English (as participants' reaction time to English words was used as an index of attentional bias to semantically related pain memory networks)
- Not having access to a desktop or laptop computer connected to reliable internet (as the trial was conducted on the web)
- Not being able or willing to provide informed consent to participate

4a-i) Computer / Internet literacy

Computer / Internet literacy is often an implicit “de facto” eligibility criterion - this should be explicitly clarified.

	1	2	3	4	5	
subitem not at all important	○	○	○	○	◉	essential

Clear selection

Does your paper address subitem 4a-i?

Copy and paste relevant sections from the manuscript (include quotes in quotation marks "like this" to indicate direct quotes from your manuscript), or elaborate on this item by providing additional information not in the ms, or briefly explain why the item is not applicable/relevant for your study

Participants were excluded if "Not having access to a desktop or laptop computer connected to reliable internet (as the trial was conducted on the web)" and "Not being able or willing to provide informed consent to participate"

4a-ii) Open vs. closed, web-based vs. face-to-face assessments:

Open vs. closed, web-based vs. face-to-face assessments: Mention how participants were recruited (online vs. offline), e.g., from an open access website or from a clinic, and clarify if this was a purely web-based trial, or there were face-to-face components (as part of the intervention or for assessment), i.e., to what degree got the study team to know the participant. In online-only trials, clarify if participants were quasi-anonymous and whether having multiple identities was possible or whether technical or logistical measures (e.g., cookies, email confirmation, phone calls) were used to detect/prevent these.

	1	2	3	4	5	
subitem not at all important	○	○	○	○	◉	essential

Clear selection

Does your paper address subitem 4a-ii? *

Copy and paste relevant sections from the manuscript (include quotes in quotation marks "like this" to indicate direct quotes from your manuscript), or elaborate on this item by providing additional information not in the ms, or briefly explain why the item is not applicable/relevant for your study

"Participants were recruited from a large Australian public hospital outpatient waitlist for pain management (clinical setting) and from the wider community (nonclinical setting). Individuals on the hospital outpatient waiting list were invited to participate through personalized mail correspondence, whereas individuals from the wider community were recruited through university electronic mailing lists, social media, and community channels (eg, Facebook advertising, Pain Australia, Chronic Pain Australia, and word of mouth)."

4a-iii) Information giving during recruitment

Information given during recruitment. Specify how participants were briefed for recruitment and in the informed consent procedures (e.g., publish the informed consent documentation as appendix, see also item X26), as this information may have an effect on user self-selection, user expectation and may also bias results.

	1	2	3	4	5	
subitem not at all important	○	○	○	◉	○	essential

Clear selection

Does your paper address subitem 4a-iii?

Copy and paste relevant sections from the manuscript (include quotes in quotation marks "like this" to indicate direct quotes from your manuscript), or elaborate on this item by providing additional information not in the ms, or briefly explain why the item is not applicable/relevant for your study

Published protocol: "All recruitment materials (eg, hospital personalized letters and flyers) will include a survey hyperlink that will direct prospective participants to the necessary study information to decide on participation. This will include information about the research procedures; the voluntary nature of the study, with the freedom to withdraw at any time until the collected data are deidentified (ie, upon completion of the 1-month follow-up survey); the potential risks and benefits of their participation; and whom to contact for questions about the research. All interested participants will be asked to provide informed consent electronically (ie, e-consent) before being taken to the screening questions and then to the first screen of the baseline assessment."

4b) Settings and locations where the data were collected

Does your paper address CONSORT subitem 4b? *

Copy and paste relevant sections from the manuscript (include quotes in quotation marks "like this" to indicate direct quotes from your manuscript), or elaborate on this item by providing additional information not in the ms, or briefly explain why the item is not applicable/relevant for your study

"All training sessions were conducted via the internet at the participants' time and place of convenience using their own computers, and all outcome assessments (ie, at baseline, during training, immediately after training, and at 1-month posttraining) were self-assessed via web-based questionnaires and computerized tasks."

4b-i) Report if outcomes were (self-)assessed through online questionnaires

Clearly report if outcomes were (self-)assessed through online questionnaires (as common in web-based trials) or otherwise.

	1	2	3	4	5	
subitem not at all important	○	○	○	○	◉	essential

Clear selection

Does your paper address subitem 4b-i? *

Copy and paste relevant sections from the manuscript (include quotes in quotation marks "like this" to indicate direct quotes from your manuscript), or elaborate on this item by providing additional information not in the ms, or briefly explain why the item is not applicable/relevant for your study

"All training sessions were conducted via the internet at the participants' time and place of convenience using their own computers, and all outcome assessments (ie, at baseline, during training, immediately after training, and at 1-month posttraining) were self-assessed via web-based questionnaires and computerized tasks."

4b-ii) Report how institutional affiliations are displayed

Report how institutional affiliations are displayed to potential participants [on ehealth media], as affiliations with prestigious hospitals or universities may affect volunteer rates, use, and reactions with regards to an intervention.(Not a required item – describe only if this may bias results)

	1	2	3	4	5	
subitem not at all important	○	○	●	○	○	essential

Clear selection

Does your paper address subitem 4b-ii?

Copy and paste relevant sections from the manuscript (include quotes in quotation marks "like this" to indicate direct quotes from your manuscript), or elaborate on this item by providing additional information not in the ms, or briefly explain why the item is not applicable/relevant for your study

The university and hospital logos were displayed on all the recruitment materials.

5) The interventions for each group with sufficient details to allow replication, including how and when they were actually administered

5-i) Mention names, credential, affiliations of the developers, sponsors, and owners

Mention names, credential, affiliations of the developers, sponsors, and owners [6] (if authors/evaluators are owners or developer of the software, this needs to be declared in a “Conflict of interest” section or mentioned elsewhere in the manuscript).

	1	2	3	4	5	
subitem not at all important	○	○	○	●	○	essential

Clear selection

Does your paper address subitem 5-i?

Copy and paste relevant sections from the manuscript (include quotes in quotation marks "like this" to indicate direct quotes from your manuscript), or elaborate on this item by providing additional information not in the ms, or briefly explain why the item is not applicable/relevant for your study

There was no conflict of interest in this study.

5-ii) Describe the history/development process

Describe the history/development process of the application and previous formative evaluations (e.g., focus groups, usability testing), as these will have an impact on adoption/use rates and help with interpreting results.

	1	2	3	4	5	
subitem not at all important	○	○	◉	○	○	essential

Clear selection

Does your paper address subitem 5-ii?

Copy and paste relevant sections from the manuscript (include quotes in quotation marks "like this" to indicate direct quotes from your manuscript), or elaborate on this item by providing additional information not in the ms, or briefly explain why the item is not applicable/relevant for your study

Protocol: Gamified ABMT
"The implementation of gamification has been listed within the group of complex interventions [30]. These interventions refer to activities that comprise multiple interacting components (eg, intensity and setting) that, when applied to the target population, result in a range of possible outcomes [31]. Therefore, the development of the gamified task followed the Medical Research Council framework for complex interventions [31], using theory, review evidence, and expert involvement. The gamified ABMT task was developed in several steps.

In the first step, a multidisciplinary team with research expertise in the fields of eHealth, cognitive psychology, and gamification discussed the core theories, methods, mode of delivery, implementation strategies, and design requirements. Specifically, rather than developing a completely new ABMT intervention, it was decided to design the gamified ABMT task as a so-called game-shell [32]; that is, game elements were added as an additional layer to the standard ABMT task without changing the initial structure. This design allows the original evidence-based ABMT paradigm to remain unchanged and has been frequently used in the gamified cognitive literature [17,32,33].

In step 2, the game elements were selected. This was guided by a qualitative and quantitative review, as well as specific theories. First, we undertook a systematic review and meta-analysis assessing the effectiveness of gamification applied to cognitive training tasks to gain a better understanding of the impact of gamification on cognitive training and identify factors that contribute to the optimal design of such programs [15]. The review identified that typically 5 game elements were used and that achievement and progression-oriented game features (eg, rewards and feedback loops) were commonly implemented in cognitive training tasks. Although the review could not show support for one feature over another (because of a limited number of studies in the subgroups), it provided evidence for the effectiveness of gamification in improving motivation and/or engagement (Hedges g=0.72) and synthesized findings into practical guidelines for implementing gamification for cognitive training.

Second, to increase the likelihood of the effectiveness of the intervention design and respond to the call for more theory-driven research on gamification in the field of health [15,34,35], the implementation of game elements in the ABMT procedure was guided by concepts of self-determination theory [36,37] and self-regulation [38]. According to self-determination theory [36,37], which is a well-established theoretical framework within gamification research [34], competence (ie, feeling effective), relatedness (ie, feeling connected to others), and autonomy (ie, feeling a sense of freedom) are the 3 basic psychological needs that determine intrinsic motivation, sustained engagement, and psychological well-being. Previous research has shown that these needs can be addressed by specific elements such as badges, leaderboards, performance graphs, and social competition [39-41]. Another construct that is fundamental to the success of health-related interventions is self-regulation, defined as a dynamic motivational process of setting, pursuing, and maintaining personal goals [42-44]. Self-regulation techniques such as goal setting and self-monitoring can motivate users to engage and sustain in activities, and there is evidence that these techniques can be facilitated in gamification through a range of features such as rewards, goals, levels, and progress bars [45-47]."

"In step 3, a gamified prototype for the experimental condition was created collaboratively by the authors of this paper, who are experts in the field of gamification and cognitive psychology, and programmed using Inquisit 6.4.

In the last step, all the intervention materials and tasks (nongamified and gamified) were

[illegible]
piloted internally by members of the research team to ensure that the program was feasible to deliver over the internet to the target sample. Following discussions, minor refinements were proposed and made to the gamified intervention. For example, the width and height of the progress bar were adjusted to make the visibility of the progression more noticeable."

5-iii) Revisions and updating

Revisions and updating. Clearly mention the date and/or version number of the application/intervention (and comparator, if applicable) evaluated, or describe whether the intervention underwent major changes during the evaluation process, or whether the development and/or content was “frozen” during the trial. Describe dynamic components such as news feeds or changing content which may have an impact on the replicability of the intervention (for unexpected events see item 3b).

	1	2	3	4	5	
subitem not at all important	○	○	●	○	○	essential

Clear selection

Does your paper address subitem 5-iii?

Copy and paste relevant sections from the manuscript (include quotes in quotation marks "like this" to indicate direct quotes from your manuscript), or elaborate on this item by providing additional information not in the ms, or briefly explain why the item is not applicable/relevant for your study

Protocol:
"In the last step, all the intervention materials and tasks (nongamified and gamified) were piloted internally by members of the research team to ensure that the program was feasible to deliver over the internet to the target sample. Following discussions, minor refinements were proposed and made to the gamified intervention. For example, the width and height of the progress bar were adjusted to make the visibility of the progression more noticeable."
No revisions or updating were made during the intervention.

5-iv) Quality assurance methods

Provide information on quality assurance methods to ensure accuracy and quality of information provided [1], if applicable.

	1	2	3	4	5	
subitem not at all important	○	○	○	○	○	essential

Does your paper address subitem 5-iv?

Copy and paste relevant sections from the manuscript (include quotes in quotation marks "like this" to indicate direct quotes from your manuscript), or elaborate on this item by providing additional information not in the ms, or briefly explain why the item is not applicable/relevant for your study

Your answer

5-v) Ensure replicability by publishing the source code, and/or providing screenshots/screen-capture video, and/or providing flowcharts of the algorithms used

Ensure replicability by publishing the source code, and/or providing screenshots/screen-capture video, and/or providing flowcharts of the algorithms used. Replicability (i.e., other researchers should in principle be able to replicate the study) is a hallmark of scientific reporting.

	1	2	3	4	5	
subitem not at all important	○	○	○	◉	○	essential

Clear selection

Does your paper address subitem 5-v?

Copy and paste relevant sections from the manuscript (include quotes in quotation marks "like this" to indicate direct quotes from your manuscript), or elaborate on this item by providing additional information not in the ms, or briefly explain why the item is not applicable/relevant for your study

The game elements used can be found in the manuscript.
Description of the intervention is provided in detail in the published protocol and in the manuscript which would allow other researchers to replicate the study.

5-vi) Digital preservation

Digital preservation: Provide the URL of the application, but as the intervention is likely to change or disappear over the course of the years; also make sure the intervention is archived (Internet Archive, webcitation.org, and/or publishing the source code or screenshots/videos alongside the article). As pages behind login screens cannot be archived, consider creating demo pages which are accessible without login.

	1	2	3	4	5	
subitem not at all important	○	○	○	○	○	essential

Does your paper address subitem 5-vi?

Copy and paste relevant sections from the manuscript (include quotes in quotation marks "like this" to indicate direct quotes from your manuscript), or elaborate on this item by providing additional information not in the ms, or briefly explain why the item is not applicable/relevant for your study

Your answer

5-vii) Access

Access: Describe how participants accessed the application, in what setting/context, if they had to pay (or were paid) or not, whether they had to be a member of specific group. If known, describe how participants obtained “access to the platform and Internet” [1]. To ensure access for editors/reviewers/readers, consider to provide a “backdoor” login account or demo mode for reviewers/readers to explore the application (also important for archiving purposes, see vi).

	1	2	3	4	5	
subitem not at all important	○	○	○	◉	○	essential

Clear selection

Does your paper address subitem 5-vii? *

Copy and paste relevant sections from the manuscript (include quotes in quotation marks "like this" to indicate direct quotes from your manuscript), or elaborate on this item by providing additional information not in the ms, or briefly explain why the item is not applicable/relevant for your study

"At the end of the assessment, participants selected their preferred days for training (Monday and Thursday or Tuesday and Friday) and provided an email address so that the research team could send links to the training sessions. Participants who completed the baseline assessment were randomized into 1 of 3 conditions (standard ABMT, gamified ABMT, or control) and invited by email to start their first training session. This email included a web link to the appropriate version of the training as well as instructions on how to download and install the software used to run the program."

5-viii) Mode of delivery, features/functionalities/components of the intervention and comparator, and the theoretical framework

Describe mode of delivery, features/functionalities/components of the intervention and comparator, and the theoretical framework [6] used to design them (instructional strategy [1], behaviour change techniques, persuasive features, etc., see e.g., [7, 8] for terminology). This includes an in-depth description of the content (including where it is coming from and who developed it) [1],” whether [and how] it is tailored to individual circumstances and allows users to track their progress and receive feedback” [6]. This also includes a description of communication delivery channels and – if computer-mediated communication is a component – whether communication was synchronous or asynchronous [6]. It also includes information on presentation strategies [1], including page design principles, average amount of text on pages, presence of hyperlinks to other resources, etc. [1].

	1	2	3	4	5	
subitem not at all important	○	○	◉	○	○	essential

Clear selection

Does your paper address subitem 5-viii? *

Copy and paste relevant sections from the manuscript (include quotes in quotation marks "like this" to indicate direct quotes from your manuscript), or elaborate on this item by providing additional information not in the ms, or briefly explain why the item is not applicable/relevant for your study

"Tasks were programmed and presented using Inquisit 6.4 (Inquisit Web Millisecond software package) on participants’ internet-connected computers. To account for different screen sizes and ensure consistency in the display of word stimuli across participants, a calibration process was performed at the start of each session, asking participants to place a credit card on the screen and adjust the length of a horizontal line until it matched the width of the credit card.
The 3 training tasks were delivered using modified versions of the dot-probe task [11]."

See published protocol and the manuscript for further information.

5-ix) Describe use parameters

Describe use parameters (e.g., intended “doses” and optimal timing for use). Clarify what instructions or recommendations were given to the user, e.g., regarding timing, frequency, heaviness of use, if any, or was the intervention used ad libitum.

	1	2	3	4	5	
subitem not at all important	○	○	○	◉	○	essential

Clear selection

Does your paper address subitem 5-ix?

Copy and paste relevant sections from the manuscript (include quotes in quotation marks "like this" to indicate direct quotes from your manuscript), or elaborate on this item by providing additional information not in the ms, or briefly explain why the item is not applicable/relevant for your study

"Participants performed the training sessions online at their time and place of convenience, twice a week on a separate pair of days (Monday and Thursday or Tuesday and Friday) for 3 consecutive weeks, totaling 6 training sessions. This dosage was based on previous pain ABMT literature [10,12,16], which has shown positive training effects for dosage ranging between 4 and 8 sessions. After each training session, participants were asked to rate their engagement with the task. The first and final sessions took approximately 30 minutes, as they included cognitive assessment measures (ie, the dot-probe and interpretation bias assessment tasks were administered at the beginning, before training at session 1, and at the end, after the last training block in session 6), whereas sessions 2 to 5 took approximately 15 minutes to complete. Participants were asked to complete the sessions within 24 hours of receiving a web link on a computer with a proper keyboard and to create a quiet and private environment free from distractions for at least 30 minutes. Each session started with the same instructions, similar to those used in previous research (eg, [20]). "

5-x) Clarify the level of human involvement

Clarify the level of human involvement (care providers or health professionals, also technical assistance) in the e-intervention or as co-intervention (detail number and expertise of professionals involved, if any, as well as “type of assistance offered, the timing and frequency of the support, how it is initiated, and the medium by which the assistance is delivered”. It may be necessary to distinguish between the level of human involvement required for the trial, and the level of human involvement required for a routine application outside of a RCT setting (discuss under item 21 – generalizability).

	1	2	3	4	5	
subitem not at all important	○	○	○	◉	○	essential

Clear selection

Does your paper address subitem 5-x?

Copy and paste relevant sections from the manuscript (include quotes in quotation marks "like this" to indicate direct quotes from your manuscript), or elaborate on this item by providing additional information not in the ms, or briefly explain why the item is not applicable/relevant for your study

""All training sessions were conducted via the internet at the participants’ time and place of convenience using their own computers, and all outcome assessments (ie, at baseline, during training, immediately after training, and at 1-month posttraining) were self-assessed via web-based questionnaires and computerized tasks.""
"They could contact the first author (JV) by email or phone if they had any questions or technical problems."

5-xi) Report any prompts/reminders used

Report any prompts/reminders used: Clarify if there were prompts (letters, emails, phone calls, SMS) to use the application, what triggered them, frequency etc. It may be necessary to distinguish between the level of prompts/reminders required for the trial, and the level of prompts/reminders for a routine application outside of a RCT setting (discuss under item 21 – generalizability).

	1	2	3	4	5	
subitem not at all important	○	○	○	◉	○	essential

Clear selection

Does your paper address subitem 5-xi? *

Copy and paste relevant sections from the manuscript (include quotes in quotation marks "like this" to indicate direct quotes from your manuscript), or elaborate on this item by providing additional information not in the ms, or briefly explain why the item is not applicable/relevant for your study

"A combination of SMS text message and email message reminders were sent to those who did not complete the scheduled session within 24 hours of receiving the web link. Participants were allowed to skip training sessions but were excluded from the analyses if they did not successfully complete at least 1 session (ie, minimum threshold exposure to ABMT)." "Participants who failed to complete the follow-up assessment received up to 2 emails or SMS text message reminders."

5-xii) Describe any co-interventions (incl. training/support)

Describe any co-interventions (incl. training/support): Clearly state any interventions that are provided in addition to the targeted eHealth intervention, as ehealth intervention may not be designed as stand-alone intervention. This includes training sessions and support [1]. It may be necessary to distinguish between the level of training required for the trial, and the level of training for a routine application outside of a RCT setting (discuss under item 21 – generalizability.

	1	2	3	4	5	
subitem not at all important	○	○	◉	○	○	essential

Clear selection

Does your paper address subitem 5-xii? *

Copy and paste relevant sections from the manuscript (include quotes in quotation marks "like this" to indicate direct quotes from your manuscript), or elaborate on this item by providing additional information not in the ms, or briefly explain why the item is not applicable/relevant for your study

No other interventions were provided in addition to the targeted intervention. However, "Concomitant care (eg, rehabilitation program and pain medications) was permitted during the trial and was monitored through a pain treatment question that probed participants' pain treatments and frequency since the commencement of the study or previous assessment. " "Concomitant care received during the study was categorized into pain treatment received (yes or no) and use of medication (yes or no). "

6a) Completely defined pre-specified primary and secondary outcome measures, including how and when they were assessed

Does your paper address CONSORT subitem 6a? *

Copy and paste relevant sections from the manuscript (include quotes in quotation marks "like this" to indicate direct quotes from your manuscript), or elaborate on this item by providing additional information not in the ms, or briefly explain why the item is not applicable/relevant for your study

"Primary outcomes included self-reported and behavioral engagement, pain intensity, and pain interference. Secondary outcomes included anxiety, depression, cognitive biases, and perceived improvement." "Data were collected via the internet using the web-based system Qualtrics (Provo) for survey responses and Inquisit 6.4 (Inquisit Web Millisecond software package) for task data and self-reported engagement responses. Participants' outcomes were assessed before beginning training (baseline), during training (for self-reported task-related engagement), immediately after completion of training (posttraining), and 1 month after the last training session (follow-up)."

6a-i) Online questionnaires: describe if they were validated for online use and apply CHERRIES items to describe how the questionnaires were designed/deployed

If outcomes were obtained through online questionnaires, describe if they were validated for online use and apply CHERRIES items to describe how the questionnaires were designed/deployed [9].

	1	2	3	4	5	
subitem not at all important	○	○	●	○	○	essential

Clear selection

Does your paper address subitem 6a-i?

Copy and paste relevant sections from manuscript text

Some of the questionnaires and tasks are validated for online use.

6a-ii) Describe whether and how “use” (including intensity of use/dosage) was defined/measured/monitored

Describe whether and how “use” (including intensity of use/dosage) was defined/measured/monitored (logins, logfile analysis, etc.). Use/adoption metrics are important process outcomes that should be reported in any ehealth trial.

	1	2	3	4	5	
subitem not at all important	○	○	◉	○	○	essential

Clear selection

Does your paper address subitem 6a-ii?

Copy and paste relevant sections from manuscript text

"We monitored attrition throughout the study, and recruitment ended when approximately 120 participants had completed at least 1 training session (ie, minimum threshold for exposure to ABMT)." "The first author (JV) could access the training data to monitor the data collection process and was responsible to respond to participants who had questions or technical issues." "A total of 2 behavioral measures were used to assess participants' engagement: nonuse intervention attrition [58], defined as the number of participants who discontinued using the intervention at each training session, and completion rates, defined as the number of training sessions (out of 6) that each participant completed during the intervention period."

6a-iii) Describe whether, how, and when qualitative feedback from participants was obtained

Describe whether, how, and when qualitative feedback from participants was obtained (e.g., through emails, feedback forms, interviews, focus groups).

	1	2	3	4	5	
subitem not at all important	○	○	◉	○	○	essential

Clear selection

Does your paper address subitem 6a-iii?

Copy and paste relevant sections from manuscript text

Feedback was provided through emails and phone calls.
"For those who provided unsolicited feedback (25/63, 39.7%), the reasons for nonuse intervention attrition were technical issues (8/25, 32%), sick or in hospital (5/25, 20%), unable to tolerate the training sessions (4/25, 16%), lack of interest (4/25, 16%), too busy or away (2/25, 8%), and no access to a computer due to the COVID-19 pandemic lockdown or floods (2/25, 8%)."

6b) Any changes to trial outcomes after the trial commenced, with reasons

Does your paper address CONSORT subitem 6b? *

Copy and paste relevant sections from the manuscript (include quotes in quotation marks "like this" to indicate direct quotes from your manuscript), or elaborate on this item by providing additional information not in the ms, or briefly explain why the item is not applicable/relevant for your study

There were no changes to trial outcomes.

7a) How sample size was determined

NPT: When applicable, details of whether and how the clustering by care provides or centers was addressed

7a-i) Describe whether and how expected attrition was taken into account when calculating the sample size

Describe whether and how expected attrition was taken into account when calculating the sample size.

	1	2	3	4	5	
subitem not at all important	○	○	○	◉	○	essential

Clear selection

Does your paper address subitem 7a-i?

Copy and paste relevant sections from manuscript title (include quotes in quotation marks "like this" to indicate direct quotes from your manuscript), or elaborate on this item by providing additional information not in the ms, or briefly explain why the item is not applicable/relevant for your study

"Considering attrition rates of previous trials in chronic pain treatment [33] and given the 1-month follow-up assessment, a dropout rate of approximately 30% was expected for this trial. Therefore, a total target sample size of 120 participants (40 participants per condition) was sought. We monitored attrition throughout the study, and recruitment ended when approximately 120 participants had completed at least 1 training session (ie, minimum threshold for exposure to ABMT)."

7b) When applicable, explanation of any interim analyses and stopping guidelines

Does your paper address CONSORT subitem 7b? *

Copy and paste relevant sections from the manuscript (include quotes in quotation marks "like this" to indicate direct quotes from your manuscript), or elaborate on this item by providing additional information not in the ms, or briefly explain why the item is not applicable/relevant for your study

"No analyses were performed until recruitment and data collection were completed."

8a) Method used to generate the random allocation sequence

NPT: When applicable, how care providers were allocated to each trial group

Does your paper address CONSORT subitem 8a? *

Copy and paste relevant sections from the manuscript (include quotes in quotation marks "like this" to indicate direct quotes from your manuscript), or elaborate on this item by providing additional information not in the ms, or briefly explain why the item is not applicable/relevant for your study

"Participant randomization was performed by an independent researcher with no involvement in the study using a computerized random number generator, Sealed Envelope (Sealed Envelope Ltd). "

8b) Type of randomisation; details of any restriction (such as blocking and block size)

Does your paper address CONSORT subitem 8b? *

Copy and paste relevant sections from the manuscript (include quotes in quotation marks "like this" to indicate direct quotes from your manuscript), or elaborate on this item by providing additional information not in the ms, or briefly explain why the item is not applicable/relevant for your study

"A block randomization technique was used, allowing 6 participants at a time to be randomized in equal proportions to the 3 training arms."

9) Mechanism used to implement the random allocation sequence (such as sequentially numbered containers), describing any steps taken to conceal the sequence until interventions were assigned

Does your paper address CONSORT subitem 9? *

Copy and paste relevant sections from the manuscript (include quotes in quotation marks "like this" to indicate direct quotes from your manuscript), or elaborate on this item by providing additional information not in the ms, or briefly explain why the item is not applicable/relevant for your study

Manuscript: "After the baseline assessment, eligible consenting adults were randomly allocated to 1 of the 3 training conditions: standard ABMT, gamified ABMT, or control. Participant randomization was performed by an independent researcher with no involvement in the study using a computerized random number generator, Sealed Envelope (Sealed Envelope Ltd). A block randomization technique was used, allowing 6 participants at a time to be randomized in equal proportions to the 3 training arms. "
Published protocol: "A block randomization technique will be used, allowing 6 participants at a time to be randomized in equal proportions to the 3 training arms. The allocation numbers will be stored on a password-protected university database maintained by the same independent person and will be revealed after participants are enrolled and baseline assessments are completed.
Each time a participant completes the baseline survey, an automatic email will be sent to the principal researcher (JFV). After receiving this email, the participant will be allocated to the next number on the list and, consequently, be assigned to a training arm."

10) Who generated the random allocation sequence, who enrolled participants, and who assigned participants to interventions

Does your paper address CONSORT subitem 10? *

Copy and paste relevant sections from the manuscript (include quotes in quotation marks "like this" to indicate direct quotes from your manuscript), or elaborate on this item by providing additional information not in the ms, or briefly explain why the item is not applicable/relevant for your study

Manuscript: "Participant randomization was performed by an independent researcher "
Published protocol: "Each time a participant completes the baseline survey, an automatic email will be sent to the principal researcher (JFV). After receiving this email, the participant will be allocated to the next number on the list and, consequently, be assigned to a training arm."

11a) If done, who was blinded after assignment to interventions (for example, participants, care providers, those assessing outcomes) and how

NPT: Whether or not administering co-interventions were blinded to group assignment

11a-i) Specify who was blinded, and who wasn't

Specify who was blinded, and who wasn't. Usually, in web-based trials it is not possible to blind the participants [1, 3] (this should be clearly acknowledged), but it may be possible to blind outcome assessors, those doing data analysis or those administering co-interventions (if any).

	1	2	3	4	5	
subitem not at all important	○	○	○	●	○	essential

Clear selection

Does your paper address subitem 11a-i? *

Copy and paste relevant sections from the manuscript (include quotes in quotation marks "like this" to indicate direct quotes from your manuscript), or elaborate on this item by providing additional information not in the ms, or briefly explain why the item is not applicable/relevant for your study

"Participants and researchers were both blinded to the training condition to which participants were assigned. Participants were not provided information about the 3 training conditions but were informed that they might receive the intervention or complete a similar task (the control condition). In doing so, participants remained blinded to their allocation, as it would have been easy to realize whether they were in the active gamified condition or not (and noting that the control condition does not include game elements). The outcome data were blinded, as assessments and training occurred on the web in the absence of the investigators. The first author (JV) could access the training data to monitor the data collection process and was responsible to respond to participants who had questions or technical issues. However, it is unlikely that this caused problems of bias allocation or assessment because of the web-based nature of the study."

11a-ii) Discuss e.g., whether participants knew which intervention was the “intervention of interest” and which one was the “comparator”

Informed consent procedures (4a-ii) can create biases and certain expectations - discuss e.g., whether participants knew which intervention was the “intervention of interest” and which one was the “comparator”.

	1	2	3	4	5	
subitem not at all important	○	○	○	◉	○	essential

Clear selection

Does your paper address subitem 11a-ii?

Copy and paste relevant sections from the manuscript (include quotes in quotation marks "like this" to indicate direct quotes from your manuscript), or elaborate on this item by providing additional information not in the ms, or briefly explain why the item is not applicable/relevant for your study

"Participants were not provided information about the 3 training conditions but were informed that they might receive the intervention or complete a similar task (the control condition). In doing so, participants remained blinded to their allocation, as it would have been easy to realize whether they were in the active gamified condition or not (and noting that the control condition does not include game elements)."

11b) If relevant, description of the similarity of interventions
(this item is usually not relevant for ehealth trials as it refers to similarity of a placebo or sham intervention to a active medication/intervention)

Does your paper address CONSORT subitem 11b? *

Copy and paste relevant sections from the manuscript (include quotes in quotation marks "like this" to indicate direct quotes from your manuscript), or elaborate on this item by providing additional information not in the ms, or briefly explain why the item is not applicable/relevant for your study

""Participants were not provided information about the 3 training conditions but were informed that they might receive the intervention or complete a similar task (the control condition). In doing so, participants remained blinded to their allocation, as it would have been easy to realize whether they were in the active gamified condition or not (and noting that the control condition does not include game elements)."

12a) Statistical methods used to compare groups for primary and secondary outcomes
NPT: When applicable, details of whether and how the clustering by care providers or centers was addressed

Does your paper address CONSORT subitem 12a? *

Copy and paste relevant sections from the manuscript (include quotes in quotation marks "like this" to indicate direct quotes from your manuscript), or elaborate on this item by providing additional information not in the ms, or briefly explain why the item is not applicable/relevant for your study

"Significance for all statistical tests were set at P<.05 (2-tailed). Effect sizes were presented by the test's most appropriate effect size [76]. For the linear mixed models, a standardized Cohen d was calculated from the estimated marginal means tables [77]. " "A series of linear mixed model analyses were conducted to examine changes over time in symptoms (ie, pain intensity, pain interference, anxiety, and depression), self-reported task-related engagement, cognitive biases (ie, attentional bias and interpretation bias), and perceived improvement in the different training conditions. The categories baseline, control, married or in a relationship, employed, and session 1 were used as reference categories for time, training condition, marital status, work status, and session, respectively. A backward modeling approach was used to build the most parsimonious model to test the hypotheses [78], using the Akaike information criterion (AIC) and Bayesian information criterion (BIC) to identify the most appropriate model. Specifically, a 3-step model building procedure was conducted to find the best-fit model. First (model 1), we constructed a model that included the main effects and covariates. Second (model 2), we removed from the model covariates that were nonsignificant to see if it improved the fit of the model. Third (model 3), we added the interaction terms to the previously best-fit model (model 1 or model 2) and compared the 3 models on their fit to the data using the AIC and BIC. All models incorporated a random intercept for participants and used the maximum likelihood estimator. Finally, sensitivity analyses were conducted on all primary analyses without controlling for the covariates. Comparisons of models for each primary and secondary outcome variable are available in Multimedia Appendix 3.
To analyze the impact of gamification on interest and enjoyment, a 1-way analysis of covariance in a general linear model was performed (no data were missing). Regarding behavioral engagement, Kaplan-Meier survival curves [79] were calculated to assess the time at which attrition occurred in each training condition and compared statistically using a log-rank test. The number of sessions completed was the time variable, and the event variable was specified as the moment of ceasing participation. Participants were classified as noncompleters if they did not complete all 6 training sessions. In addition, a 1-way analysis of covariance in a general linear model was performed to determine whether there were differences in the mean number of sessions completed between the training conditions. Pearson correlations assessed the relationship between changes in attentional bias magnitude from pre- to posttraining assessment and changes in scores on symptom measures (ie, pain intensity, pain interference, anxiety, and depression). Change in attentional bias was calculated by subtracting the attentional bias score in the pretraining session from the attentional bias score in the posttraining session."

12a-i) Imputation techniques to deal with attrition / missing values

Imputation techniques to deal with attrition / missing values: Not all participants will use the intervention/comparator as intended and attrition is typically high in ehealth trials. Specify how participants who did not use the application or dropped out from the trial were treated in the statistical analysis (a complete case analysis is strongly discouraged, and simple imputation techniques such as LOCF may also be problematic [4]).

	1	2	3	4	5	
subitem not at all important	○	○	○	◉	○	essential

Clear selection

Does your paper address subitem 12a-i? *

Copy and paste relevant sections from the manuscript (include quotes in quotation marks "like this" to indicate direct quotes from your manuscript), or elaborate on this item by providing additional information not in the ms, or briefly explain why the item is not applicable/relevant for your study

"To manage missing data, linear mixed model analyses were used (where appropriate), as it allows the inclusion of all available data. Missing PROMIS measures data were dealt with according to recommendations in the scoring manual by using the HealthMeasures Scoring Service [59]."

12b) Methods for additional analyses, such as subgroup analyses and adjusted analyses

Does your paper address CONSORT subitem 12b? *

Copy and paste relevant sections from the manuscript (include quotes in quotation marks "like this" to indicate direct quotes from your manuscript), or elaborate on this item by providing additional information not in the ms, or briefly explain why the item is not applicable/relevant for your study

"Finally, sensitivity analyses were conducted on all primary analyses without controlling for the covariates. Comparisons of models for each primary and secondary outcome variable are available in Multimedia Appendix 3."
"Finally, exploratory analyses were conducted to address additional questions. These included the role of engagement metrics (ie, number of training sessions completed) and individual differences (ie, attentional control, pain-related worrying, personality characteristics, and recruitment setting) in the impact of training conditions on pain intensity and pain interference. Models for each exploratory analysis are available in Multimedia Appendix 2."

X26) REB/IRB Approval and Ethical Considerations [recommended as subheading under "Methods"] (not a CONSORT item)

X26-i) Comment on ethics committee approval

	1	2	3	4	5	
subitem not at all important	○	○	○	○	◉	essential

Clear selection

Does your paper address subitem X26-i?

Copy and paste relevant sections from the manuscript (include quotes in quotation marks "like this" to indicate direct quotes from your manuscript), or elaborate on this item by providing additional information not in the ms, or briefly explain why the item is not applicable/relevant for your study

"The study was approved by the Human Research Ethics Committees of the Royal Brisbane and Women's Hospital (HREC/2020/QRBW/61743) and Queensland University of Technology (2000000395) and prospectively registered on the Australian New Zealand Clinical Trials Registry (ACTRN12620000803998)."

x26-ii) Outline informed consent procedures

Outline informed consent procedures e.g., if consent was obtained offline or online (how? Checkbox, etc.?), and what information was provided (see 4a-ii). See [6] for some items to be included in informed consent documents.

	1	2	3	4	5	
subitem not at all important	○	○	○	○	◉	essential

Clear selection

Does your paper address subitem X26-ii?

Copy and paste relevant sections from the manuscript (include quotes in quotation marks "like this" to indicate direct quotes from your manuscript), or elaborate on this item by providing additional information not in the ms, or briefly explain why the item is not applicable/relevant for your study

"Electronic informed consent (ie, e-consent) was obtained for all study participants."

X26-iii) Safety and security procedures

Safety and security procedures, incl. privacy considerations, and any steps taken to reduce the likelihood or detection of harm (e.g., education and training, availability of a hotline)

	1	2	3	4	5	
subitem not at all important	○	○	○	◉	○	essential

Clear selection

Does your paper address subitem X26-iii?

Copy and paste relevant sections from the manuscript (include quotes in quotation marks "like this" to indicate direct quotes from your manuscript), or elaborate on this item by providing additional information not in the ms, or briefly explain why the item is not applicable/relevant for your study

Published protocol: "Owing to the minimal risks associated with study participation, it is not necessary to implement an independent data and safety monitoring board. The research team will be responsible for monitoring and data management and will meet regularly to manage the protocol, monitor recruitment, and deal with any adverse events.

Data safety and security measures have been considered, including restricted access to the research team, password protection, firewall, and virus protection. Furthermore, each participant will be assigned a unique participant code (blinded to the group the participant has been assigned to) and will be asked to self-generate an ID code (instead of participants' personal information). Thus, this coded data may be reidentifiable during the research but will be deidentified upon completion of the study. An electronic, password-locked master file will be created that matches the unique participant code to their self-generated code to ensure participants are allocated to the correct experimental group and that the survey and task data match across the different time points. All data will be stored on a secure password-protected university server and accessed only by the research team.

On the basis of previous similar trials, no adverse events are expected [10]. A small amount of fatigue and some mild discomfort during the training task may be experienced. This will be managed by providing participants with enough rest periods between blocks of trials. If the research team becomes aware of any harm or other adverse events, it will be documented and reported appropriately. The research team will also manage any risks and recommend participants to liaise with relevant services, such as psychological assistance, if appropriate. The study will be stopped if evidence emerges that participants can come to harm because of the ABMT intervention."

RESULTS

13a) For each group, the numbers of participants who were randomly assigned, received intended treatment, and were analysed for the primary outcome

NPT: The number of care providers or centers performing the intervention in each group and the number of patients treated by each care provider in each center

Does your paper address CONSORT subitem 13a? *

Copy and paste relevant sections from the manuscript (include quotes in quotation marks "like this" to indicate direct quotes from your manuscript), or elaborate on this item by providing additional information not in the ms, or briefly explain why the item is not applicable/relevant for your study

"Of the 206 participants who were randomized, 73 (35.4%) were excluded from all analyses due to not successfully completing at least 1 training session, which was the minimum threshold exposure to ABMT (n=25, 12.1% standard ABMT; n=25, 12.1% gamified ABMT; and n=23, 11.2% control), 3 (1.5%) were excluded for answering all the instructional questions incorrectly (n=1, 0.5% standard ABMT and n=2, 1% gamified ABMT), and 1 (0.5%) was excluded for having only chronic neuropathic pain (gamified ABMT). The final sample size included in the analysis was 129 adults with chronic musculoskeletal pain (n=43, 33.3% standard ABMT; n=41, 31.8% gamified ABMT; and n=45, 34.9% control)."

13b) For each group, losses and exclusions after randomisation, together with reasons

Does your paper address CONSORT subitem 13b? (NOTE: Preferably, this is shown in a CONSORT flow diagram) *

Copy and paste relevant sections from the manuscript (include quotes in quotation marks "like this" to indicate direct quotes from your manuscript), or elaborate on this item by providing additional information not in the ms, or briefly explain why the item is not applicable/relevant for your study

"Of the 206 participants who were randomized, 73 (35.4%) were excluded from all analyses due to not successfully completing at least 1 training session, which was the minimum threshold exposure to ABMT (n=25, 12.1% standard ABMT; n=25, 12.1% gamified ABMT; and n=23, 11.2% control), 3 (1.5%) were excluded for answering all the instructional questions incorrectly (n=1, 0.5% standard ABMT and n=2, 1% gamified ABMT), and 1 (0.5%) was excluded for having only chronic neuropathic pain (gamified ABMT). The final sample size included in the analysis was 129 adults with chronic musculoskeletal pain (n=43, 33.3% standard ABMT; n=41, 31.8% gamified ABMT; and n=45, 34.9% control)."

13b-i) Attrition diagram

Strongly recommended: An attrition diagram (e.g., proportion of participants still logging in or using the intervention/comparator in each group plotted over time, similar to a survival curve) or other figures or tables demonstrating usage/dose/engagement.

	1	2	3	4	5	
subitem not at all important	○	○	○	○	◉	essential

Clear selection

Does your paper address subitem 13b-i?

Copy and paste relevant sections from the manuscript or cite the figure number if applicable (include quotes in quotation marks "like this" to indicate direct quotes from your manuscript), or elaborate on this item by providing additional information not in the ms, or briefly explain why the item is not applicable/relevant for your study

"Figure 2 shows the CONSORT flow diagram for the study."

14a) Dates defining the periods of recruitment and follow-up

Does your paper address CONSORT subitem 14a? *

Copy and paste relevant sections from the manuscript (include quotes in quotation marks "like this" to indicate direct quotes from your manuscript), or elaborate on this item by providing additional information not in the ms, or briefly explain why the item is not applicable/relevant for your study

"The first participant for this study was enrolled in August 2021, and the final follow-up assessment was completed in June 2022."

14a-i) Indicate if critical “secular events” fell into the study period

Indicate if critical “secular events” fell into the study period, e.g., significant changes in Internet resources available or “changes in computer hardware or Internet delivery resources”

	1	2	3	4	5	
subitem not at all important	○	◉	○	○	○	essential

Clear selection

Does your paper address subitem 14a-i?

Copy and paste relevant sections from the manuscript (include quotes in quotation marks "like this" to indicate direct quotes from your manuscript), or elaborate on this item by providing additional information not in the ms, or briefly explain why the item is not applicable/relevant for your study

"For those who provided unsolicited feedback (25/63, 39.7%), the reasons for nonuse intervention attrition were technical issues (8/25, 32%), sick or in hospital (5/25, 20%), unable to tolerate the training sessions (4/25, 16%), lack of interest (4/25, 16%), too busy or away (2/25, 8%), and no access to a computer due to the COVID-19 pandemic lockdown or floods (2/25, 8%)."

14b) Why the trial ended or was stopped (early)

Does your paper address CONSORT subitem 14b? *

Copy and paste relevant sections from the manuscript (include quotes in quotation marks "like this" to indicate direct quotes from your manuscript), or elaborate on this item by providing additional information not in the ms, or briefly explain why the item is not applicable/relevant for your study

"We monitored attrition throughout the study, and recruitment ended when approximately 120 participants had completed at least 1 training session (ie, minimum threshold for exposure to ABMT)."

15) A table showing baseline demographic and clinical characteristics for each group

NPT: When applicable, a description of care providers (case volume, qualification, expertise, etc.) and centers (volume) in each group

Does your paper address CONSORT subitem 15? *

Copy and paste relevant sections from the manuscript (include quotes in quotation marks "like this" to indicate direct quotes from your manuscript), or elaborate on this item by providing additional information not in the ms, or briefly explain why the item is not applicable/relevant for your study

"Table 2. Baseline key characteristics of the sample by training condition (N=129)."

15-i) Report demographics associated with digital divide issues

In ehealth trials it is particularly important to report demographics associated with digital divide issues, such as age, education, gender, social-economic status, computer/Internet/ehealth literacy of the participants, if known.

	1	2	3	4	5	
subitem not at all important	○	○	◉	○	○	essential

Clear selection

Does your paper address subitem 15-i? *

Copy and paste relevant sections from the manuscript (include quotes in quotation marks "like this" to indicate direct quotes from your manuscript), or elaborate on this item by providing additional information not in the ms, or briefly explain why the item is not applicable/relevant for your study

"Table 3. Summary statistics on outcome measures by training condition and assessment time points."

16) For each group, number of participants (denominator) included in each analysis and whether the analysis was by original assigned groups

16-i) Report multiple “denominators” and provide definitions

Report multiple “denominators” and provide definitions: Report N’s (and effect sizes) “across a range of study participation [and use] thresholds” [1], e.g., N exposed, N consented, N used more than x times, N used more than y weeks, N participants “used” the intervention/comparator at specific pre-defined time points of interest (in absolute and relative numbers per group). Always clearly define “use” of the intervention.

	1	2	3	4	5	
subitem not at all important	○	○	○	◉	○	essential

Clear selection

Does your paper address subitem 16-i? *

Copy and paste relevant sections from the manuscript (include quotes in quotation marks "like this" to indicate direct quotes from your manuscript), or elaborate on this item by providing additional information not in the ms, or briefly explain why the item is not applicable/relevant for your study

"Table 3. Summary statistics on outcome measures by training condition and assessment time points." See Multimedia Appendices 2 and 3

16-ii) Primary analysis should be intent-to-treat

Primary analysis should be intent-to-treat, secondary analyses could include comparing only “users”, with the appropriate caveats that this is no longer a randomized sample (see 18-i).

	1	2	3	4	5	
subitem not at all important	○	○	◉	○	○	essential

Clear selection

Does your paper address subitem 16-ii?

Copy and paste relevant sections from the manuscript (include quotes in quotation marks "like this" to indicate direct quotes from your manuscript), or elaborate on this item by providing additional information not in the ms, or briefly explain why the item is not applicable/relevant for your study

"Analyses included all randomized participants who successfully completed at least 1 training session."

17a) For each primary and secondary outcome, results for each group, and the estimated effect size and its precision (such as 95% confidence interval)

Does your paper address CONSORT subitem 17a? *

Copy and paste relevant sections from the manuscript (include quotes in quotation marks "like this" to indicate direct quotes from your manuscript), or elaborate on this item by providing additional information not in the ms, or briefly explain why the item is not applicable/relevant for your study

See Tables 4 and 5 as well as Multimedia Appendices 2 and 3.

17a-i) Presentation of process outcomes such as metrics of use and intensity of use

In addition to primary/secondary (clinical) outcomes, the presentation of process outcomes such as metrics of use and intensity of use (dose, exposure) and their operational definitions is critical. This does not only refer to metrics of attrition (13-b) (often a binary variable), but also to more continuous exposure metrics such as “average session length”. These must be accompanied by a technical description how a metric like a “session” is defined (e.g., timeout after idle time) [1] (report under item 6a).

	1	2	3	4	5	
subitem not at all important	○	○	○	◉	○	essential

Clear selection

Does your paper address subitem 17a-i?

Copy and paste relevant sections from the manuscript (include quotes in quotation marks "like this" to indicate direct quotes from your manuscript), or elaborate on this item by providing additional information not in the ms, or briefly explain why the item is not applicable/relevant for your study

"On average, participants completed 4.60 (SD 1.83) out of 6 training sessions, which did not differ significantly between conditions ($F_{2,118}=1.24$; $P=.29$; $\eta^2_p=0.021$). The attrition pattern of the 3-week intervention period is presented in the Kaplan-Meier plot in Figure 3. Nonuse intervention attrition occurred for 58.1% (25/43), 43.9% (18/41), and 44.4% (20/45) of the standard ABMT, gamified ABMT, and control conditions, respectively, which is comparable to other web-based health interventions [80-83]. Dropout occurred throughout the course of the intervention, with no significant differences between conditions in the time of attrition (log-rank $\chi^2_2=2.8$; $P=.25$)."
"A total of 2 behavioral measures were used to assess participants' engagement: nonuse intervention attrition [58], defined as the number of participants who discontinued using the intervention at each training session, and completion rates, defined as the number of training sessions (out of 6) that each participant completed during the intervention period."

17b) For binary outcomes, presentation of both absolute and relative effect sizes is recommended

Does your paper address CONSORT subitem 17b? *

Copy and paste relevant sections from the manuscript (include quotes in quotation marks "like this" to indicate direct quotes from your manuscript), or elaborate on this item by providing additional information not in the ms, or briefly explain why the item is not applicable/relevant for your study

Please see question 17 a.

18) Results of any other analyses performed, including subgroup analyses and adjusted analyses, distinguishing pre-specified from exploratory

Does your paper address CONSORT subitem 18? *

Copy and paste relevant sections from the manuscript (include quotes in quotation marks "like this" to indicate direct quotes from your manuscript), or elaborate on this item by providing additional information not in the ms, or briefly explain why the item is not applicable/relevant for your study

See Tables 4 and 5 as well as Multimedia Appendices 2 and 3.

18-i) Subgroup analysis of comparing only users

A subgroup analysis of comparing only users is not uncommon in ehealth trials, but if done, it must be stressed that this is a self-selected sample and no longer an unbiased sample from a randomized trial (see 16-iii).

	1	2	3	4	5	
subitem not at all important	○	○	○	○	○	essential

Does your paper address subitem 18-i?

Copy and paste relevant sections from the manuscript (include quotes in quotation marks "like this" to indicate direct quotes from your manuscript), or elaborate on this item by providing additional information not in the ms, or briefly explain why the item is not applicable/relevant for your study

Your answer

19) All important harms or unintended effects in each group

(for specific guidance see CONSORT for harms)

Does your paper address CONSORT subitem 19? *

Copy and paste relevant sections from the manuscript (include quotes in quotation marks "like this" to indicate direct quotes from your manuscript), or elaborate on this item by providing additional information not in the ms, or briefly explain why the item is not applicable/relevant for your study

"Similar to previous studies (eg, [20]), no serious adverse events were reported. However, 5 participants (3/41, 7.3% gamified ABMT; 1/43, 2.3% standard ABMT; and 1/45, 2.2% control) discontinued their participation due to training-related nonserious adverse events. Specifically, despite being offered a rest between each block of trials, 4 participants reported that the physical action required to complete the tasks (ie, repetitive movements and sitting for prolonged periods) triggered pain in their hand, wrist, back, or neck, and 1 participant reported that the task caused dizziness. Of these 5 participants, 2 (2/129, 1.6%) specified that their difficulty in performing the tasks was aggravated by the COVID-19 pandemic lockdowns, which prevented them from receiving regular treatment for their pain. Another participant in the standard ABMT condition discontinued participation due to fear that the thin black writing on a white background might be a migraine trigger and suggested offering the option of a dark mode (ie, white or gray text presented against a dark or back background) and large text (ie, bolding and increasing the font size)."

19-i) Include privacy breaches, technical problems

Include privacy breaches, technical problems. This does not only include physical “harm” to participants, but also incidents such as perceived or real privacy breaches [1], technical problems, and other unexpected/unintended incidents. “Unintended effects” also includes unintended positive effects [2].

	1	2	3	4	5	
subitem not at all important	○	○	○	◉	○	essential

Clear selection

Does your paper address subitem 19-i?

Copy and paste relevant sections from the manuscript (include quotes in quotation marks "like this" to indicate direct quotes from your manuscript), or elaborate on this item by providing additional information not in the ms, or briefly explain why the item is not applicable/relevant for your study

"Similar to previous studies (eg, [20]), no serious adverse events were reported. However, 5 participants (3/41, 7.3% gamified ABMT; 1/43, 2.3% standard ABMT; and 1/45, 2.2% control) discontinued their participation due to training-related nonserious adverse events. Specifically, despite being offered a rest between each block of trials, 4 participants reported that the physical action required to complete the tasks (ie, repetitive movements and sitting for prolonged periods) triggered pain in their hand, wrist, back, or neck, and 1 participant reported that the task caused dizziness. Of these 5 participants, 2 (2/129, 1.6%) specified that their difficulty in performing the tasks was aggravated by the COVID-19 pandemic lockdowns, which prevented them from receiving regular treatment for their pain. Another participant in the standard ABMT condition discontinued participation due to fear that the thin black writing on a white background might be a migraine trigger and suggested offering the option of a dark mode (ie, white or gray text presented against a dark or back background) and large text (ie, bolding and increasing the font size)."

19-ii) Include qualitative feedback from participants or observations from staff/researchers

Include qualitative feedback from participants or observations from staff/researchers, if available, on strengths and shortcomings of the application, especially if they point to unintended/unexpected effects or uses. This includes (if available) reasons for why people did or did not use the application as intended by the developers.

	1	2	3	4	5	
subitem not at all important	○	○	◉	○	○	essential

Clear selection

Does your paper address subitem 19-ii?

Copy and paste relevant sections from the manuscript (include quotes in quotation marks "like this" to indicate direct quotes from your manuscript), or elaborate on this item by providing additional information not in the ms, or briefly explain why the item is not applicable/relevant for your study

"Similar to previous studies (eg, [20]), no serious adverse events were reported. However, 5 participants (3/41, 7.3% gamified ABMT; 1/43, 2.3% standard ABMT; and 1/45, 2.2% control) discontinued their participation due to training-related nonserious adverse events. Specifically, despite being offered a rest between each block of trials, 4 participants reported that the physical action required to complete the tasks (ie, repetitive movements and sitting for prolonged periods) triggered pain in their hand, wrist, back, or neck, and 1 participant reported that the task caused dizziness. Of these 5 participants, 2 (2/129, 1.6%) specified that their difficulty in performing the tasks was aggravated by the COVID-19 pandemic lockdowns, which prevented them from receiving regular treatment for their pain. Another participant in the standard ABMT condition discontinued participation due to fear that the thin black writing on a white background might be a migraine trigger and suggested offering the option of a dark mode (ie, white or gray text presented against a dark or back background) and large text (ie, bolding and increasing the font size)."

DISCUSSION

22) Interpretation consistent with results, balancing benefits and harms, and considering other relevant evidence

NPT: In addition, take into account the choice of the comparator, lack of or partial blinding, and unequal expertise of care providers or centers in each group

22-i) Restate study questions and summarize the answers suggested by the data, starting with primary outcomes and process outcomes (use)

Restate study questions and summarize the answers suggested by the data, starting with primary outcomes and process outcomes (use).

	1	2	3	4	5	
subitem not at all important	○	○	○	○	◉	essential

Clear selection

Does your paper address subitem 22-i? *

Copy and paste relevant sections from the manuscript (include quotes in quotation marks "like this" to indicate direct quotes from your manuscript), or elaborate on this item by providing additional information not in the ms, or briefly explain why the item is not applicable/relevant for your study

"This study investigated the effects of a gamified web-delivered pain ABMT intervention in adults with chronic musculoskeletal pain. The findings can be readily summarized. First, there was no evidence that gamified ABMT was effective in enhancing engagement over and above the standard ABMT and sham training control conditions. Second, there were also no differential effects over time between the active ABMT and control conditions on measures of pain intensity, pain interference, depression, anxiety, or perceived improvement. Third, depressive symptoms, pain intensity, and pain interference reduced over time across all conditions. Finally, there was no indication for the presence of pretraining attentional biases in any of the conditions and no evidence that cognitive biases changed after training for the active ABMT conditions compared to the control condition."

22-ii) Highlight unanswered new questions, suggest future research

Highlight unanswered new questions, suggest future research.

	1	2	3	4	5	
subitem not at all important	○	○	○	○	◉	essential

Clear selection

Does your paper address subitem 22-ii?

Copy and paste relevant sections from the manuscript (include quotes in quotation marks "like this" to indicate direct quotes from your manuscript), or elaborate on this item by providing additional information not in the ms, or briefly explain why the item is not applicable/relevant for your study

"This study has several limitations. First, our sample was restricted to individuals with chronic musculoskeletal pain, thereby limiting the generalizability of findings to other types of pain conditions. Second, despite a rigorous randomization procedure, the conditions differed on several baseline characteristics. Although these were controlled for in our analyses, replications with matched samples are warranted as there might still be some residual confounding. Third, the use of a web-based delivery mode provided limited control over the environment in which ABMT was completed. Given there are suggestions in the anxiety ABMT literature that the efficacy of ABMT may be more difficult to achieve with remote training delivery compared to training in the laboratory [101], future research may want to replicate our findings in a more controlled setting. Fourth, although we opted for the dot-probe assessment paradigm as it is frequently used in web-based attentional bias research [17], we did not assess its reliability, which has been called into question [102]. Future studies may wish to use alternative measures of attentional bias such as eye-tracking technology. Fifth, the selection of a limited number of stimuli (8 stimulus pairs) may have affected the results. Despite our stimulus material consisting of word stimuli that have a strong association to pain, it is possible that a habituation effect to the words reduced their meaningfulness or that it contributed to the decrease in engagement over time. Future research may consider the use of more diverse stimulus types (eg, pictures as well). Finally, we chose to not include a gamified sham (control) condition to maximize power, which limited our ability to determine the impact of cognitive load on the processes underlying ABMT. Future research may consider using a “neutral” or no-contingency control condition such as sham-n training to ascertain whether attentional bias is affected or if cognitive flexibility is trained [90,103]. Future studies would also benefit from using a more specific gamification framework (eg, [104]) that involves an interdisciplinary team of individuals with chronic pain, cognitive experts, and gamification designers to guide the co-design of their interventions."

20) Trial limitations, addressing sources of potential bias, imprecision, and, if relevant, multiplicity of analyses

20-i) Typical limitations in ehealth trials

Typical limitations in ehealth trials: Participants in ehealth trials are rarely blinded. Ehealth trials often look at a multiplicity of outcomes, increasing risk for a Type I error. Discuss biases due to non-use of the intervention/usability issues, biases through informed consent procedures, unexpected events.

	1	2	3	4	5	
subitem not at all important	○	○	○	○	◉	essential

Clear selection

Does your paper address subitem 20-i? *

Copy and paste relevant sections from the manuscript (include quotes in quotation marks "like this" to indicate direct quotes from your manuscript), or elaborate on this item by providing additional information not in the ms, or briefly explain why the item is not applicable/relevant for your study

"This study has several limitations. First, our sample was restricted to individuals with chronic musculoskeletal pain, thereby limiting the generalizability of findings to other types of pain conditions. Second, despite a rigorous randomization procedure, the conditions differed on several baseline characteristics. Although these were controlled for in our analyses, replications with matched samples are warranted as there might still be some residual confounding. Third, the use of a web-based delivery mode provided limited control over the environment in which ABMT was completed. Given there are suggestions in the anxiety ABMT literature that the efficacy of ABMT may be more difficult to achieve with remote training delivery compared to training in the laboratory [101], future research may want to replicate our findings in a more controlled setting. Fourth, although we opted for the dot-probe assessment paradigm as it is frequently used in web-based attentional bias research [17], we did not assess its reliability, which has been called into question [102]. Future studies may wish to use alternative measures of attentional bias such as eye-tracking technology. Fifth, the selection of a limited number of stimuli (8 stimulus pairs) may have affected the results. Despite our stimulus material consisting of word stimuli that have a strong association to pain, it is possible that a habituation effect to the words reduced their meaningfulness or that it contributed to the decrease in engagement over time. Future research may consider the use of more diverse stimulus types (eg, pictures as well). Finally, we chose to not include a gamified sham (control) condition to maximize power, which limited our ability to determine the impact of cognitive load on the processes underlying ABMT. Future research may consider using a “neutral” or no-contingency control condition such as sham-n training to ascertain whether attentional bias is affected or if cognitive flexibility is trained [90,103]. Future studies would also benefit from using a more specific gamification framework (eg, [104]) that involves an interdisciplinary team of individuals with chronic pain, cognitive experts, and gamification designers to guide the co-design of their interventions."

21) Generalisability (external validity, applicability) of the trial findings

NPT: External validity of the trial findings according to the intervention, comparators, patients, and care providers or centers involved in the trial

21-i) Generalizability to other populations

Generalizability to other populations: In particular, discuss generalizability to a general Internet population, outside of a RCT setting, and general patient population, including applicability of the study results for other organizations

	1	2	3	4	5	
subitem not at all important	○	○	○	◉	○	essential

Clear selection

Does your paper address subitem 21-i?

Copy and paste relevant sections from the manuscript (include quotes in quotation marks "like this" to indicate direct quotes from your manuscript), or elaborate on this item by providing additional information not in the ms, or briefly explain why the item is not applicable/relevant for your study

"First, our sample was restricted to individuals with chronic musculoskeletal pain, thereby limiting the generalizability of findings to other types of pain conditions."

21-ii) Discuss if there were elements in the RCT that would be different in a routine application setting

Discuss if there were elements in the RCT that would be different in a routine application setting (e.g., prompts/reminders, more human involvement, training sessions or other co-interventions) and what impact the omission of these elements could have on use, adoption, or outcomes if the intervention is applied outside of a RCT setting.

	1	2	3	4	5	
subitem not at all important	○	○	◉	○	○	essential

Clear selection

Does your paper address subitem 21-ii?

Copy and paste relevant sections from the manuscript (include quotes in quotation marks "like this" to indicate direct quotes from your manuscript), or elaborate on this item by providing additional information not in the ms, or briefly explain why the item is not applicable/relevant for your study

"Third, the use of a web-based delivery mode provided limited control over the environment in which ABMT was completed. Given there are suggestions in the anxiety ABMT literature that the efficacy of ABMT may be more difficult to achieve with remote training delivery compared to training in the laboratory [101], future research may want to replicate our findings in a more controlled setting. "

OTHER INFORMATION

23) Registration number and name of trial registry

Does your paper address CONSORT subitem 23? *

Copy and paste relevant sections from the manuscript (include quotes in quotation marks "like this" to indicate direct quotes from your manuscript), or elaborate on this item by providing additional information not in the ms, or briefly explain why the item is not applicable/relevant for your study

Australian New Zealand Clinical Trials Registry (ANZCTR) ACTRN12620000803998; https://anzctr.org.au/ACTRN12620000803998.aspx

24) Where the full trial protocol can be accessed, if available

Does your paper address CONSORT subitem 24? *

Cite a Multimedia Appendix, other reference, or copy and paste relevant sections from the manuscript (include quotes in quotation marks "like this" to indicate direct quotes from your manuscript), or elaborate on this item by providing additional information not in the ms, or briefly explain why the item is not applicable/relevant for your study

Vermeir JF, White MJ, Johnson D, Crombez G, Van Ryckeghem DM. Gamified web-delivered attentional bias modification training for adults with chronic pain: Protocol for a randomized, double-blind, placebo-controlled trial. JMIR Res Protoc. 2022;11(1):e32359.

25) Sources of funding and other support (such as supply of drugs), role of funders

Does your paper address CONSORT subitem 25? *

Copy and paste relevant sections from the manuscript (include quotes in quotation marks "like this" to indicate direct quotes from your manuscript), or elaborate on this item by providing additional information not in the ms, or briefly explain why the item is not applicable/relevant for your study

"JV received an Australian Government Research Training Program Scholarship and a Queensland University of Technology Excellence Top-Up Scholarship. DV is supported by the Luxembourg National Research Fund (FNR) Core Junior programme (Painflex: Nr. 12671141)."

X27) Conflicts of Interest (not a CONSORT item)

X27-i) State the relation of the study team towards the system being evaluated

In addition to the usual declaration of interests (financial or otherwise), also state the relation of the study team towards the system being evaluated, i.e., state if the authors/evaluators are distinct from or identical with the developers/sponsors of the intervention.

	1	2	3	4	5	
subitem not at all important	○	○	○	●	○	essential

Clear selection

Does your paper address subitem X27-i?

Copy and paste relevant sections from the manuscript (include quotes in quotation marks "like this" to indicate direct quotes from your manuscript), or elaborate on this item by providing additional information not in the ms, or briefly explain why the item is not applicable/relevant for your study

There was no conflict of interest in this study

About the CONSORT EHEALTH checklist

As a result of using this checklist, did you make changes in your manuscript? *

- ○ yes, major changes
- ○ yes, minor changes
- ◉ no

What were the most important changes you made as a result of using this checklist?

Your answer

How much time did you spend on going through the checklist INCLUDING making changes in your manuscript *

It took approximately 4 hours.

As a result of using this checklist, do you think your manuscript has improved? *

- ( ) yes
- (•) no
- ( ) Other:

Would you like to become involved in the CONSORT EHEALTH group?

This would involve for example becoming involved in participating in a workshop and writing an "Explanation and Elaboration" document

- ( ) yes
- (•) no
- ( ) Other:

Clear selection

Any other comments or questions on CONSORT EHEALTH

Your answer

STOP - Save this form as PDF before you click submit

To generate a record that you filled in this form, we recommend to generate a PDF of this page (on a Mac, simply select "print" and then select "print as PDF") before you submit it.

When you submit your (revised) paper to JMIR, please upload the PDF as supplementary file.

Don't worry if some text in the textboxes is cut off, as we still have the complete information in our database. Thank you!

Final step: Click submit !

Click submit so we have your answers in our database!

Submit Clear form

Never submit passwords through Google Forms.

This content is neither created nor endorsed by Google. - Terms of Service - Privacy Policy

Does this form look suspicious? Report

Google Forms
