# Supplementary material for: Gamified Web-Delivered Attentional Bias Modification Training for Adults With Chronic Pain: Randomized, Double-Blind, Placebo-Controlled Trial
Source: JMIR Serious Games. 2025 Jan 16;13:e50635. doi: 10.2196/50635 (PMC11783034; doi:10.2196/50635)
Supplement: Multimedia Appendix 2 [file games_v13i1e50635_app2.pdf]

Multimedia Appendix 2: Additional and Exploratory Analyses

Gamified Web-Delivered Attentional Bias Modification Training for Adults with Chronic Pain: Randomized, Double-Blind, Placebo-Controlled Trial

Julie F Vermeir[1], PhD; Melanie J White[1], PhD; Daniel Johnson[2], PhD; Geert Crombez[3], PhD; Dimitri M L Van Ryckeghem[3,4,5], PhD

[1]School of Psychology and Counselling, Faculty of Health, Queensland University of Technology (QUT), Brisbane, Australia

[2]School of Computer Science, Faculty of Science, Queensland University of Technology (QUT), Brisbane, Australia

[3]Department of Experimental Clinical and Health Psychology, Ghent University, Ghent, Belgium

[4]Department of Clinical Psychological Science, Maastricht University, Maastricht, Netherlands

[5]Department of Behavioural and Cognitive Sciences, University of Luxembourg, Esch-sur-Alzette, Luxembourg

***Corresponding author:** Julie F Vermeir. *Address*: Faculty of Health, School of Psychology and Counselling, Queensland University of Technology (QUT), 170 Victoria Park Road, Brisbane, QLD, 4059, Australia. *Phone*: +61 731384714. *Email address*: julie.vermeir@hdr.qut.edu.au

Trial Registration: Australian New Zealand Clinical Trials Registry ACTRN12620000803998; https://anzctr.org.au/ACTRN12620000803998.aspx

International Registered Report Identifier (IRRID): PRR1-10.2196/32359

1. EXPLORATORY MEASURES

1.1 Measure of Attentional Control

The Attentional Control Scale [1] was used to measure individual differences in attentional control (focusing and shifting attention). The 20-item self-report questionnaire was adapted by including a 7-day time frame for the items. Participants rated the degree to which they endorsed each statement on a 4-point Likert scale from 1 (*almost never*) to 4 (*always*). A total score is calculated, with higher scores indicating a better ability to direct and maintain attention. The Attentional Control Scale has been found to have good reliability [2] and good concurrent validity [1].

1.2 Personality Characteristics

The Behavioral Inhibition System (BIS) and Behavioral Activation System (BAS) scales [3] were used to measure personality traits. This 20-item self-report questionnaire measures trait sensitivity levels of the BIS (punishment; 7 items) and BAS (reward; 13 items) and all items are scored on a 4-point Likert scale ranging from 1 (*strongly disagree*) to 4 (*strongly agree*). The BIS and BAS scales have demonstrated acceptable to good internal consistency [4], and acceptable test-retest reliability over a period of 2 months [3].

1.3 Pain-Related Worrying

The Pain Catastrophizing Scale [5] was used to assess participants' pain-related worrying [6]. The Pain Catastrophizing Scale is a 13-item self-report measure that evaluates 3 subscales: rumination, magnification, and helplessness. Using a 5-point Likert scale, ranging from 0 (*not at all*) to 4 (*all the time*), participants are asked to recall past painful experiences and indicate the extent to which 13 thoughts or feelings are associated with these experiences. The 3 subscale scores are summed to provide a total score for pain-related worrying, ranging between 0 and 52. The Pain Catastrophizing Scale has been found to have good validity and reliability for individuals with chronic pain [5,7].

2. DATA ANALYSIS

Exploratory analyses were conducted to address additional questions. To assess changes over time in attentional control in the different training conditions, linear mixed model analyses were conducted using the same model-building procedure as described in the manuscript. This was a deviation from the protocol as we inadvertently omitted to include this analysis in the *Statistical Methods* section of the protocol [8]. To explore the role of engagement metrics (ie, number of training sessions completed) and individual differences (ie, attentional control, pain-related worrying, personality characteristics, and recruitment setting) in the impact of training conditions on pain intensity and pain interference, a series of linear mixed model analyses were conducted. In the model, the variable of interest and its interaction with training condition was added to the original best fit model to investigate whether participants' pain intensity or pain interference ratings between the training conditions was moderated by the variable of interest. All models incorporated a random intercept for participants and used the maximum likelihood estimator.

3. EXPLORATORY ANALYSES FOR ATTENTIONAL CONTROL

Table S1. Linear Mixed Models for Attentional Control

	**Model 1: Main effects with covariates**					**Model 2: Removing nonsignificant covariate(s)**						**Model 3: Adding interaction terms to the best fit model**				
	B	SE	95% CI		*P*	B	SE	95% CI		*P*	*Cohen's d*	B	SE	95% CI		*P*
Intercept	74.09	4.84	64.51	83.67	<.001*	73.62	4.93	63.86	83.37	<.001*		73.50	4.95	63.72	83.29	<.001*
Posttraining	-0.14	0.63	-1.38	1.10	.82	-0.15	0.63	-1.39	1.10	.81	-.017	0.08	1.07	-2.03	2.19	.94
Gamified ABMT	-0.20	2.02	-4.19	3.80	.92	-1.35	1.84	-4.99	2.28	.46	-.154	-1.25	1.94	-5.08	2.59	.52
Standard ABMT	-1.23	1.84	-4.88	2.41	.51	-0.75	1.79	-4.30	2.80	.68	-.085	-0.58	1.89	-4.32	3.17	.76
Primary[a]	-15.16	8.09	-31.18	0.86	.063	-	-	-	-	-		-	-	-	-	-
Secondary[a]	-1.94	1.89	-5.69	1.80	.31	-	-	-	-	-		-	-	-	-	-
Single[a]	-2.53	1.71	-5.92	0.86	.14	-	-	-	-	-		-	-	-	-	-
Divorced or separated[a]	-2.71	2.21	-7.08	1.66	.22	-	-	-	-	-		-	-	-	-	-
Student[a]	-4.25	2.60	-9.40	0.89	.10	-	-	-	-	-		-	-	-	-	-
Retired[a]	2.16	2.04	-1.88	6.20	.29	-	-	-	-	-		-	-	-	-	-
Not employed[a]	2.59	1.93	-1.24	6.41	.18	-	-	-	-	-		-	-	-	-	-
Depression[a]	-0.38	0.08	-0.53	-0.22	<.001*	-0.38	0.08	-0.53	-0.23	<.001*		-0.38	0.08	-0.53	-0.23	<.001*
Post*Gamified ABMT	-	-	-	-	-	-	-	-	-	-		-0.26	1.51	-3.25	2.73	.86
Post*Standard ABMT	-	-	-	-	-	-	-	-	-	-		-0.45	1.55	-3.52	2.63	.78
AIC	1583.79					1581.87						1585.79				
BIC	1631.86					1605.91						1616.69				

Note. ABMT=Attentional Bias Modification Training; AIC=Akaike Information Criterion; BIC=Bayesian Information Criterion; for time, baseline was the reference category; for training condition, control was the reference category; for education level, tertiary level education was the reference category; for marital status, married or in a relationship was the reference category; for work status, employed was the reference category.

[a] Covariates.

*Statistical significance *P*<.05, two-tailed.

Table S1 displays the results of the linear mixed models for attentional control. There was no significant main effect of time on attentional control scores from baseline to posttraining assessment (*P*=.81) nor a significant main effect of training condition (*P* values>.05; AIC=1581.87 and BIC=1605.91). The model that included the interaction effects did not fit the data better than the previous model and all interactions were nonsignificant for Time*Condition (*P* values>.05; AIC=1585.79 and BIC=1616.69).

Table S2. Linear Mixed Model Investigating the Role of Attentional Control on Pain Intensity

Model	B	SE	95% CI		*P*
Intercept	39.90	6.06	27.90	51.90	<.001*
Follow-up	-1.85	0.72	-3.26	-0.45	.010*
Posttraining	-0.35	0.69	-1.70	1.01	.61
Gamified ABMT	1.87	6.64	-11.28	15.01	.78
Standard ABMT	-3.84	6.64	-16.99	9.31	.56
Depression[a]	0.31	0.06	0.19	0.42	<.001*
Attentional control	0.14	0.09	-0.03	0.32	.10
Gamified ABMT*Attentional control	-0.04	0.13	-0.30	0.22	.75
Standard ABMT*Attentional control	0.10	0.13	-0.16	0.36	.45
AIC	2099.67				
BIC	2141.09				

Note. ABMT=Attentional Bias Modification Training; AIC=Akaike Information Criterion; BIC=Bayesian Information Criterion; for time, baseline was the reference category; for group, control was the reference category.

[a] Covariate.

*Statistical significance *P*<.05, two-tailed.

Table S2 displays the results of the linear mixed model exploring the role of attentional control on pain intensity. The inclusion of attentional control and its interaction with training condition did improve the model fit compared with the original model. Results showed a significant main effect of time from baseline to follow-up assessment (*P*=.010). However, there were no significant main effects of attentional control or training condition nor interaction effects of condition with attentional control.

Table S3. Linear Mixed Model Investigating the Role of Attentional Control -Total on Pain Interference

Model	B	SE	95% CI		*P*
Intercept	30.39	4.84	20.82	39.97	<.001*
Follow-up	-2.03	0.60	-3.21	-0.84	<.001*
Posttraining	-0.78	0.58	-1.93	0.36	.18
Gamified ABMT	8.71	5.41	-2.00	19.41	.11
Standard ABMT	-2.93	5.39	-13.59	7.73	.59
Single[a]	-2.90	0.98	-4.85	-0.96	.004*
Divorced or separated[a]	-0.66	1.28	-3.19	1.87	.61
Student[a]	1.14	1.50	-1.83	4.11	.45
Retired[a]	4.09	1.17	1.78	6.40	<.001*
Not employed[a]	3.47	1.11	1.27	5.66	.002*
Depression[a]	0.40	0.05	0.31	0.50	<.001*
Attentional control	0.19	0.07	0.05	0.32	.009*
Gamified ABMT*Attentional control	-0.14	0.10	-0.35	0.06	.17
Standard ABMT*Attentional control	0.07	0.11	-0.14	0.27	.54
AIC	1985.39				
BIC	2045.64				

Note. ABMT=Attentional Bias Modification Training; AIC=Akaike Information Criterion; BIC=Bayesian Information Criterion; for time, baseline was the reference category; for training condition, control was the reference category; for marital status, married or in a relationship was the reference category; for work status, employed was the reference category.

[a] Covariates.

*Statistical significance *P*<.05, two-tailed.

Table S3 displays the results of the linear mixed model exploring the role of attentional control on pain interference. The inclusion of attentional control and its interaction with training condition did improve the model fit compared with the original model. Results showed a significant main effect of attentional control (*P*=.009), and of time from baseline to follow-up assessment (*P*<.001). However, there was no significant main effect of training condition nor interaction effects of condition with attentional control.

4. EXPLORATORY ANALYSES FOR ENGAGEMENT METRICS

Table S4. Linear Mixed Model Investigating the Role of Engagement Metrics on Pain Intensity

Model	B	SE	95% CI		*P*
Intercept	51.37	4.49	42.50	60.24	<.001*
Follow-up	-1.80	0.73	-3.23	-0.37	.014*
Posttraining	-0.28	0.69	-1.64	1.09	.69
Gamified ABMT	0.77	4.31	-7.73	9.27	.86
Standard ABMT	2.30	3.67	-4.94	9.54	.53
Depression[a]	0.24	0.06	0.13	0.35	<.001*
Number of sessions completed	-0.06	0.54	-1.13	1.01	.91
Gamified ABMT*Number of sessions	-0.25	0.83	-1.88	1.39	.77
Standard ABMT*Number of sessions	-0.30	0.73	-1.73	1.14	.69
AIC	2119.00				
BIC	2160.49				

Note. ABMT=Attentional Bias Modification Training; AIC=Akaike Information Criterion; BIC=Bayesian Information Criterion; for time, baseline was the reference category; for training condition, control was the reference category.
[a] Covariate.
*Statistical significance *P*<.05, two-tailed.

Table S4 displays the results of the linear mixed model exploring the role of engagement metrics (number of training sessions completed) on pain intensity. The inclusion of engagement metrics and its interaction with training condition did not improve the model fit compared with the original model and all interactions were nonsignificant for Condition*Number of sessions.

Table S5. Linear Mixed Model Investigating the Role of Engagement Metrics on Pain Interference

Model	B	SE	95% CI		*P*
Intercept	42.41	3.63	35.25	49.58	<.001*
Follow-up	-2.00	0.62	-3.21	-0.78	.001*
Posttraining	-0.76	0.59	-1.92	0.39	.19
Gamified ABMT	2.00	3.56	-5.03	9.03	.58
Standard ABMT	4.13	3.04	-1.87	10.13	.18
Single[a]	-3.45	1.02	-5.46	-1.45	<.001*
Divorced or separated[a]	-0.95	1.31	-3.55	1.64	.47
Student[a]	0.88	1.55	-2.18	3.95	.57
Retired[a]	4.63	1.21	2.24	7.03	<.001*
Not employed[a]	4.32	1.17	2.01	6.64	<.001*
Depression[a]	0.35	0.05	0.26	0.44	<.001*
Number of sessions completed	0.05	0.45	-0.83	0.93	.91
Gamified ABMT*Number of sessions	-0.04	0.68	-1.39	1.31	.95
Standard ABMT*Number of sessions	-0.85	0.60	-2.04	0.34	.16
AIC	2008.40				
BIC	2068.74				

Note. ABMT=Attentional Bias Modification Training; AIC=Akaike Information Criterion; BIC=Bayesian Information Criterion; for time, baseline was the reference category; for training condition, control was the reference category; for marital status, married or in a relationship was the reference category; for work status, employed was the reference category.

[a] Covariates.

*Statistical significance *P*<.05, two-tailed.

Table S5 displays the results of the linear mixed model exploring the role of engagement metrics (number of training sessions completed) on pain interference. The inclusion of engagement metrics and its interaction with training condition did not improve the model fit compared with the original model and all interactions were nonsignificant for Condition*Number of training sessions.

5. EXPLORATORY ANALYSES FOR PERSONALITY CHARACTERISTICS

Table S6. Linear Mixed Model Investigating the Role of Personality – BIS Total on Pain Intensity

Model	B	SE	95% CI		*P*
Intercept	52.62	6.45	39.89	65.36	<.001*
Follow-up	-1.90	0.71	-3.30	-0.49	.008*
Posttraining	-0.35	0.68	-1.70	0.99	.61
Gamified ABMT	2.46	7.52	-12.41	17.32	.74
Standard ABMT	2.29	7.31	-12.15	16.73	.76
Depression[a]	0.29	0.06	0.17	0.41	<.001*
BIS total	-0.23	0.29	-0.79	0.34	.43
Gamified ABMT*BIS total	-0.15	0.37	-0.88	0.58	.68
Standard ABMT*BIS total	-0.08	0.36	-0.78	0.63	.83
AIC	2115.61				
BIC	2157.09				

Note. ABMT=Attentional Bias Modification Training; AIC=Akaike Information Criterion; BIC=Bayesian Information Criterion; BIS scale=Behavioral Inhibition System scale; for time, baseline was the reference category; for training condition, control was the reference category.

[a] Covariate.

*Statistical significance *P*<.05, two-tailed.

Table S6 displays the results of the linear mixed model exploring the role of personality characteristics (BIS) on pain intensity. The inclusion of BIS and its interaction with training condition did not improve the model fit compared with the original model and all interactions were nonsignificant for Condition*BIS total.

Table S7. Linear Mixed Model Investigating the Role of Personality – BIS Total on Pain Interference

Model	B	SE	95% CI		*P*
Intercept	47.64	5.36	37.06	58.23	<.001*
Follow-up	-2.10	0.61	-3.30	-0.91	<.001*
Posttraining	-0.87	0.58	-2.01	0.27	.13
Gamified ABMT	-2.78	6.18	-15.00	9.44	.65
Standard ABMT	-0.97	6.00	-12.82	10.88	.87
Single[a]	-3.51	1.02	-5.53	-1.49	<.001*
Divorced or separated[a]	-1.33	1.32	-3.95	1.28	.31
Student[a]	0.92	1.56	-2.17	4.01	.56
Retired[a]	3.81	1.24	1.37	6.25	.002*
Not employed[a]	3.62	1.16	1.32	5.91	.002*
Depression[a]	0.38	0.05	0.28	0.49	<.001*
BIS total	-0.31	0.24	-0.78	0.16	.20
Gamified ABMT*BIS total	0.21	0.30	-0.38	0.81	.48
Standard ABMT*BIS total	0.04	0.29	-0.54	0.62	.88
AIC	2009.01				
BIC	2069.36				

Note. ABMT=Attentional Bias Modification Training; AIC=Akaike Information Criterion; BIC=Bayesian Information Criterion; BIS scale=Behavioral Inhibition System scale; for time, baseline was the reference category; for training condition, control was the reference category; for marital status, married or in a relationship was the reference category; for work status, employed was the reference category.

[a] Covariates.

*Statistical significance *P*<.05, two-tailed.

Table S7 displays the results of the linear mixed model exploring the role of personality characteristics (BIS) on pain interference. The inclusion of BIS and its interaction with training condition did not improve the model fit compared with the original model and all interactions were nonsignificant for Condition*BIS total.

Table S8. Linear Mixed Model Investigating the Role of Personality– BAS Total on Pain Intensity

Model	B	SE	95% CI		*P*
Intercept	59.19	7.27	44.81	73.56	<.001*
Follow-up	-1.90	0.71	-3.31	-0.50	.008*
Posttraining	-0.37	0.68	-1.71	0.98	.59
Gamified ABMT	-8.38	8.36	-24.93	8.16	.32
Standard ABMT	-11.74	7.68	-26.92	3.45	.13
Depression[a]	0.24	0.05	0.13	0.35	<.001*
BAS total	-0.23	0.18	-0.58	0.13	.21
Gamified ABMT*BAS total	0.23	0.24	-0.24	0.69	.34
Standard ABMT*BAS total	0.37	0.22	-0.06	0.80	.095
AIC	2116.94				
BIC	2158.42				

Note. ABMT=Attentional Bias Modification Training; AIC=Akaike Information Criterion; BIC=Bayesian Information Criterion; BAS scale=Behavioral Activation System scale; for time, baseline was the reference category; for training condition, control was the reference category.

[a] Covariate.

*Statistical significance *P*<.05, two-tailed.

Table S8 displays the results of the linear mixed model exploring the role of personality characteristics (BAS) on pain intensity. The inclusion of BAS and its interaction with training condition did not improve the model fit compared with the original model and the interactions were nonsignificant for Condition*BAS total.

Table S9. Linear Mixed Model Investigating the Role of Personality Characteristics– BAS Scale Total on Pain Interference

Model	B	SE	95% CI		*P*
Intercept	55.16	5.93	43.44	66.88	<.001*
Follow-up	-2.13	0.60	-3.32	-0.94	<.001*
Posttraining	-0.89	0.58	-2.03	0.25	.12
Gamified ABMT	-7.92	6.72	-21.20	5.36	.24
Standard ABMT	-17.55	6.14	-29.68	-5.41	.005*
Single[a]	-3.17	1.03	-5.20	-1.13	.003*
Divorced or separated[a]	-1.27	1.29	-3.82	1.27	.32
Student[a]	1.37	1.53	-1.66	4.41	.37
Retired[a]	4.62	1.19	2.26	6.98	<.001*
Not employed[a]	3.94	1.14	1.68	6.19	<.001*
Depression[a]	0.33	0.05	0.24	0.42	<.001*
BAS total	-0.33	0.14	-0.61	-0.04	.025*
Gamified ABMT*BAS total	0.28	0.19	-0.10	0.65	.15
Standard ABMT*BAS total	0.52	0.18	0.17	0.86	.004*
AIC	2003.73				
BIC	2064.08				

Note. ABMT=Attentional Bias Modification Training; AIC=Akaike Information Criterion; BIC=Bayesian Information Criterion; BAS scale=Behavioral Activation System scale; for time, baseline was the reference category; for training condition, control was the reference category; for marital status, married or in a relationship was the reference category; for work status, employed was the reference category.

[a] Covariates.

*Statistical significance *P*<.05, two-tailed.

Table S9 displays the results of the linear mixed model exploring the role of personality characteristics (BAS) on pain interference. The inclusion of BAS and its interaction with training condition did improve the model fit compared with the original model. Results showed significant main effects of BAS (*P*=.025), time from baseline to follow-up assessment (*P*<.001) and training condition between the control and standard ABMT conditions (*P*=.005). The interaction between BAS and training condition was also significant for the standard ABMT*BAS compared to the control*BAS (*P*=.004), suggesting that BAS is moderating the effects of ABMT on pain interference. The results suggest that for the control and gamified ABMT conditions, BAS was negatively associated with pain interference (ie, lower BAS was associated with higher pain interference), whereas the standard ABMT condition displayed no relationship between these variables (see Figure S1). Given the novelty of this finding and the large number of exploratory analyses performed, more research is needed to clarify this relationship.

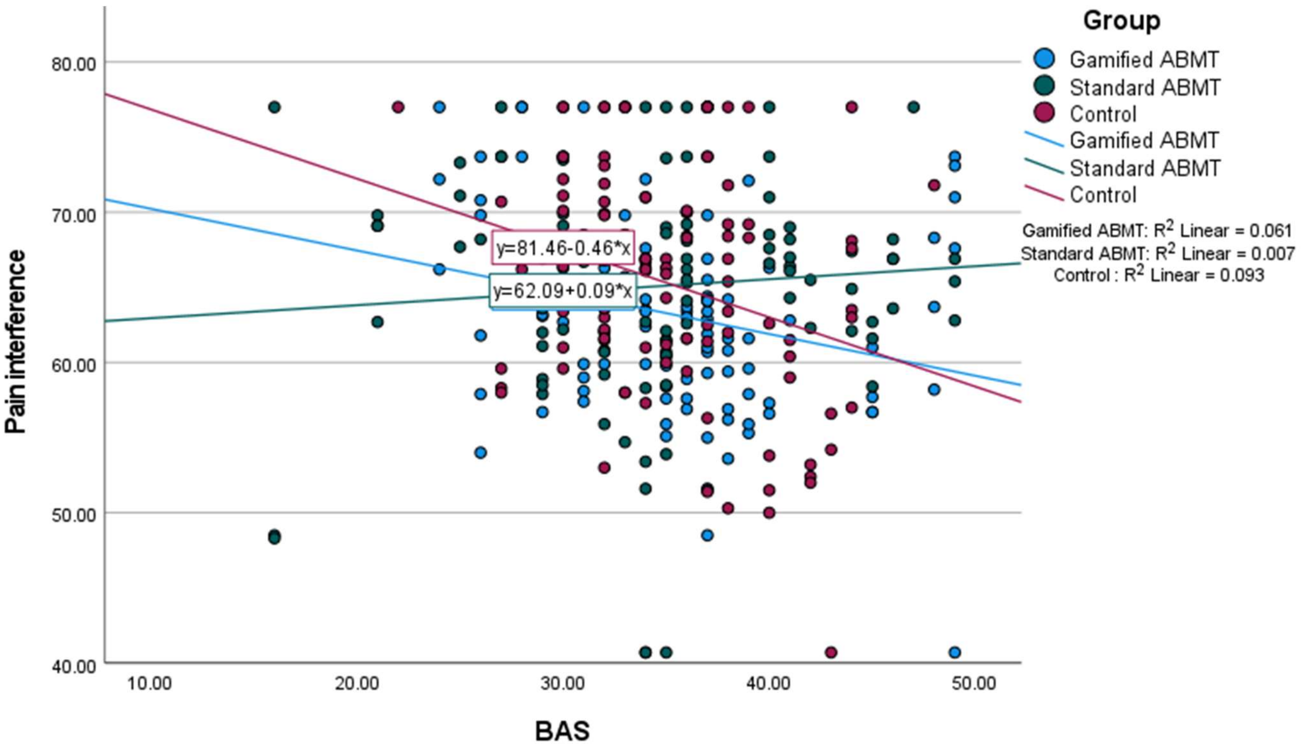

Figure S1. Scatter plot showing the interaction effects for Condition*BAS total. ABMT=attentional bias modification training.

6. EXPLORATORY ANALYSES FOR PAIN-RELATED WORRYING

Table S10. Linear Mixed Model Investigating the Role of Pain-Related Worrying on Pain Intensity

Model	B	SE	95% CI		*P*
Intercept	55.12	4.28	46.66	63.57	<.001*
Follow-up	-1.93	0.71	-3.33	-0.53	.007*
Posttraining	-0.39	0.68	-1.73	0.96	.57
Gamified ABMT	3.77	2.63	-1.44	8.98	.15
Standard ABMT	2.04	2.71	-3.33	7.41	.45
Depression[a]	0.07	0.07	-0.08	0.21	.37
Pain-related worrying	0.26	0.08	0.11	0.41	<.001*
Gamified ABMT*Pain-related worrying	-0.18	0.09	-0.37	0.01	.06
Standard ABMT*Pain-related worrying	-0.03	0.10	-0.21	0.16	.79
AIC	2103.68				
BIC	2145.17				

Note. ABMT=Attentional Bias Modification Training; AIC=Akaike Information Criterion; BIC=Bayesian Information Criterion; for time, baseline was the reference category; for training condition, control was the reference category.
[a] Covariate.
*Statistical significance *P*<.05, two-tailed.

Table S10 displays the results of the linear mixed model exploring the role of pain-related worrying on pain intensity. The inclusion of pain-related worrying and its interaction with training condition did improve the model fit compared with the original model. Results showed significant main effects of pain-related worrying (*P*<.001) and time from baseline to follow-up assessment (*P*=.007). However, there was no significant main effect of training condition nor interaction effects of condition with pain-related worrying.

Table S11. Linear Mixed Model Investigating the Role of Pain-Related Worrying on Pain Interference

Model	B	SE	95% CI		*P*
Intercept	48.22	3.62	41.07	55.36	<.001*
Follow-up	-2.12	0.60	-3.31	-0.93	<.001*
Posttraining	-0.89	0.58	-2.03	0.25	.12
Gamified ABMT	1.81	2.27	-2.68	6.30	.43
Standard ABMT	0.55	2.33	-4.06	5.16	.81
Single[a]	-3.16	1.00	-5.14	-1.18	.002*
Divorced or separated[a]	-0.09	1.31	-2.68	2.51	.95
Student[a]	1.14	1.53	-1.88	4.16	.46
Retired[a]	3.90	1.20	1.53	6.26	.001*
Not employed[a]	3.92	1.13	1.68	6.16	<.001*
Depression[a]	0.20	0.06	0.07	0.32	.003*
Pain-related worrying	0.16	0.07	0.03	0.29	.017*
Gamified ABMT*Pain-related worrying	-0.02	0.08	-0.19	0.14	.77
Standard ABMT*Pain-related worrying	-0.01	0.08	-0.17	0.16	.92
AIC	2002.03				
BIC	2062.37				

Note. ABMT=Attentional Bias Modification Training; AIC=Akaike Information Criterion; BIC=Bayesian Information Criterion; for time, baseline was the reference category; for training condition, control was the reference category; for marital status, married or in a relationship was the reference category; for work status, employed was the reference category.

[a] Covariates.

*Statistical significance *P*<.05, two-tailed.

Table S11 displays the results of the linear mixed model exploring the role of pain-related worrying on pain interference. The inclusion of pain-related worrying and its interaction with training condition did improve the model fit compared with the original model. Results showed significant main effects of pain-related worrying (*P*=.017) and time from baseline to follow-up assessment (*P*<.001). However, there was no significant main effect of training condition nor interaction effects of condition with pain-related worrying.

7. EXPLORATORY ANALYSES FOR RECRUITMENT SETTING

Table S12. Linear Mixed Model Investigating the Role of Recruitment Setting on Pain Intensity

Model	B	SE	95% CI		*P*
Intercept	52.81	3.30	46.29	59.33	<.001*
Follow-up	-2.04	0.71	-3.45	-0.63	.005*
Posttraining	-0.49	0.68	-1.83	0.86	.48
Gamified ABMT	0.24	1.41	-2.54	3.03	.86
Standard ABMT	0.63	1.46	-2.27	3.53	.67
Depression[a]	0.19	0.05	0.08	0.29	<.001*
Clinical setting	4.84	1.70	1.47	8.20	.005*
Gamified ABMT*Clinical setting	-0.95	2.70	-6.29	4.40	.73
Standard ABMT*Clinical setting	0.92	2.42	-3.87	5.72	.70
AIC	2100.35				
BIC	2141.84				

Note. ABMT=Attentional Bias Modification Training; AIC=Akaike Information Criterion; BIC=Bayesian Information Criterion; for time, baseline was the reference category; for training condition, control was the reference category; for recruitment setting, nonclinical setting was the reference group.

[a] Covariate.

*Statistical significance *P*<.05, two-tailed.

Table S12 displays the results of the linear mixed model exploring the role of recruitment setting on pain intensity. The inclusion of recruitment setting and its interaction with training condition did improve the model fit compared with the original model. Results showed a significant main effect of time from baseline to follow-up assessment (*P*=.005). Moreover, participants' location of recruitment had a significant impact on their pain intensity ratings so that pain intensity was 4.84 unit higher for participants recruited from the hospital outpatient waiting list compared to those recruited from the general population (*P*=.005). However, there was no significant main effect of training condition nor interaction effects of condition with clinical setting.

Table S13. Linear Mixed Models Investigating the Role of Recruitment Setting on Pain Interference

Model	B	SE	95% CI		*P*
Intercept	45.10	2.88	39.40	50.80	<.001*
Follow-up	-2.17	0.61	-3.37	-0.98	<.001*
Posttraining	-0.94	0.58	-2.08	0.21	.11
Gamified ABMT	1.20	1.28	-1.33	3.73	.35
Standard ABMT	-0.89	1.29	-3.44	1.66	.49
Single[a]	-3.45	1.00	-5.42	-1.48	<.001*
Divorced or separated[a]	-1.56	1.32	-4.17	1.06	.24
Student[a]	0.86	1.53	-2.16	3.88	.58
Retired[a]	4.01	1.20	1.64	6.39	.001*
Not employed[a]	4.06	1.14	1.81	6.32	<.001*
Depression[a]	0.32	0.05	0.22	0.41	<.001*
Clinical setting	0.51	1.48	-2.43	3.44	.73
Gamified ABMT*Clinical setting	2.18	2.39	-2.55	6.90	.36
Standard ABMT*Clinical setting	2.97	2.09	-1.16	7.10	.16
AIC	2005.40				
BIC	2065.74				

Note. ABMT=Attentional Bias Modification Training; AIC=Akaike Information Criterion; BIC=Bayesian Information Criterion; for time, baseline was the reference category; for training condition, control was the reference category; for marital status, married or in a relationship was the reference category; for work status, employed was the reference category; for recruitment setting, nonclinical setting was the reference group.

[a] Covariates.

*Statistical significance *P*<.05, two-tailed.

Table S13 displays the results of the linear mixed model exploring the role of recruitment setting on pain interference. The inclusion of recruitment setting and its interaction with training condition did improve the model fit compared with the original model. Results showed a significant main effect of time from baseline to follow-up assessment (*P*<.001). However, participants' location of recruitment had no significant impact on their pain interference ratings. There was also no significant main effect of training condition nor interaction effects of condition with clinical setting.

8. SUMMARY STATISTICS ON ADDITIONAL AND EXPLORATORY ANALYSES

Table S14. Summary Statistics on Outcome Measures by Training Condition and Assessment Time Points

Variable	Training condition					
	Standard ABMT		Gamified ABMT		Control	
	n	M (SD) or %	n	M (SD) or %	n	M (SD) or %
**ACS**						
Baseline	42	49.95 (9.54)	41	50.76 (9.36)	45	50.13 (9.76)
Posttraining	31	48.58 (9.24)	35	50.40 (11.50)	35	50.11 (8.90)
**BIS scale total**	43	19.44 (4.22)	41	19.51 (3.80)	45	20.56 (3.33)
**BAS scale total**	43	34.47 (7.16)	41	35.73 (5.95)	45	34.67 (5.10)
**PCS total**	43	24.28 (13.41)	41	22.05 (13.61)	45	26.69 (12.13)
**Medical visit in the past 4 weeks**						
Baseline, yes	33	76.7	28	68.3	34	75.6
Posttraining, yes	18	56.3	24	68.6	24	68.6
Follow-up, yes	15	51.7	15	53.6	21	63.6
**Use of medication**						
Baseline, yes	41	95.3	41	100	44	97.8
Posttraining, yes	28	87.5	33	94.3	34	97.1
Follow-up, yes	24	82.8	28	100	29	87.9
**Pain treatment**						
Baseline, yes	38	88.4	38	92.7	38	84.4
Posttraining, yes	23	71.9	31	88.6	25	71.4
Follow-up, yes	23	79.3	23	82.1	22	66.7
**Treatment received**						
Placebo	25	78.1	28	80	28	80
Intervention	7	21.9	7	20	7	20

Note. SD=standard deviation; ABMT=Attentional Bias Modification Training; ACS=Attentional Control Scale; BIS and BAS scales=Behavioral Inhibition System and Behavioral Activation System scales; PCS=Pain Catastrophizing Scale.
*Statistical significance *P*< .05, two-tailed.

9. SUMMARY STATISTICS FOR LINEAR MIXED MODEL EXPLORATORY ANALYSES

Table S15. Summary Statistics on Attentional Control Measure by Training Condition Across Time

Variable	Standard ABMT			Gamified ABMT			Control		
	Obs	M	SD	Obs	M	SD	Obs	M	SD
Attentional control	73	49.37	9.38	76	50.59	10.33	80	50.13	9.33

Note. Obs=observations.

Table S16. Summary Statistics on Attentional Control Measure by Assessment Time Points

Variable	Baseline			Posttraining			Follow-up		
	n	M	SD	n	M	SD	n	M	SD
Attentional control	128	50.27	9.49	101	49.74	9.91	-	-	-

Reference

1. Derryberry, D. and M.A. Reed, *Anxiety-related attentional biases and their regulation by attentional control.* Journal of abnormal psychology, 2002. **111**(2): p. 225-236.
2. Heathcote, L.C., et al., *Attention bias modification training for adolescents with chronic pain: a randomized placebo-controlled trial.* Pain, 2018.
3. Carver, C.S. and T.L. White, *Behavioral inhibition, behavioral activation, and affective responses to impending reward and punishment: the BIS/BAS scales.* Journal of Personality and Social Psychology, 1994. **67**(2): p. 319-333.
4. Meyer, B., S.L. Johnson, and C.S. Carver, *Exploring Behavioral Activation and Inhibition Sensitivities Among College Students at Risk for Bipolar Spectrum Symptomatology.* J Psychopathol Behav Assess, 1999. **21**(4): p. 275-292.
5. Sullivan, M.J.L., S.R. Bishop, and J. Pivik, *The pain catastrophizing scale: development and validation.* Psychological assessment, 1995. **7**(4): p. 524-532.
6. Crombez, G., et al., *Let's talk about pain catastrophizing measures: an item content analysis.* PeerJ, 2020. **8**: p. e8643.
7. Van Damme, S., et al., *A confirmatory factor analysis of the Pain Catastrophizing Scale: invariant factor structure across clinical and non-clinical populations.* Pain, 2002. **96**(3): p. 319-324.
8. Vermeir, J.F., et al., *Gamified web-delivered attentional bias modification training for adults with chronic pain: Protocol for a randomized, double-blind, placebo-controlled trial.* JMIR Res Protoc, 2022. **11**(1): p. e32359.
