# Supplementary material for: Gamified Web-Delivered Attentional Bias Modification Training for Adults With Chronic Pain: Randomized, Double-Blind, Placebo-Controlled Trial
Source: JMIR Serious Games. 2025 Jan 16;13:e50635. doi: 10.2196/50635 (PMC11783034; doi:10.2196/50635)
Supplement: Multimedia Appendix 3 [file games_v13i1e50635_app3.pdf]

Multimedia Appendix 3: Primary and Secondary Outcomes

Gamified Web-Delivered Attentional Bias Modification Training for Adults with Chronic Pain: Randomized, Double-Blind, Placebo-Controlled Trial

Julie F Vermeir[1], PhD; Melanie J White[1], PhD; Daniel Johnson[2], PhD; Geert Crombez[3], PhD; Dimitri M L Van Ryckeghem[3,4,5], PhD

[1]School of Psychology and Counselling, Faculty of Health, Queensland University of Technology (QUT), Brisbane, Australia

[2]School of Computer Science, Faculty of Science, Queensland University of Technology (QUT), Brisbane, Australia

[3]Department of Experimental Clinical and Health Psychology, Ghent University, Ghent, Belgium

[4]Department of Clinical Psychological Science, Maastricht University, Maastricht, Netherlands

[5]Department of Behavioural and Cognitive Sciences, University of Luxembourg, Esch-sur-Alzette, Luxembourg

***Corresponding author:** Julie F Vermeir. *Address*: Faculty of Health, School of Psychology and Counselling, Queensland University of Technology (QUT), 170 Victoria Park Road, Brisbane, QLD, 4059, Australia. *Phone*: +61 731384714. *Email address*: julie.vermeir@hdr.qut.edu.au

Trial Registration: Australian New Zealand Clinical Trials Registry ACTRN12620000803998; https://anzctr.org.au/ACTRN12620000803998.aspx

International Registered Report Identifier (IRRID): PRR1-10.2196/32359

1. ANALYSES FOR PRIMARY OUTCOMES

Table S1. Linear Mixed Models for Task-Related Engagement

	Model 1: Main effects with covariates					Model 2: Removing nonsignificant covariate(s)					Model 3: Adding interaction terms to the best fit model				
	B	SE	95% CI		*P*	B	SE	95% CI		*P*	B	SE	95% CI		*P*
Intercept	6.77	1.62	3.58	9.97	<.001*	5.72	0.59	4.55	6.90	<.001*	5.60	0.61	4.40	6.79	<.001*
Session 6	-1.63	0.19	-2.00	-1.25	<.001*	-1.63	0.19	-2.00	-1.25	<.001*	-1.39	0.31	-2.01	-0.78	<.001*
Session 5	-1.33	0.19	-1.71	-0.96	<.001*	-1.33	0.19	-1.71	-0.96	<.001*	-1.03	0.32	-1.66	-0.41	.001*
Session 4	-1.28	0.19	-1.65	-0.91	<.001*	-1.28	0.19	-1.65	-0.91	<.001*	-1.11	0.30	-1.71	-0.52	<.001*
Session 3	-0.99	0.18	-1.35	-0.63	<.001*	-0.99	0.18	-1.35	-0.63	<.001*	-1.00	0.30	-1.59	-0.41	<.001*
Session 2	-0.69	0.18	-1.04	-0.34	<.001*	-0.69	0.18	-1.03	-0.34	<.001*	-0.46	0.29	-1.04	0.11	.12
Gamified ABMT	0.57	0.68	-0.78	1.91	.41	0.55	0.67	-0.77	1.87	.41	0.66	0.71	-0.74	2.06	.35
Standard ABMT	-0.09	0.61	-1.30	1.12	.88	-0.20	0.60	-1.38	0.98	.74	0.09	0.64	-1.18	1.36	.89
Primary[a]	3.47	2.73	-1.93	8.86	.21	-	-	-	-	-	-	-	-	-	-
Secondary[a]	0.22	0.63	-1.02	1.46	.73	-	-	-	-	-	-	-	-	-	-
Single[a]	-1.38	0.57	-2.51	-0.25	.017*	-1.54	0.56	-2.64	-0.43	.007*	-1.53	0.56	-2.64	-0.43	.007*
Divorced or separated[a]	0.28	0.74	-1.19	1.75	.71	0.22	0.75	-1.26	1.69	.77	0.24	0.74	-1.23	1.71	.75
Student[a]	1.20	0.88	-0.53	2.94	.17	1.09	0.87	-0.64	2.81	.22	1.08	0.87	-0.64	2.80	.22
Retired[a]	1.63	0.68	0.29	2.98	.018*	1.71	0.67	0.37	3.04	.012*	1.69	0.67	0.37	3.02	.013*
Not employed[a]	0.79	0.65	-0.50	2.08	.23	0.67	0.63	-0.58	1.92	.29	0.66	0.63	-0.58	1.91	.29
Depression[a]	-0.02	0.03	-0.07	0.03	.44	-	-	-	-	-	-	-	-	-	-
Session 6*Gamified ABMT	-	-	-	-	-	-	-	-	-	-	-0.41	0.45	-1.29	0.47	.36
Session 6*Standard ABMT	-	-	-	-	-	-	-	-	-	-	-0.31	0.47	-1.23	0.62	.51
Session 5*Gamified ABMT	-	-	-	-	-	-	-	-	-	-	-0.61	0.46	-1.51	0.29	.18
Session 5*Standard ABMT	-	-	-	-	-	-	-	-	-	-	-0.34	0.47	-1.26	0.59	.48
Session 4*Gamified ABMT	-	-	-	-	-	-	-	-	-	-	-0.13	0.45	-1.01	0.74	.77
Session 4*Standard ABMT	-	-	-	-	-	-	-	-	-	-	-0.41	0.46	-1.31	0.49	.37
Session 3*Gamified ABMT	-	-	-	-	-	-	-	-	-	-	0.27	0.43	-0.58	1.11	.54
Session 3*Standard ABMT	-	-	-	-	-	-	-	-	-	-	-0.31	0.45	-1.20	0.58	.50
Session 2*Gamified ABMT	-	-	-	-	-	-	-	-	-	-	-0.14	0.42	-0.96	0.69	.75
Session 2*Standard ABMT	-	-	-	-	-	-	-	-	-	-	-0.60	0.44	-1.46	0.26	.17
AIC	2387.82					2384.19					2396.60				
BIC	2466.75					2449.96					2506.23				

Note. ABMT=Attentional Bias Modification Training; AIC=Akaike Information Criterion; BIC=Bayesian Information Criterion; for session, session 1 was the reference category; for training condition, control was the reference category; for education level, tertiary level education was the reference category; for marital status, married or in a relationship was the reference category; for work status, employed was the reference category.

[a] Covariates.

*Statistical significance *P*<.05, two-tailed

Table S2. Linear Mixed Models for Pain Intensity

	**Model 1: Main effects with covariates**					**Model 2: Removing nonsignificant covariate(s)**					**Model 3: Adding interaction terms to the best fit model**				
	B	SE	95% CI		*P*	B	SE	95% CI		*P*	B	SE	95% CI		*P*
Intercept	50.40	3.48	43.52	57.28	<.001*	51.17	3.50	44.24	58.10	<.001*	51.18	44.15	58.20	14.41	<.001*
Follow-up	-1.91	0.71	-3.31	-0.51	.008*	-1.90	0.71	-3.31	-0.50	.008*	-1.35	-3.65	0.95	-1.16	.25
Posttraining	-0.38	0.68	-1.72	0.97	.58	-0.36	0.68	-1.70	0.99	.60	-0.14	-2.39	2.11	-0.12	.90
Gamified ABMT	1.28	1.45	-1.60	4.15	.38	-0.48	1.31	-3.06	2.11	.72	0.83	-2.19	3.85	0.54	.59
Standard ABMT	1.87	1.31	-0.73	4.46	.16	1.01	1.27	-1.50	3.52	.43	0.35	-2.58	3.29	0.24	.81
Primary[a]	-1.23	5.68	-12.49	10.02	.83	-	-	-	-	-	-	-	-	-	-
Secondary[a]	1.81	1.34	-0.83	4.46	.18	-	-	-	-	-	-	-	-	-	-
Single[a]	-1.65	1.23	-4.08	0.79	.18	-	-	-	-	-	-	-	-	-	-
Divorced or separated[a]	-0.28	1.59	-3.43	2.86	.86	-	-	-	-	-	-	-	-	-	-
Student[a]	0.25	1.87	-3.46	3.96	.89	-	-	-	-	-	-	-	-	-	-
Retired[a]	2.87	1.47	-0.03	5.77	.053	-	-	-	-	-	-	-	-	-	-
Not employed[a]	2.10	1.39	-0.65	4.85	.13	-	-	-	-	-	-	-	-	-	-
Depression[a]	0.22	0.06	0.11	0.34	<.001*	0.24	0.06	0.13	0.35	<.001*	0.24	0.13	0.35	4.32	<.001*
Follow*Gamified ABMT	-	-	-	-	-	-	-	-	-	-	-2.61	-5.98	0.76	-1.53	.13
Follow*Standard ABMT	-	-	-	-	-	-	-	-	-	-	0.90	-2.44	4.24	0.53	.60
Post*Gamified ABMT	-	-	-	-	-	-	-	-	-	-	-2.11	-5.31	1.08	-1.30	.19
Post*Standard ABMT	-	-	-	-	-	-	-	-	-	-	1.57	-1.68	4.82	0.95	.34
AIC	2118.87					2113.76					2115.34				
BIC	2175.44					2143.93					2160.60				

Note. ABMT=Attentional Bias Modification Training; AIC=Akaike Information Criterion; BIC=Bayesian Information Criterion; for time, baseline was the reference category; for training condition, control was the reference category; for education level, tertiary level education was the reference category; for marital status, married or in a relationship was the reference category; for work status, employed was the reference category.

[a] Covariates.

*Statistical significance *P*<.05, two-tailed.

Table S3. Linear Mixed Models for Pain Interference

	**Model 1: Main effects with covariates**					**Model 2: Removing nonsignificant covariate(s)**					**Model 3: Adding interaction terms to the best fit model**				
	B	SE	95% CI		*P*	B	SE	95% CI		*P*	B	SE	95% CI		*P*
Intercept	43.76	2.89	38.04	49.48	<.001*	43.53	2.89	37.83	49.24	<.001*	43.64	2.91	37.90	49.38	<.001*
Follow-up	-2.12	0.61	-3.32	-0.93	<.001*	-2.12	0.61	-3.31	-0.93	<.001*	-3.57	0.99	-5.52	-1.61	<.001*
Posttraining	-0.88	0.58	-2.03	0.26	.13	-0.88	0.58	-2.02	0.26	.13	-0.47	0.97	-2.38	1.45	.63
Gamified ABMT	1.78	1.21	-0.61	4.17	.14	1.63	1.19	-0.72	3.99	.17	0.97	1.37	-1.72	3.66	.48
Standard ABMT	0.44	1.09	-1.72	2.60	.69	0.27	1.06	-1.84	2.37	.80	0.11	1.24	-2.34	2.56	.93
Primary[a]	-2.19	4.72	-11.53	7.15	.64	-	-	-	-	-	-	-	-	-	-
Secondary[a]	0.94	1.11	-1.26	3.14	.401	-	-	-	-	-	-	-	-	-	-
Single[a]	-3.27	1.02	-5.29	-1.25	.002*	-3.32	1.02	-5.34	-1.30	.001*	-3.32	1.02	-5.34	-1.31	.001*
Divorced or separated[a]	-1.05	1.32	-3.67	1.56	.43	-1.11	1.32	-3.72	1.51	.41	-1.12	1.32	-3.73	1.49	.40
Student[a]	0.87	1.56	-2.21	3.96	.58	0.77	1.56	-2.32	3.86	.62	0.74	1.56	-2.34	3.81	.64
Retired[a]	4.40	1.22	1.99	6.81	<.001*	4.40	1.21	2.01	6.78	<.001*	4.42	1.20	2.04	6.80	<.001*
Not employed[a]	3.90	1.16	1.61	6.19	<.001*	3.94	1.16	1.65	6.23	<.001*	3.90	1.16	1.61	6.18	<.001*
Depression[a]	0.33	0.05	0.24	0.43	<.001*	0.34	0.05	0.25	0.43	<.001*	0.34	0.05	0.25	0.44	<.001*
Follow*Gamified ABMT	-	-	-	-	-	-	-	-	-	-	3.07	1.46	0.20	5.94	.036*
Follow*Standard ABMT	-	-	-	-	-	-	-	-	-	-	1.53	1.45	-1.32	4.38	.29
Post*Gamified ABMT	-	-	-	-	-	-	-	-	-	-	-0.39	1.38	-3.12	2.34	.78
Post*Standard ABMT	-	-	-	-	-	-	-	-	-	-	-0.85	1.41	-3.62	1.92	.55
AIC	2009.30					2006.30					2007.61				
BIC	2065.87					2055.33					2071.72				

Note. ABMT=Attentional Bias Modification Training; AIC=Akaike Information Criterion; BIC=Bayesian Information Criterion; for time, baseline was the reference category; for training condition, control was the reference category; for education level, tertiary level education was the reference category; for marital status, married or in a relationship was the reference category; for work status, employed was the reference category.

[a] Covariates.

*Statistical significance *P*<.05, two-tailed.

2. ANALYSES FOR SECONDARY OUTCOMES

Table S4. Linear Mixed Models for Attentional Bias Index

	**Model 1: Main effects with covariates**					**Model 2: Removing nonsignificant covariate(s)**					**Model 3: Adding interaction terms to the best fit model**				
	B	SE	95% CI		*P*	B	SE	95% CI		*P*	B	SE	95% CI		*P*
Intercept	-2.67	9.35	-21.1	15.77	.78	-0.91	2.55	-5.93	4.12	.72	1.30	2.96	-4.53	7.13	.66
Posttraining	2.20	2.73	-3.17	7.578	.42	2.39	2.76	-3.05	7.82	.39	-2.95	4.60	-12.02	6.12	.52
Gamified ABMT	-1.22	3.81	-8.73	6.30	.75	-0.57	3.27	-7.01	5.88	.86	-5.02	4.26	-13.41	3.38	.24
Standard ABMT	0.29	3.53	-6.67	7.26	.93	0.45	3.33	-6.12	7.02	.89	-1.88	4.29	-10.33	6.57	.66
Primary[a]	10.47	14.35	-17.83	38.76	.47	-	-	-	-	-	-	-	-	-	-
Secondary[a]	0.63	3.59	-6.45	7.72	.86	-	-	-	-	-	-	-	-	-	-
Single[a]	-2.01	3.24	-8.40	4.39	.54	-	-	-	-	-	-	-	-	-	-
Divorced or separated[a]	-0.44	4.11	-8.54	7.65	.91	-	-	-	-	-	-	-	-	-	-
Student[a]	7.98	4.95	-1.79	17.74	.11	-	-	-	-	-	-	-	-	-	-
Retired[a]	-4.01	3.93	-11.76	3.74	.31	-	-	-	-	-	-	-	-	-	-
Not employed[a]	-0.88	3.71	-8.19	6.44	.81	-	-	-	-	-	-	-	-	-	-
Depression[a]	0.05	0.16	-0.26	0.35	.76	-	-	-	-	-	-	-	-	-	-
Post*Gamified ABMT	-	-	-	-	-	-	-	-	-	-	10.65	6.59	-2.33	23.63	.11
Post*Standard ABMT	-	-	-	-	-	-	-	-	-	-	5.66	6.75	-7.65	18.96	.40
AIC	1888.60					1878.14					1879.54				
BIC	1935.59					1898.28					1906.39				

Note. ABMT=Attentional Bias Modification Training; AIC=Akaike Information Criterion; BIC=Bayesian Information Criterion; for time, baseline was the reference category; for training condition, control was the reference category; for education level, tertiary level education was the reference category; for marital status, married or in a relationship was the reference category; for work status, employed was the reference category.

[a] Covariates.

*Statistical significance *P*<.05, two-tailed.

Table S5. Pearson's Correlations Showing the Relationships between Changes in Attentional Bias Magnitude and Changes on Pain Intensity, Pain Interference, Anxiety and Depression Measures from Pretraining to Posttraining Assessment

Variable	**Pain intensity**		**Pain interference**		**Anxiety**		**Depression**	
	r	*P*	r	*P*	r	*P*	r	*P*
**Attentional bias**								
Standard ABMT	-.05	.83	-.23	.29	-.17	.43	-.11	.63
Gamified ABMT	-.23	.22	-.29	.12	.01	.94	.06	.76
Control	.33	.076	-.01	.97	.14	.46	-.08	.68

Note. ABMT=Attentional Bias Modification Training.

Table S6. Linear Mixed Models for Anxiety

	Model 1: Main effects with covariates					Model 2: Removing nonsignificant covariate(s)					Model 3: Adding interaction terms to the best fit model				
	B	SE	95% CI		*P*	B	SE	95% CI		*P*	B	SE	95% CI		*P*
Intercept	15.89	3.42	9.12	22.66	<.001*	16.40	3.45	9.58	23.22	<.001*	17.05	3.48	10.18	23.93	<.001*
Follow-up	-1.21	0.68	-2.55	0.13	.077	-1.16	0.68	-2.51	0.18	.089	-2.03	1.12	-4.25	0.18	.071
Posttraining	-0.45	0.65	-1.74	0.83	.49	-0.43	0.65	-1.71	0.86	.51	-1.94	1.10	-4.11	0.23	.079
Gamified ABMT	1.84	1.43	-0.99	4.67	.20	1.46	1.29	-1.09	4.00	.26	0.16	1.49	-2.79	3.10	.92
Standard ABMT	0.23	1.29	-2.33	2.78	.86	-0.06	1.25	-2.54	2.42	.96	-0.81	1.45	-3.67	2.05	.58
Primary[a]	1.77	5.60	-9.34	12.87	.75	-	-	-	-	-	-	-	-	-	-
Secondary[a]	1.80	1.32	-0.81	4.41	.18	-	-	-	-	-	-	-	-	-	-
Single[a]	-1.76	1.21	-4.15	0.64	.15	-	-	-	-	-	-	-	-	-	-
Divorced or separated[a]	-2.07	1.57	-5.16	1.03	.19	-	-	-	-	-	-	-	-	-	-
Student[a]	1.97	1.85	-1.68	5.62	.29	-	-	-	-	-	-	-	-	-	-
Retired[a]	-1.68	1.44	-4.53	1.18	.25	-	-	-	-	-	-	-	-	-	-
Not employed[a]	-1.70	1.37	-4.41	1.01	.22	-	-	-	-	-	-	-	-	-	-
Depression[a]	0.74	0.06	0.62	0.85	<.001*	0.71	0.05	0.61	0.82	<.001*	0.71	0.05	0.61	0.82	<.001*
Follow*Gamified ABMT	-	-	-	-	-	-	-	-	-	-	2.10	1.65	-1.15	5.34	.20
Follow*Standard ABMT	-	-	-	-	-	-	-	-	-	-	0.60	1.63	-2.62	3.82	.71
Post*Gamified ABMT	-	-	-	-	-	-	-	-	-	-	2.54	1.56	-0.54	5.62	.11
Post*Standard ABMT	-	-	-	-	-	-	-	-	-	-	2.06	1.59	-1.07	5.19	.20
AIC	2095.90					2091.03					2095.35				
BIC	2152.47					2121.20					2140.60				

Note. ABMT=Attentional Bias Modification Training; AIC=Akaike Information Criterion; BIC=Bayesian Information Criterion; for time, baseline was the reference category; for training condition, control was the reference category; for education level, tertiary level education was the reference category; for marital status, married or in a relationship was the reference category; for work status, employed was the reference category.

[a] Covariates.

*Statistical significance *P*<.05, two-tailed.

Table S7. Linear Mixed Models for Depression

	Model 1: Main effects with covariates					Model 2: Adding interaction terms				
	B	SE	95% CI		*P*	B	SE	95% CI		*P*
Intercept	54.65	1.94	50.81	58.49	<.001*	55.15	1.98	51.244	59.061	<.001*
Follow-up	-1.17	0.70	-2.54	0.20	.094	-1.83	1.15	-4.09	0.44	.11
Posttraining	-1.82	0.66	-3.12	-0.51	.007*	-3.14	1.13	-5.36	-0.92	.006*
Gamified ABMT	-0.90	2.10	-5.06	3.26	.67	-1.63	2.23	-6.03	2.78	.47
Standard ABMT	2.07	1.90	-1.68	5.82	.28	1.19	2.02	-2.80	5.19	.56
Primary[a]	-8.46	8.39	-25.08	8.16	.32	-8.35	8.38	-24.94	8.24	.32
Secondary[a]	5.04	1.92	1.24	8.85	.010*	5.06	1.92	1.26	8.86	.009*
Single[a]	3.94	1.73	0.51	7.37	.025*	4.01	1.73	0.58	7.44	.022*
Divorced or separated[a]	-0.78	2.31	-5.36	3.79	.74	-0.83	2.31	-5.39	3.74	.72
Student[a]	5.89	2.69	0.57	11.22	.030*	5.91	2.69	0.59	11.22	.03*
Retired[a]	3.14	2.10	-1.02	7.31	.14	3.19	2.10	-0.97	7.34	.13
Not employed[a]	7.09	1.95	3.22	10.95	<.001*	7.06	1.95	3.20	10.92	<.001*
Follow*Gamified ABMT	-	-	-	-	-	1.21	1.68	-2.11	4.53	.47
Follow*Standard ABMT	-	-	-	-	-	0.82	1.68	-2.49	4.12	.63
Post*Gamified ABMT	-	-	-	-	-	1.56	1.60	-1.58	4.71	.33
Post*Standard ABMT	-	-	-	-	-	2.51	1.63	-0.70	5.72	.12
AIC	2196.33					2201.58				
BIC	2249.13					2269.47				

Note. ABMT=Attentional Bias Modification Training; AIC=Akaike Information Criterion; BIC=Bayesian Information Criterion; for time, baseline was the reference category; for training condition, control was the reference category; for education level, tertiary level education was the reference category; for marital status, married or in a relationship was the reference category; for work status, employed was the reference category.

[a] Covariates.

*Statistical significance *P*<.05, two-tailed.

Table S8. Linear Mixed Models for Perceived Improvement

	Model 1: Main effects with covariates					Model 2: Removing nonsignificant covariate(s)					Model 3: Adding interaction terms to the best fit model				
	B	SE	95% CI		*P*	B	SE	95% CI		*P*	B	SE	95% CI		*P*
Intercept	3.42	0.47	2.49	4.35	<.001*	3.76	0.18	3.40	4.12	<.001*	3.73	0.19	3.36	4.11	<.001*
Follow-up	-0.03	0.09	-0.22	0.15	.73	-0.03	0.09	-0.22	0.15	.73	0.02	0.16	-0.28	0.33	.88
Gamified ABMT	0.29	0.19	-0.09	0.66	.14	0.20	0.19	-0.17	0.57	.28	0.25	0.21	-0.17	0.67	.23
Standard ABMT	0.10	0.18	-0.25	0.46	.56	0.11	0.18	-0.24	0.46	.53	0.14	0.21	-0.27	0.55	.49
Primary[a]	-1.94	0.69	-3.30	-0.58	.006*	-1.94	0.69	-3.30	-0.58	.006*	-1.94	0.69	-3.30	-0.58	.006*
Secondary[a]	0.06	0.17	-0.29	0.40	.74	0.11	0.17	-0.24	0.45	.54	0.10	0.17	-0.24	0.45	.55
Single[a]	-0.16	0.17	-0.49	0.17	.33	-	-	-	-	-	-	-	-	-	-
Divorced or separated[a]	-0.29	0.21	-0.70	0.12	.17	-	-	-	-	-	-	-	-	-	-
Student[a]	0.00	0.25	-0.49	0.50	.99	-0.04	0.24	-0.52	0.45	.88	-0.04	0.24	-0.52	0.45	.89
Retired[a]	0.19	0.20	-0.20	0.58	.34	0.20	0.20	-0.19	0.58	.32	0.20	0.20	-0.19	0.58	.32
Not employed[a]	0.40	0.18	0.03	0.76	.032*	0.42	0.18	0.07	0.77	.020*	0.42	0.18	0.07	0.77	.020*
Depression[a]	0.01	0.01	-0.01	0.02	.38	-	-	-	-	-	-	-	-	-	-
Follow*Gamified ABMT	-	-	-	-	-	-	-	-	-	-	-0.11	0.23	-0.56	0.34	.62
Follow*Standard ABMT	-	-	-	-	-	-	-	-	-	-	-0.06	0.23	-0.51	0.38	.78
AIC	471.34					468.12					471.87				
BIC	516.94					503.95					514.22				

Note. ABMT=Attentional Bias Modification Training; AIC=Akaike Information Criterion; BIC=Bayesian Information Criterion; for time, posttraining was the reference category; for training condition, control was the reference category; for education level, tertiary level education was the reference category; for marital status, married or in a relationship was the reference category; for work status, employed was the reference category.

[a] Covariates.

*Statistical significance *P*<.05, two-tailed.

Table S9. Linear Mixed Models for Pain Interpretation Bias: Bodily Threat and Pain Belief

	**Model 1: Main effects with covariates**					**Model 2: Removing nonsignificant covariate(s)**					**Model 3: Adding interaction terms to the best fit model**				
	B	SE	95% CI		*P*	B	SE	95% CI		*P*	B	SE	95% CI		*P*
Intercept	-1.06	0.48	-2.01	-0.11	.029*	-0.96	0.49	-1.93	0.02	.055	-0.93	0.50	-1.91	0.05	.062
Posttraining	-0.04	0.09	-0.21	0.14	.66	-0.04	0.09	-0.21	0.14	.68	-0.07	0.15	-0.37	0.22	.63
Gamified ABMT	0.04	0.20	-0.36	0.44	.83	-0.08	0.19	-0.45	0.29	.67	-0.08	0.20	-0.48	0.32	.69
Standard ABMT	0.41	0.18	0.06	0.77	.024*	0.42	0.18	0.07	0.78	.020*	0.38	0.20	0.00	0.77	.051
Primary[a]	1.57	0.79	0.00	3.14	.051	-	-	-	-	-	-	-	-	-	-
Secondary[a]	0.02	0.19	-0.35	0.39	.93	-	-	-	-	-	-	-	-	-	-
Single[a]	-0.26	0.17	-0.59	0.08	.14	-	-	-	-	-	-	-	-	-	-
Divorced or separated[a]	-0.04	0.22	-0.47	0.40	.86	-	-	-	-	-	-	-	-	-	-
Student[a]	-0.37	0.26	-0.88	0.14	.16	-	-	-	-	-	-	-	-	-	-
Retired[a]	-0.14	0.20	-0.54	0.26	.49	-	-	-	-	-	-	-	-	-	-
Not employed[a]	0.30	0.19	-0.08	0.69	.12	-	-	-	-	-	-	-	-	-	-
Depression[a]	0.02	0.01	0.00	0.03	.028*	0.02	0.01	0.00	0.03	.041*	0.02	0.01	0.00	0.03	.044*
Post*Gamified ABMT	-	-	-	-	-						0.00	0.21	-0.42	0.42	1.00
Post*Standard ABMT	-	-	-	-	-						0.12	0.22	-0.31	0.54	.59
AIC	559.32.					558.34					561.97				
BIC	606.64					582.00					592.39				

Note. ABMT=Attentional Bias Modification Training; AIC=Akaike Information Criterion; BIC=Bayesian Information Criterion; for time, baseline was the reference category; for training condition, control was the reference category; for education level, tertiary level education was the reference category; for marital status, married or in a relationship was the reference category; for work status, employed was the reference category.

[a] Covariates.

*Statistical significance *P*<.05, two-tailed.

Table S10. Linear Mixed Models for Pain Interpretation Bias: Social Belief

	Model 1: Main effects with covariates					Model 2: Removing nonsignificant covariate(s)					Model 3: Adding interaction terms to the best fit model				
	B	SE	95% CI		*P*	B	SE	95% CI		*P*	B	SE	95% CI		*P*
Intercept	-2.52	0.65	-3.80	-1.23	<.001*	-2.62	0.64	-3.88	-1.36	<.001*	-2.61	0.64	-3.88	-1.35	<.001*
Posttraining	0.05	0.09	-0.12	0.23	.56	0.05	0.09	-0.12	0.23	.56	0.07	0.15	-0.22	0.37	.62
Gamified ABMT	-0.24	0.27	-0.78	0.30	.38	-0.27	0.24	-0.74	0.20	.26	-0.23	0.25	-0.72	0.27	.37
Standard ABMT	0.33	0.24	-0.16	0.81	.19	0.31	0.23	-0.15	0.77	.18	0.29	0.24	-0.19	0.78	.23
Primary[a]	-0.50	1.09	-2.66	1.65	.65	-	-	-	-	-	-	-	-	-	-
Secondary[a]	0.12	0.25	-0.38	0.62	.65	-	-	-	-	-	-	-	-	-	-
Single[a]	-0.06	0.23	-0.51	0.39	.79	-	-	-	-	-	-	-	-	-	-
Divorced or separated[a]	-0.15	0.30	-0.74	0.44	.61	-	-	-	-	-	-	-	-	-	-
Student[a]	0.39	0.35	-0.30	1.08	.27	-	-	-	-	-	-	-	-	-	-
Retired[a]	-0.09	0.27	-0.63	0.45	.74	-	-	-	-	-	-	-	-	-	-
Not employed[a]	0.07	0.26	-0.45	0.59	.80	-	-	-	-	-	-	-	-	-	-
Depression[a]	0.03	0.01	0.00	0.05	.021*	0.03	0.01	0.01	0.05	.007*	0.03	0.01	0.01	0.05	.007*
Post*Gamified ABMT	-	-	-	-	-	-	-	-	-	-	-0.12	0.21	-0.54	0.30	.57
Post*Standard ABMT	-	-	-	-	-	-	-	-	-	-	0.06	0.22	-0.37	0.49	.78
AIC	633.47					621.84					625.11				
BIC	680.79					645.50					655.53				

Note. ABMT=Attentional Bias Modification Training; AIC=Akaike Information Criterion BIC=Bayesian Information Criterion; for time, baseline was the reference category; for training condition, control was the reference category; for education level, tertiary level education was the reference category; for marital status, married or in a relationship was the reference category; for work status, employed was the reference category.

[a] Covariates.

*Statistical significance *P*<.05, two-tailed.

3. SENSITIVITY ANALYSES FOR PRIMARY OUTCOMES

Table S11. Linear Mixed Models for Task-Related Engagement

	**Model 1: Main effects**					**Model 2: Adding interaction terms**				
	B	SE	95% CI		*P*	B	SE	95% CI		*P*
Intercept	6.25	0.44	5.38	7.11	<.001*	6.12	0.46	5.22	7.02	<.001*
Session 6	-1.62	0.19	-2.00	-1.25	<.001*	-1.40	0.31	-2.01	-0.78	<.001*
Session 5	-1.33	0.19	-1.71	-0.95	<.001*	-1.04	0.32	-1.66	-0.41	.001*
Session 4	-1.28	0.19	-1.65	-0.91	<.001*	-1.12	0.31	-1.71	-0.52	<.001*
Session 3	-0.99	0.18	-1.35	-0.63	<.001*	-1.00	0.30	-1.59	-0.41	<.001*
Session 2	-0.69	0.18	-1.04	-0.34	<.001*	-0.46	0.29	-1.04	0.11	.12
Gamified ABMT	-0.04	0.62	-1.26	1.17	.94	0.09	0.66	-1.22	1.39	.90
Standard ABMT	-0.46	0.61	-1.67	0.75	.45	-0.18	0.66	-1.47	1.12	.79
Session 6*Gamified ABMT	-	-	-	-	-	-0.41	0.45	-1.29	0.47	.36
Session 6*Standard ABMT	-	-	-	-	-	-0.29	0.47	-1.22	0.63	.53
Session 5*Gamified ABMT	-	-	-	-	-	-0.62	0.46	-1.52	0.29	.18
Session 5*Standard ABMT	-	-	-	-	-	-0.32	0.47	-1.24	0.61	.50
Session 4*Gamified ABMT	-	-	-	-	-	-0.14	0.45	-1.01	0.74	.76
Session 4*Standard ABMT	-	-	-	-	-	-0.40	0.46	-1.30	0.50	.38
Session 3*Gamified ABMT	-	-	-	-	-	0.25	0.43	-0.59	1.10	.56
Session 3*Standard ABMT	-	-	-	-	-	-0.29	0.45	-1.18	0.60	.52
Session 2*Gamified ABMT	-	-	-	-	-	-0.14	0.42	-0.96	0.68	.74
Session 2*Standard ABMT	-	-	-	-	-	-0.60	0.44	-1.46	0.26	.17
AIC	2388.68					2401.20				
BIC	2432.53					2488.91				

Note. ABMT=Attentional Bias Modification Training; AIC=Akaike Information Criterion; BIC=Bayesian Information Criterion; for session, session 1 was the reference category; for training condition, control was the reference category.

*Statistical significance *P*<.05, two-tailed.

Table S12. Linear Mixed Models for Pain Intensity

	Model 1: Main effects					Model 2: Adding interaction terms				
	B	SE	95% CI		*P*	B	SE	95% CI		*P*
Intercept	66.04	1.01	64.05	68.02	<.001*	65.86	1.10	63.70	68.02	<.001*
Follow-up	-1.87	0.72	-3.28	-0.46	.010*	-1.33	1.17	-3.63	0.97	.26
Posttraining	-0.29	0.68	-1.63	1.06	.68	-0.13	1.14	-2.38	2.12	.91
Gamified ABMT	-1.68	1.37	-4.40	1.04	.22	-0.34	1.59	-3.47	2.78	.83
Standard ABMT	0.94	1.37	-1.76	3.64	.49	0.20	1.57	-2.89	3.29	.90
Follow*Gamified ABMT	-	-	-	-	-	-2.74	1.71	-6.11	0.63	.11
Follow*Standard ABMT	-	-	-	-	-	1.08	1.70	-2.27	4.43	.53
Post*Gamified ABMT	-	-	-	-	-	-2.07	1.62	-5.27	1.13	.20
Post*Standard ABMT	-	-	-	-	-	1.72	1.65	-1.53	4.98	.30
AIC	2129.77					2130.72				
BIC	2156.17					2172.20				

Note. ABMT=Attentional Bias Modification Training; AIC=Akaike Information Criterion; BIC=Bayesian Information Criterion; for time, baseline was the reference category; for training condition, control was the reference category.
*Statistical significance *P*<.05, two-tailed.

Table S13. Linear Mixed Models for Pain Interference

	Model 1: Main effects					Model 2: Adding interaction terms				
	B	SE	95% CI		*P*	B	SE	95% CI		*P*
Intercept	66.56	0.97	64.65	68.48	<.001*	66.88	1.04	64.84	68.92	<.001*
Follow-up	-2.05	0.62	-3.26	-0.84	.001*	-3.57	1.01	-5.55	-1.58	<.001*
Posttraining	-0.75	0.59	-1.91	0.40	.20	-0.47	0.99	-2.41	1.48	.64
Gamified ABMT	-2.53	1.34	-5.18	0.12	.061	-3.15	1.50	-6.11	-0.20	.037*
Standard ABMT	-0.52	1.33	-3.16	2.11	.69	-0.88	1.48	-3.80	2.04	.55
Follow*Gamified ABMT	-	-	-	-	-	2.88	1.48	-0.03	5.79	.053
Follow*Standard ABMT	-	-	-	-	-	1.95	1.47	-0.95	4.84	.19
Post*Gamified ABMT	-	-	-	-	-	-0.35	1.40	-3.11	2.41	.80
Post*Standard ABMT	-	-	-	-	-	-0.51	1.43	-3.32	2.31	.72
AIC	2062.80					2064.90				
BIC	2089.20					2106.39				

Note. ABMT=Attentional Bias Modification Training; AIC=Akaike Information Criterion; BIC=Bayesian Information Criterion; for time, baseline was the reference category; for training condition, control was the reference category.
*Statistical significance *P*<.05, two-tailed.

Table S14. One-Way Analysis of Variance Results for Interest and Enjoyment (Intrinsic Motivation Inventory) and Total Number of Training Sessions Completed

Variable	*F*	*P*	η_p^2
Interest and enjoyment	1.28	.28	.025
Number of completed sessions	1.56	.22	.024

4. SUMMARY STATISTICS FOR LINEAR MIXED MODEL ANALYSES

Table S15. Summary Statistics on Outcome Measures by Training Condition Across Time

Variable	Standard ABMT			Gamified ABMT			Control		
	Obs	M	SD	Obs	M	SD	Obs	M	SD
Engagement	181	5.09	3.20	200	5.24	2.97	212	5.75	2.92
Pain intensity	104	66.22	8.02	104	63.62	7.10	113	65.16	7.28
Pain interference	104	65.08	7.49	104	63.16	6.65	113	65.43	7.31
Attentional bias index	66	0.48	17.23	71	-0.47	21.76	75	0.08	20.27
Anxiety	104	60.17	11.19	104	57.88	9.31	113	59.98	8.25
Depression	104	61.26	10.55	104	56.06	10.15	113	60.36	9.03
Perceived improvement	61	4.05	0.90	63	4.00	0.67	68	3.91	0.94
Interpretation bias: health belief	70	0.45	1.06	71	-0.14	0.88	76	-0.01	0.87
Interpretation bias: social belief	70	-0.66	1.37	71	-1.31	1.12	76	-0.93	0.98

Note. Obs=observations.

Table S16. Summary Statistics on Outcome Measures by Assessment Time Points

Variable	Baseline			Posttraining			Follow-up		
	n	M	SD	n	M	SD	n	M	SD
Pain intensity	129	65.81	6.83	102	65.21	7.62	90	63.61	8.22
Pain interference	129	65.58	6.83	102	64.54	7.27	90	63.19	7.53
Attentional bias index	125	-0.95	21.15	87	1.42	17.76	-	-	-
Anxiety	129	59.74	9.61	102	59.60	9.97	90	58.55	9.39
Depression	129	60.13	9.95	102	58.45	10.21	90	58.91	10.29
Perceived improvement	-	-	-	102	4.01	0.76	90	3.96	0.94
Interpretation bias: health belief	129	0.11	1.02	88	0.07	0.89	-	-	-
Interpretation bias: social belief	129	-0.96	1.25	88	-0.98	1.11	-	-	-

Table S17. Summary Statistics on Engagement Measure by Assessment Time Points

Variable	n	M	SD
Engagement: Session 1	118	6.00	2.90
Engagement: Session 2	110	5.54	3.04
Engagement: Session 3	100	5.29	3.09
Engagement: Session 4	92	5.26	3.02
Engagement: Session 5	85	5.13	3.02
Engagement: Session 6	88	4.78	3.07

Note. Item was rated on an 11-point Likert scale, ranging from 0 (*not at all*) to 10 (*very much*)
